# Marked point process variational autoencoder with applications to unsorted spiking activities

**DOI:** 10.1371/journal.pcbi.1012620

**Published:** 2024-12-30

**Authors:** Ryohei Shibue, Tomoharu Iwata

**Affiliations:** 1 Communication Science Laboratories, NTT Corporation, Kanagawa, Japan; 2 Communication Science Laboratories, NTT Corporation, Kyoto, Japan; Northwestern University Feinberg School of Medicine, UNITED STATES OF AMERICA

## Abstract

Spike train modeling across large neural populations is a powerful tool for understanding how neurons code information in a coordinated manner. Recent studies have employed marked point processes in neural population modeling. The marked point process is a stochastic process that generates a sequence of events with marks. Spike train models based on such processes use the waveform features of spikes as marks and express the generative structure of the unsorted spikes without applying spike sorting. In such modeling, the goal is to estimate the joint mark intensity that describes how observed covariates or hidden states (e.g., animal behaviors, animal internal states, and experimental conditions) influence unsorted spikes. A major issue with this approach is that existing joint mark intensity models are not designed to capture high-dimensional and highly nonlinear observations. To address this limitation, we propose a new joint mark intensity model based on a variational autoencoder, capable of representing the dependency structure of unsorted spikes on observed covariates or hidden states in a data-driven manner. Our model defines the joint mark intensity as a latent variable model, where a neural network decoder transforms a shared latent variable into states and marks. With our model, we derive a new log-likelihood lower bound by exploiting the variational evidence lower bound and upper bound (e.g., the *χ* upper bound) and use this new lower bound for parameter estimation. To demonstrate the strength of this approach, we integrate our model into a state space model with a nonlinear embedding to capture the hidden state dynamics underlying the observed covariates and unsorted spikes. This enables us to reconstruct covariates from unsorted spikes, known as neural decoding. Our model achieves superior performance in prediction and decoding tasks for synthetic data and the spiking activities of place cells.

## Author summary

Recent neural recording techniques provide simultaneous insights into large neural population activities. This type of data may offer new insights into the mechanisms of high-dimensional and highly nonlinear neural coding, which are not available with small data. The marked point process is a promising tool for modeling neural population activities. This model avoids the preprocessing of spike assignment and directly estimates the generative distribution of neural recordings. However, when analyzing high-dimensional data or nonlinear dynamics underlying the data, developing appropriate parametric models for the marked point process becomes challenging. To address this limitation, we propose a new marked point process model that uses a variational autoencoder. By utilizing the strengths of neural networks, our model automatically estimates the spike generation distribution from neural recordings, enabling us to find its dependence on animal behavior or external stimuli. Our model is also capable of performing neural decoding, a fundamental technique used to reconstruct variables in the outside world from neural population activities. By integrating our model with neural network-based state space models, we can reconstruct animal behavior or external stimuli from unsorted spikes in a data-driven manner. This could help researchers formulate hypotheses regarding the connection between neural population activities and other influencing factors, and design further experiments and analyses.

## 1 Introduction

Recent techniques have enabled the simultaneous recording of neural activities from a large number of neurons. For instance, high-density probe arrays can measure neuron spiking activities across multiple brain regions [1, 2]. These datasets offer new insights into how neurons code information in a coordinated manner.

With the development of neural recording techniques, there has been a growing need for analysis methods for large neural population activities. Marked point processes have emerged as a valuable tool for this purpose. A marked point process is a stochastic process that generates a sequence of events, each accompanied by feature values, called marks. This process has been used for statistical modeling of various data types, including earthquakes [3], financial transactions [4], social networks [5], recommendations [6], and violent crimes [7].

This paper proposes a marked point process model that expresses the generative distribution of unsorted spikes. Unsorted spikes refer to sequences of pairs consisting of spike times and spike features. We assume that there is a state that influences the occurrences of unsorted spikes. This state could be any kind of information that affects neural population activities, including observable covariates (e.g., animal behavior, experimental conditions) and hidden states (e.g., animal internal state). Under this assumption, our objective is to estimate the joint mark intensity function defined in the product space of the state and the spike feature. This function encodes the probability of the occurrence of a spike with certain features, such as spike waveform.

**Fig 1:**
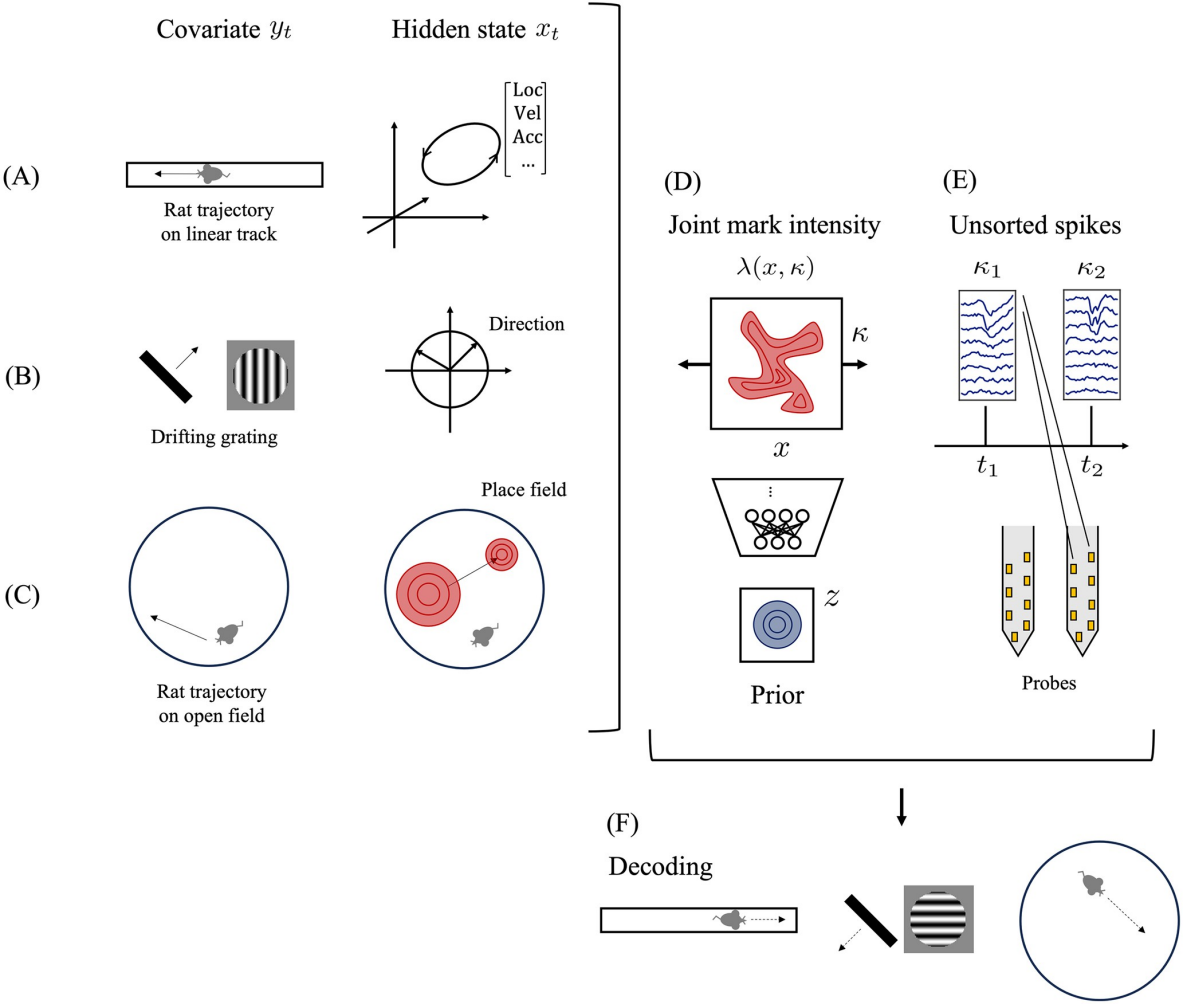
Illustration of our model. The left panels show examples of covariates *y*_*t*_ and hidden states *x*_*t*_. (A) Place cell of a rat foraging on a linear track. The firing rate of this neuron depends not only on the rat’s location but also on the rat’s movement direction. The higher-dimensional representation, including rat direction information, is essential to capturing spiking activities. (B) V1 neuron of a monkey watching gratings. The V1 neuron responds to the orientation of the gratings. The lower-dimensional representation, including only the orientation information, is sufficient to capture spiking activities. (C) Plasticity of place cell. The location of the place field (red) changes during the experiment, and this shift is not directly observed. Expressing this shift as hidden state dynamics enables us to capture such plasticity. (D) Joint mark intensity model. This function determines the relationship between state *x*_*t*_ and unsorted spikes {(*t*_*i*_, *κ*_*i*_)}. Our model consists of a neural network that transforms a simple prior over latent variable *z* into a joint mark intensity. (E) Unsorted spikes. This sequence consists of spike times *t*_*i*_ and feature values *κ*_*i*_ that characterize the spikes (e.g., spike amplitude, spike waveform). (F) Decoding. Reconstructing the covariate *y*_*t*_ from the unsorted spikes and the estimated joint mark intensity shows the relationship between neural data and animal behavior.

We here describe the usage of the joint mark intensity function for neural population modeling [8–15]. During multi-unit recording, probes are inserted into the brain to measure electrical potentials generated by nearby neurons. In conventional point process modeling (e.g., [16]), the initial step is spike sorting [17] to allocate spikes to the corresponding neurons by using spike waveform features. Subsequently, the intensity is estimated as a function of the animal’s behavioral state or external stimulus for each individual neuron. However, spike sorting classifies spikes with hard decision boundaries, and it can sometimes lead to misassignment, which has a negative impact on subsequent analyses [18]. To address this, recent studies have taken a different approach, directly estimating the joint mark intensity from electrical potential and using it for neural decoding [8–13, 15]. In this approach, a feature of a spike waveform (e.g., spike amplitude) is considered as the mark *κ*, and the unsorted spikes {(*t*_*i*_, *κ*_*i*_)}, where *t*_*i*_ denotes the spike time and *κ*_*i*_ denotes the corresponding feature of the *i*-th spike, are considered as observations (Fig 1E). The state that influences these unsorted spikes, such as an animal’s behavior, experimental conditions, or the animal’s internal state, is denoted as *x*_*t*_. The joint mark intensity λ(*x*, *κ*) explains how this state affects the occurrence of unsorted spikes. More specifically, λ(*x*, *κ*)Δ*t* represents the expected number of spikes with the spike feature *κ* within an interval of length Δ*t* when the state takes the value *x*.

Previous studies have used Gaussian mixture models [12–15] or kernel density estimation [9–11] to estimate the joint mark intensity. Although these methods have achieved significant improvement in estimation accuracy compared to sorting-based models, their usage is limited to the case where the state *x*_*t*_ and the spike feature *κ* are embedded in a low-dimensional space. As far as we know, there are no papers addressing the problem of spike features with dimensions exceeding 10.

Recent recording techniques, such as Neuropixels [1, 2], provide larger neuron populations as high-dimensional data. If there are denser recording contacts and the number of neurons around the probes is large, more detailed waveform features are necessary to refine spike isolation [19]. A previous study on unsorted decoding applied to Neuropixel data used spike location along the probe’s width and depth, as well as the maximum peak-to-peak amplitude for spike features [20]. However, spike locations are calculated from the spike amplitude estimates at each site, which are often biased by noise [21, 22]. Preprocessing raw recordings into such low-dimensional features may lead to information loss and decreased decoding performance, and the optimal selection of spike features for unsorted decoding remains an open question. Thus, there is a growing need for a joint mark intensity model that can bypass the selection of spike features by directly processing raw spike waveforms.

In this paper, we propose a neural network-based model for joint mark intensity. Using neural networks allows us to capture the complicated relationship between states and marks without relying on prior knowledge, even when these are high-dimensional vectors. However, using a simple feedforward neural network incurs a high computational cost during parameter estimation. To avoid this problem, we incorporate a variational autoencoder (VAE) [23] into the joint mark intensity (Fig 1D). The VAE is a generative model consisting of a neural network that transforms a simple prior over latent space into the observation space. Specifically, we decompose the joint mark intensity function into a nonnegative scale parameter and a joint probability density function of states and marks, adopting the VAE for the latter density function.

We estimate the parameters of our joint mark intensity model by maximizing the point process likelihood. However, similar to VAE, the log-likelihood contains expectations over latent variables, and these terms cannot be computed in closed form. Moreover, since the point process log-likelihood contains the negative evidence term − *p*(*x*), the conventional lower bound maximization used in VAE learning is not applicable. To address this, we derive a new lower bound for the point process log-likelihood and maximize this lower bound instead. To obtain the lower bound, we unify the evidence lower bound (e.g., Rényi lower bound) and upper bound (e.g., *χ* upper bound) derived from different divergences [24–28]. Employing this lower bound enables us to find encoders that give low-variance gradient estimates during parameter optimization, leading to better joint mark intensity estimates. To the best of our knowledge, this is the first work to derive the lower bound of the point process log-likelihood by unifying two evidence bounds and to use it for parameter estimation.

Our model can estimate the joint mark intensity defined in a high-dimensional space in a data-driven manner, without relying on any prior knowledge. To emphasize this strength, we integrate our model into a state space model to capture the hidden state dynamics underlying the data. In neural population modeling, there are several cases where non-observable hidden state dynamics influence neural population activities (Fig 1A–1C). Previous works have tackled this problem by combining a state space model with a point process model [10, 29–33].

In such cases, we do not know what hidden state exists behind the data and how this state affects the occurrence of unsorted spikes beforehand. Thus, we need to estimate the embedding that projects the observations into the hidden state space. This embedding can be nonlinear when the hidden state dynamics, the generation process of unsorted spikes, or the observed covariates are nonlinear. Thus, a simple linear Gaussian state space model is sometimes not sufficient.

To handle such nonlinearity, we employ a black-box state space model with a nonlinear embedding [32, 34, 35]. These models adopt neural network-based nonlinear embeddings that transform observations to hidden states and impose linear dynamics on the hidden state space. This embedding can transform the complicated nonlinear dynamics of observations into the more manageable linear dynamics of hidden states in a data-driven manner. However, since the hidden state space is determined by neural network embeddings and thus unknown beforehand, developing the parametric models for joint mark intensity defined in this space is difficult. Even in such cases, our model can give reasonable joint mark intensity estimates.

This state space model formulation is especially useful for neural decoding (Fig 1F). Neural decoding is the reconstruction of observed covariates from the neural recordings. This task provides a clue suggesting that neural population activities have enough information to reconstruct animal behaviors or external stimuli. The joint mark intensity serves as a valuable tool for neural decoding since it directly connects these variables and neural recordings without applying spike sorting. Current neural decoding based on joint mark intensity is limited to a low-dimensional covariate with simple dynamics [9–13, 15]. By integrating our joint mark intensity model, our state space model can handle high-dimensional observations governed by highly nonlinear dynamics without any prior knowledge. To achieve efficiency, we propose optimization-based decoding with our model.

Our motivations and contributions are summarized as follows. The motivations include:

1. Proposing new models capable of handling high-dimensional spike features (e.g., raw spike waveforms), not just low-dimensional spike features (e.g., spike locations and maximum peak-to-peak amplitudes of spikes),
2. Deriving an appropriate estimation method for the new model,
3. Demonstrating the strength of the proposed model.

The contributions addressing these motivations are:

1. Expressing the joint mark intensity function of the marked point process as a variational autoencoder,
2. Combining two variational bounds to derive a new variational lower bound of the point process log-likelihood,
3. Integrating the proposed model into a black-box state space model.

We evaluate our model on synthetic data and place cell spiking activities in terms of prediction and decoding. The results show that our model achieves better performance than other marked point process models and sorting-based decoding.

## 2 Materials and methods

In this section, we describe our model. In Section 2.1, we provide an explanation of the joint mark intensity. In Section 2.2, we define our joint mark intensity model. In Section 2.3, we derive the lower bound for the point process log-likelihood under our model and explain how to use it for parameter estimation. In Section 2.4, we integrate our joint mark intensity model into a state space model with a nonlinear embedding. In Section 2.5, we demonstrate how our model can be used for decoding.

### 2.1 Preliminaries

A point process is one of the stochastic processes used to model a sequence of events. Marked point processes are a subclass of point processes that generate events with feature values called marks. When employing marked point processes for statistical modeling, the goal is to estimate the conditional intensity function that describes the probability structure of the process. This function determines the instantaneous probability of observing an event with a particular mark. The definition of the conditional intensity function is as follows [36]:

$$\begin{array}{c} \lambda \left(t,\kappa |x_{t}\right)≔\underset{\Delta t,\Delta \kappa \to 0}{\text{lim}}\frac{E[N\left(\left(t,t+\Delta t\right]\times B\left(\kappa ,\Delta \kappa \right)\right)|x_{t}]}{\Delta t\;|B(\kappa ,\Delta \kappa )|}. \end{array}$$

Here, κ∈K is a mark, *N*(⋅) is a counting measure defined on the product space of time and mark, $x_{t}\in X$ is a state that influences the occurrence of events, B(κ,Δκ)⊂K is an open ball of radius $\Delta \kappa \in R_{+}$ centered at *κ*, and |*B*(*κ*, Δ*κ*)| is the Lebesgue measure of *B*(*κ*, Δ*κ*). Although the general definition of this function depends on the history of the process, we do not consider such dependency in this paper.

To ensure estimation efficiency, we focus on a specific class of marked point processes. We assume that there is a state that changes with time, *x*_*t*_, and events are generated from a marked inhomogeneous Poisson process whose intensity is determined by *x*_*t*_. Under this assumption, the conditional intensity function can be written as

$$\begin{array}{c} \lambda \left(t,\kappa |x_{t}\right)=\lambda \left(x_{t},\kappa \right), \end{array}$$

where λ(*x*, *κ*) is a nonnegative function defined in the space X×K. As mentioned in Section 1, we refer to this function as the “joint mark intensity.” This function explains how event occurrences depend on the state. More specifically, the value λ(*x*, *κ*)Δ*t* is the expected number of events with mark *κ* occurring in an interval of length Δ*t* when the state takes the value *x*.

Suppose that events ${\left\{\left(t_{i},\kappa_{i}\right)\right\}}_{i=1}^{n}$ are generated from this marked point process within an interval (0, *T*]. Then, the log-likelihood is given by

$$\begin{array}{c} \sum_{i=1}^{n}\text{log}\;\lambda \left(x_{t_{i}},\kappa_{i}\right)-\int_{0}^{T}\int_{K}\lambda \left(x_{t},\kappa \right)\;d\kappa \;dt. \end{array}$$

In this expression, we need the continuous-time path of *x*_*t*_ to evaluate the exact log-likelihood. In practice, the values of *x*_*t*_ are available only at discrete time points *t*_*j*_ ≔ *j*Δ*t*, *j* = 1, …, *m*, where Δ*t* = *T*/*m*. Let $x_{i}≔x_{t_{i}}$ be a state value at a spike time and $x_{j}≔x_{t_{j}}$ be a state value at discrete time points. Note that we do not know the values of *x*_*i*_ since *x*_*t*_ is only measured at discrete time points ${\left\{t_{j}\right\}}_{j=1}^{m}$. A straightforward approach to obtaining *x*_*i*_ is to use nearest-neighbor interpolation from the available measurements ${\left\{x_{j}\right\}}_{j=1}^{m}$. Under this assumption, the log-likelihood is approximated as

$$\begin{array}{c} \sum_{i=1}^{n}\;\text{log}\;\lambda \left(x_{i},\kappa_{i}\right)-\sum_{j=1}^{m}\int_{K}\lambda \left(x_{j},\kappa \right)\;\Delta t\;d\kappa . \end{array}$$ (1)

In this approximation, the integral in the log-likelihood is replaced by Riemann summation. By maximizing this log-likelihood within a set of functions of λ(*x*, *κ*), we can estimate the joint mark intensity given the observations. For notational simplicity, we will omit Δ*t* in this log-likelihood without any loss of generality. For justification, see Section S1.1 in S1 Text.

### 2.2 Joint mark intensity model

In this section and the one that follows, we assume that there is an observable state *x*_*t*_ and that this state affects the occurrence of unsorted spikes. An example of *x*_*t*_ corresponds to external stimuli presented to the animal during the experiment. The goal is to estimate the joint mark intensity λ(*x*, *κ*) from the state values at spike times ${\left\{x_{i}\right\}}_{i=1}^{n}$, at discrete time points ${\left\{x_{j}\right\}}_{j=1}^{m}$, and the unsorted spikes ${\left\{\left(t_{i},\kappa_{i}\right)\right\}}_{i=1}^{n}$.

A straightforward way to express the joint mark intensity λ(*x*, *κ*) in a high-dimensional space is to use a neural network that takes pairs of *x* and *κ* as inputs and outputs nonnegative values. However, expressing λ(*x*, *κ*) as a neural network makes integration with respect to *κ* in the log-likelihood (1) challenging.

To address this issue, we propose a new joint mark intensity model based on neural networks. We first decompose the joint mark intensity λ(*x*, *κ*) into a base intensity term and a joint probability density of the state and mark, and define the latter density function by a variational autoencoder:

$$\begin{array}{c} \lambda \left(x,\kappa \right)=\lambda_{0}\;\int p\left(x,\kappa |z\right)\;p\left(z\right)\;dz, \end{array}$$

where $\lambda_{0}\in R_{+}$ is a scale parameter, *z* is a low-dimensional representation of the state and mark, *p*(*x*, *κ* ∣ *z*) is a conditional density referred to as a decoder, and *p*(*z*) is a prior. We define *p*(*x*, *κ* ∣ *z*) as a distribution family whose parameters are neural networks with *z* as input.

Substituting this definition into the log-likelihood (1), we obtain

$$\begin{array}{c} \sum_{i=1}^{n}\;\text{log}\;(\lambda_{0}\int p\left(x_{i},\kappa_{i}|z\right)\;p\left(z\right)\;dz)-\sum_{j=1}^{m}\lambda_{0}\int p\left(x_{j}|z\right)\;p\left(z\right)\;dz, \end{array}$$ (2)

where *p*(*x* ∣ *z*) is the marginal density of *x* given *z*:

$$\begin{array}{c} p(x|z)=\int p(x,\kappa |z)\;d\kappa . \end{array}$$

This model formulation has several advantages over existing models. First, it can represent highly complex data structures due to its use of neural networks. Second, since the integral with respect to *κ* in the log-likelihood becomes a closed form with our model, the log-likelihood can be calculated without heavy numerical integration when dim(*z*) ≪ dim(*κ*).

This log-likelihood still contains integration terms with respect to the latent variable *z*, and a simple Monte Carlo approximation of these terms results in large variance in gradient estimates [23]. To reduce this variance, we introduce two encoders, *q*(*z* ∣ *x*, *κ*) and *q*(*z* ∣ *x*), and derive the lower bound of this log-likelihood as explained in the next section.

### 2.3 Lower bound for point process log-likelihood

To obtain the lower bound for the point process log-likelihood (2), we provide the lower bound for

$$\begin{array}{c} \text{log}\;p(x,\kappa )=\text{log}\;\int p(x,\kappa |z)\;p(z)\;dz \end{array}$$

and the upper bound for

$$\begin{array}{c} p(x)=\int p(x|z)\;p(z)\;dz. \end{array}$$

The main idea throughout this section is to rewrite the above evidence as a function of the divergence term and the evidence bound term. Based on the rewritten expression, minimizing divergence has the effect of tightening the evidence bound to the evidence. For step-by-step derivations of the bounds, see Section S1.2 in S1 Text. The lower bound introduced in this section can be generalized under a subclass of the *f*-divergence family [28]. For the details, see Section S1.3 in S1 Text.

First, we derive the lower bound for the log evidence log *p*(*x*, *κ*). This type of lower bound has been widely used in variational inference, e.g., variational autoencoders [23]. Here, we present the Rényi lower bound [24] as an example. The Rényi lower bound is an extension of the evidence lower bound based on the Kullback-Leibler divergence. Although this lower bound was originally derived from the Rényi divergence in [24], we use *α*-divergence [37] instead to ensure consistency with subsequent discussions. The definition of the *α*-divergence with a parameter *α* ∈ (0, 1) is

$$\begin{array}{c} H_{\alpha }\left(q(z)\|p(z)\right)=-\frac{1}{\alpha }\int q{\left(z\right)}^{1-\alpha }p{\left(z\right)}^{\alpha }\;dz+\frac{1}{\alpha }. \end{array}$$

Using this divergence, we obtain the following inequality:

$$\begin{array}{cc} p(x,\kappa ) & \geq {\left(p{\left(x,\kappa \right)}^{\alpha }-\alpha \;p{\left(x,\kappa \right)}^{\alpha }\;H_{\alpha }\left(q(z|x,\kappa )\|p(z|x,\kappa )\right)\right)}^{\frac{1}{\alpha }}\\ & ={\left(E_{q(z|x,\kappa )}[{\left(\frac{p(x,\kappa |z)\;p(z)}{q(z|x,\kappa )}\right)}^{\alpha }]\right)}^{\frac{1}{\alpha }}. \end{array}$$

Here, *p*(*z* ∣ *x*, *κ*) is the posterior of *z* given *x* and *κ*,

$$\begin{array}{c} p\left(z|x,\kappa \right)=\frac{p(x,\kappa |z)\;p(z)}{p(x,\kappa )}, \end{array}$$

and *q*(*z* ∣ *x*, *κ*) is an encoder. The inequality is derived from the nonnegativity of the *α*-divergence.

Maximizing this lower bound with respect to the encoder reduces to a minimization of

$$\begin{array}{c} H_{\alpha }\left(q(z|x,\kappa )\|p(z|x,\kappa )\right). \end{array}$$

Note that equality holds when *q*(*z* ∣ *x*, *κ*) = *p*(*z* ∣ *x*, *κ*). Taking the logarithm of both sides of this inequality yields the Rényi lower bound for the log evidence:

$$\begin{array}{c} \text{log}\;p\left(x,\kappa \right)\geq \frac{1}{\alpha }\;\text{log}\;(E_{q(z|x,\kappa )}[{\left(\frac{p(x,\kappa |z)\;p(z)}{q(z|x,\kappa )}\right)}^{\alpha }]). \end{array}$$

The parameter *α* controls the tightness of the bound. When *α* = 1, the bound is tightest and is equal to the objective in the importance-weighted autoencoder [38]. When *α* → 0, the bound reduces to the Kullback-Leibler lower bound used in the original variational autoencoder [23].

Next, we derive the upper bound for the evidence *p*(*x*). In the context of model learning, the evidence upper bound [24, 25] is less frequently used than the evidence lower bound. This is because the sign of the evidence in well-known objectives is inconsistent with upper bound minimization. In other words, the evidence should be larger when the model is better, but upper bound minimization results in smaller evidence. To use upper bound minimization for VAE learning, *χ*-VAE [39] adopts a two-step procedure for evidence maximization. The first step minimizes the upper bound of the evidence with respect to the encoder while fixing the decoder. The second step maximizes the evidence with respect to the decoder while fixing the encoder. This alternating optimization aims to find saddle points and tends to be unstable in practice. In contrast, maximizing the evidence lower bound achieves a better decoder and encoder simultaneously.

An illustrative application of evidence upper bound minimization for model learning can be found in undirected graphical model learning [40]. To highlight the connection to our model, we review the content of this paper. An undirected graphical model, such as a Markov random field, is defined by its unnormalized density, known as the energy function. Let $\tilde{p}\left(x,\theta \right)$ be this unnormalized density governed by parameter *θ*. Then, the log-likelihood given the observations ${\left\{x_{i}\right\}}_{i=1}^{n}$ is

$$\begin{array}{c} \sum_{i=1}^{n}\;\text{log}\;\tilde{p}\left(x_{i},\theta \right)-n\;\text{log}\;(\int \tilde{p}\left(x,\theta \right)\;dx). \end{array}$$

The integral in the second term is the partition function and is not analytically tractable in practice. To handle this integral, the authors of the paper introduced a proposal distribution *q*(*x*, *ϕ*) and derived the lower bound using the *χ* upper bound as

$$\begin{array}{c} \sum_{i=1}^{n}\;\text{log}\;\tilde{p}\left(x_{i},\theta \right)-\frac{n}{2}\;\text{log}\;(\int {\left(\frac{\tilde{p}\left(x,\theta \right)}{q(x,\phi )}\right)}^{2}\;q\left(x,\phi \right)\;dx). \end{array}$$

The parameters *θ* and *ϕ* are simultaneously estimated by maximizing the Monte Carlo estimate of this bound. In this case, the sign of the *χ* upper bound term is negative in the lower bound. Thus, upper bound minimization is consistent with likelihood maximization.

Since the point process log-likelihood contains the negative evidence −*p*(*x*), the minimization of the evidence upper bound is consistent with the maximization of the overall log-likelihood. We adopt the *χ* upper bound [25] derived from the *χ*-divergence to bound this evidence. The definition of the *χ*-divergence with a parameter *β* > 1 is

$$\begin{array}{c} \chi_{\beta }\left(q(z)\|p(z)\right)=\frac{1}{\beta }\int q{\left(z\right)}^{1-\beta }p{\left(z\right)}^{\beta }\;dz-\frac{1}{\beta }. \end{array}$$

Based on this divergence, we obtain the following inequality:

$$\begin{array}{cc} p(x) & \leq {\left(p{\left(x\right)}^{\beta }+\beta \;p{\left(x\right)}^{\beta }\;\chi_{\beta }\left(q(z|x)\|p(z|x)\right)\right)}^{\frac{1}{\beta }}\\ & ={\left(E_{q(z|x)}[{\left(\frac{p(x|z)\;p(z)}{q(z|x)}\right)}^{\beta }]\right)}^{\frac{1}{\beta }}. \end{array}$$

Here, *p*(*z* ∣ *x*) is the posterior of *z* given *x*, and *q*(*z* ∣ *x*) is another encoder. The inequality is derived from the nonnegativity of the *χ*-divergence. This inequality is also derived from Jensen’s inequality.

The minimization of this upper bound with respect to the encoder reduces to the minimization of

$$\begin{array}{c} \chi_{\beta }\left(q(z|x)\|p(z|x)\right). \end{array}$$

Equality holds when *q*(*z* ∣ *x*) = *p*(*z* ∣ *x*). The parameter *β* controls how the encoder approximates the true posterior. When *β* = 2, the encoder that minimizes this upper bound also minimizes the variance of the Monte Carlo estimate of the evidence *p*(*x*)

$$\begin{array}{c} \sum_{l=1}^{L}\frac{p\left(x|z_{l}\right)\;p\left(z_{l}\right)}{q(z_{l}|x)}, \end{array}$$

where ${\left\{z_{l}\right\}}_{l=1}^{L}$ are i.i.d. samples from *q*(*z* ∣ *x*).

Substituting these two bounds gives the lower bound of the log-likelihood (2):

$$\sum_{i=1}^{n}\frac{1}{\alpha }\;\text{log}\;(E_{q(z|x_{i},\kappa_{i})}[{\left(\frac{\lambda_{0}\;p\left(x_{i},\kappa_{i}|z\right)\;p\left(z\right)}{q(z|x_{i},\kappa_{i})}\right)}^{\alpha }])-\sum_{j=1}^{m}{\left(E_{q(z|x_{j})}[{\left(\frac{\lambda_{0}\;p\left(x_{j}|z\right)\;p\left(z\right)}{q(z|x_{j})}\right)}^{\beta }]\right)}^{\frac{1}{\beta }}.$$

In this equation, we move λ_0_ into the inner brackets. In the training step, the expectations with respect to the encoders are replaced by Monte Carlo estimates using the reparameterization trick:

$$\sum_{i=1}^{n}\frac{1}{\alpha }\;\text{log}\;(\frac{1}{L}\sum_{l=1}^{L}{\left(\frac{\lambda_{0}\;p\left(x_{i},\kappa_{i}|z_{il}\right)\;p\left(z_{il}\right)}{q(z_{il}|x_{i},\kappa_{i})}\right)}^{\alpha })-\sum_{j=1}^{m}{\left(\frac{1}{L}\sum_{l=1}^{L}{\left(\frac{\lambda_{0}\;p\left(x_{j}|z_{jl}\right)\;p\left(z_{jl}\right)}{q(z_{jl}|x_{j})}\right)}^{\beta }\right)}^{\frac{1}{\beta }},$$ (3)

where ${\left\{z_{il}\right\}}_{l=1}^{L}$ and ${\left\{z_{jl}\right\}}_{l=1}^{L}$ are i.i.d. samples from *q*(*z* ∣ *x*_*i*_, *κ*_*i*_) and *q*(*z* ∣ *x*_*j*_), respectively.

We define the decoder and encoders as distribution families whose parameters are neural networks. Specific choices are Gaussian distributions whose mean vectors and precision matrices are modeled by neural networks:

$$\begin{array}{cc} p(x,\kappa |z) & =N\left(x|\mu_{\theta }^{z\to x}\left(z\right),\Lambda_{\theta }^{z\to x}\left(z\right)\right)\;N\left(\kappa |\mu_{\theta }^{z\to \kappa }\left(z\right),\Lambda_{\theta }^{z\to \kappa }\left(z\right)\right),\\ p(z) & =N(z|0,I),\\ q(z|x,\kappa ) & =N(z|\nu_{\phi }^{x\kappa \to z}\left(x,\kappa \right),\Xi_{\phi }^{x\kappa \to z}\left(x,\kappa \right)),\\ q(z|x) & =N(z|\nu_{\phi }^{x\to z}\left(x\right),\Xi_{\phi }^{x\to z}\left(x\right)). \end{array}$$

Here, N(⋅ ∣ *μ*, Λ) is a multivariate Gaussian density function with mean *μ* and precision matrix Λ. The neural networks *μ*_*θ*_(⋅), *ν*_*ϕ*_(⋅) return mean vectors, and Λ_*θ*_(⋅), Ξ_*ϕ*_(⋅) return precision matrices. For notational clarity, we indicate the input and output domain of each neural network in the superscript of the variable. For example, the neural network $\mu_{\theta }^{z\to x}\left(z\right)$ receives *z* and returns the mean vector defined on the space of *x*. In these networks, *θ* denotes the parameters of the decoders and *ϕ* denotes the parameters of the encoders.

To achieve estimation efficiency, we define the joint density *p*(*x*, *κ* ∣ *z*) as the product of two decoders such that *x* and *κ* are conditionally independent given *z*. This definition enables us to easily calculate *p*(*x* ∣ *z*) in the *χ* upper bound. In the above Gaussian case, *p*(*x* ∣ *z*) simply reduces to $N(x|\mu_{\theta }^{z\to x}\left(z\right),\Lambda_{\theta }^{z\to x}\left(z\right))$.

The encoders *q*(*z* ∣ *x*, *κ*) and *q*(*z* ∣ *x*) approximate the posteriors *p*(*z* ∣ *x*, *κ*) and *p*(*z* ∣ *x*), respectively, and thus the optimal *q*(*z* ∣ *x*) is determined by the optimal *q*(*z* ∣ *x*, *κ*) as

$$\begin{array}{c} q(z|x)=\int q(z|x,\kappa )\;p(\kappa )\;d\kappa . \end{array}$$

To simplify parameter estimation, we use different neural networks for these encoders. Even in this approach, we do not need the preceding constraint; if the encoders *q*(*z* ∣ *x*, *κ*) and *q*(*z* ∣ *x*) are optimal, then this constraint automatically holds. On the other hand, developing encoders that satisfy this constraint reduces the model candidates and may help estimation efficiency and accuracy. Finding appropriate encoders for this purpose is our future work.

To estimate the parameters, we maximize the Monte Carlo estimate of the lower bound (3) with respect to the decoder parameter *θ*, the encoder parameter *ϕ*, and the scale parameter λ_0_ using a stochastic gradient ascent algorithm. However, since the 1/*β*-th power function in the *χ* upper bound term is concave, this Monte Carlo estimate is a biased estimator and its expectation is not guaranteed to be a lower bound for the log-likelihood (2). If *L* → ∞, this Monte Carlo estimate converges to the true value [24]. When *L* is not sufficiently large, this bias may make parameter estimation unstable: the Monte Carlo estimate sometimes diverges to zero.

To address this issue, we utilize jackknife resampling [41] and doubly reparameterized gradients [42]. The jackknife estimator is a leave-one-out resampling method that eliminates the *O*(*L*^−1^) bias. This estimator improves the order of convergence from *O*(*L*^−1^) to *O*(*L*^−2^); however, it increases the variance [41]. The doubly reparameterized gradients reduce this variance. For the details, see Sections S1.4 and S1.5 in S1 Text.

### 2.4 State space representation

In Sections 2.2 and 2.3, we discussed joint mark intensity estimation given the observable state and unsorted spikes. Hereafter, we assume that a hidden state with some dynamics exists behind this data, and that the observed covariate and unsorted spikes are generated from distributions determined by this hidden state. We denote the observed covariate as *y*_*t*_ and the hidden state as *x*_*t*_. In neural population modeling, *y*_*t*_ includes all observable values other than unsorted spikes during experiments, and *x*_*t*_ includes the values not directly observed through the experiments.

With this assumption, the model we aim to estimate is divided into three parts. The first part is the dynamics model of the hidden state. The second part is the observation model of the observed covariate given the hidden state. The last part is the marked point process model for unsorted spikes determined by the hidden state.

To consider hidden state dynamics in the discrete-time domain, we split the time axis into *R* equal-length bins and let *x*_*r*_, *r* = 1, …, *R* be the hidden states and *y*_*r*_, *r* = 1, …, *R* be the observed covariate values for each bin. We put *n*_*r*_ spikes within the *r*-th bin together and denote their marks as *κ*_*ri*_, *i* = 1, …, *n*_*r*_. In the place cell example, *x*_*r*_ is the hidden representation reflecting detailed information about the rat, *y*_*r*_ is the coordinate of the rat’s location, and *κ*_*ri*_ is a spike feature of the *i*-th spike occurring in the *r*-th bin. Hereafter, we denote $x={\left\{x_{r}\right\}}_{r=1}^{R}$, $y={\left\{y_{r}\right\}}_{r=1}^{R}$, and $\kappa ={\left\{\kappa_{r}\right\}}_{r=1}^{R}={\left\{{\left\{\kappa_{ri}\right\}}_{i=1}^{n_{r}}\right\}}_{r=1}^{R}$. Some bins may not contain any spikes, and so we define ***κ***_*r*_ = ∅ for such bins. Note that we only observe ***y*** and ***κ***, and we do not have access to information about ***x***. Thus, the dimension of *x*_*r*_ is an unknown hyperparameter and needs to be decided beforehand.

This state space representation is similar to the black-box state space model used in [32]. The main difference between our model and this work lies in the observation model. In [32], observations were the number of spikes, and the observation model was a Poisson distribution. In our model, the observations consist of the covariate and unsorted spikes, and the observation model for the latter is the marked point process.

**Fig 2:**
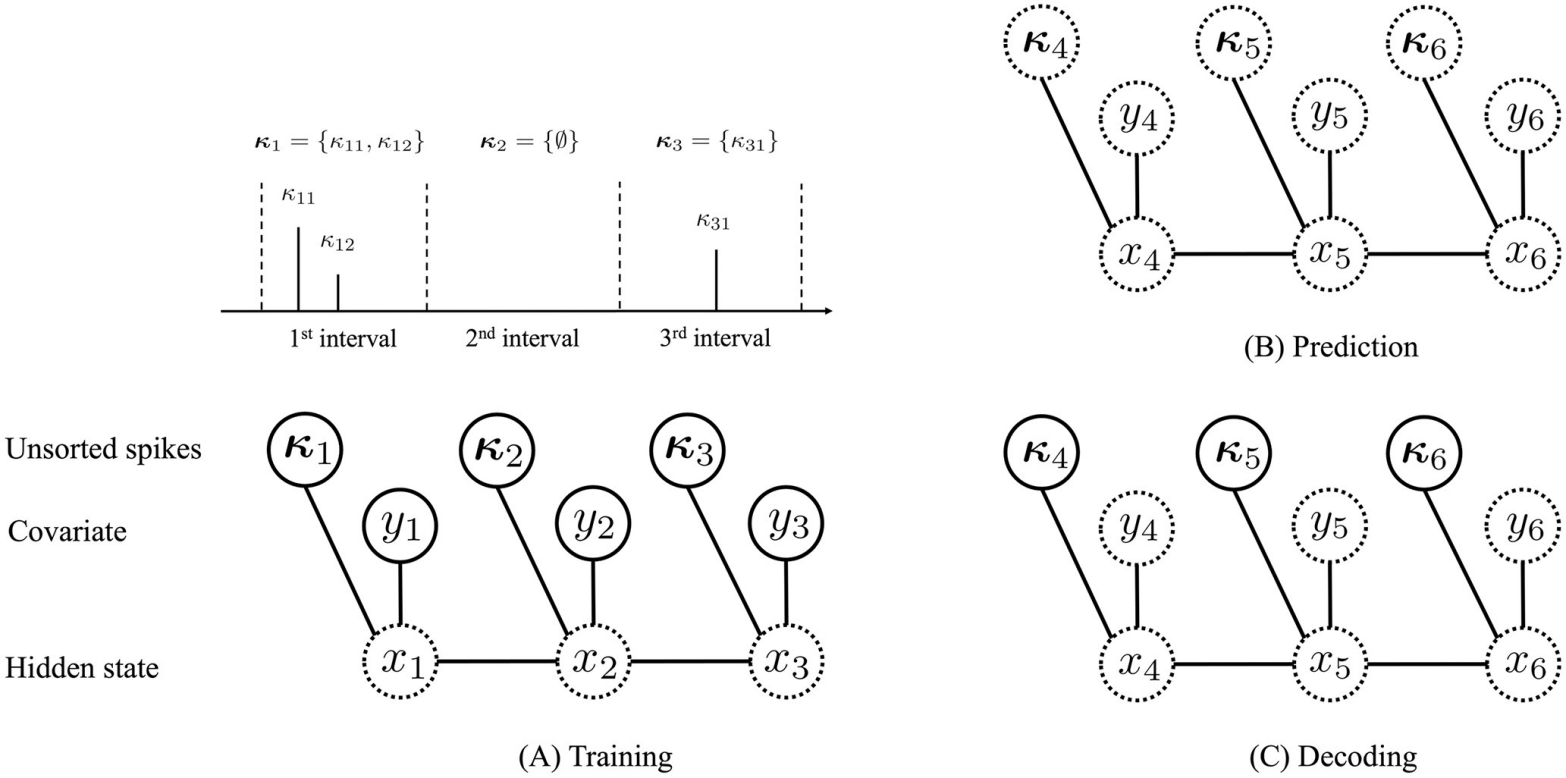
Illustration of our state space representation. Circles indicate the variables, and lines connecting these circles indicate the probabilistic dependencies. The variables surrounded by solid circles are observed variables in the training step and evaluation tasks. We assume that the hidden state *x*_*t*_ influences the observed covariate *y*_*t*_ and unsorted spikes {(*t*_*i*_, *κ*_*i*_)}. To consider the state dynamics in the discrete domain, we divide the time axis into *R* bins of equal length and define *x*_*r*_, *r* = 1, …, *R* as the hidden state and *y*_*r*_, *r* = 1, …, *R* as the observed covariate for each bin. We group *n*_*r*_ spikes within the *r*-th bin and denote their marks as $\kappa_{r}={\left\{\kappa_{ri}\right\}}_{i=1}^{n_{r}},\;r=1,\ldots ,R$. The hidden state *x*_*r*_ follows linear dynamics, and the observed covariate *y*_*r*_ depends on this state through a nonlinear embedding. The unsorted spikes ***κ***_*r*_ follow the marked point process defined by our joint mark intensity model. (A) In the training step, we estimate the parameters of our model by maximizing the lower bound of the log-likelihood given ***y*** and ***κ***. With the estimated parameters, we evaluate our model in prediction and decoding tasks. (B) In the prediction task, we evaluate the negative log-likelihood (NLL) of ***y*** and ***κ*** in the test part of the data. (C) In the decoding task, we reconstruct the covariate ***y*** from unsorted spikes ***κ*** and calculate the mean squared error (MSE) between the reconstructed covariate and the true one.

We assume that ***x***, ***y***, ***κ*** follow the state space model:

$$\begin{array}{cc} p(x,y,\kappa ) & =p(x)\;p(y|x)\;p(\kappa |x)\\ & =p\left(x_{1}\right)\prod_{r=2}^{R}p\left(x_{r}|x_{r-1}\right)\prod_{r=1}^{R}p\left(y_{r}|x_{r}\right)\prod_{r=1}^{R}p\left(\kappa_{r}|x_{r}\right), \end{array}$$

where *p*(*x*_1_) and *p*(*x*_*r*_ ∣ *x*_*r*−1_) are the initial distribution and transition distribution for *x*_*r*_, and *p*(*y*_*r*_ ∣ *x*_*r*_) and *p*(***κ***_*r*_ ∣ *x*_*r*_) are the observation distributions for *y*_*r*_ and ***κ***_*r*_, respectively. Fig 2 shows an illustration of this state space model. For estimation simplicity, we define these distributions as Gaussian distributions with neural networks [34]:

$$\begin{array}{cc} p(x_{1}) & =N(x_{1}|a_{1},V_{1}),\\ p(x_{r}|x_{r-1}) & =N(x_{r}|Fx_{r-1}+a,V),\\ p(y_{r}|x_{r}) & =N(y_{r}|\mu_{\theta }^{x\to y}\left(x_{r}\right),\Lambda_{\theta }^{x\to y}\left(x_{r}\right)), \end{array}$$

where *a*_1_ is the initial mean, *V*_1_ is the initial precision matrix, *F* is the transition matrix, *a* is the transition offset, *V* is the transition precision matrix, and $\mu_{\theta }^{x\to y}\left(x\right),\;\Lambda_{\theta }^{x\to y}\left(x\right)$ are the mean vector and precision matrix expressed as neural networks. For ***κ***_***r***_, we define the observation distribution as the point process, as described in the preceding sections:

$$\begin{array}{c} p\left(\kappa_{r}|x_{r}\right)\propto (\prod_{i=1}^{n_{r}}\lambda \left(x_{r},\kappa_{ri}\right))\text{exp}\;(-\int_{K}\lambda \left(x_{r},\kappa \right)\;d\kappa ). \end{array}$$

Under this assumption, the marginal log-likelihood

$$\begin{array}{c} \;\text{log}\;p(y,\kappa )=\;\text{log}\;\int p(x,y,\kappa )\;dx \end{array}$$

cannot be expressed in closed form. Instead, we maximize the evidence lower bound based on Kullback-Leibler divergence by utilizing an encoder *q*(***x*** ∣ ***y***, ***κ***):

$$\begin{array}{c} \;\text{log}\;p\left(y,\kappa \right)\geq E_{q(x|y,\kappa )}[\;\text{log}\;p(x)+\;\text{log}\;p(y|x)+\;\text{log}\;p(\kappa |x)-\;\text{log}\;q(x|y,\kappa )]. \end{array}$$ (4)

This encoder *q*(***x*** ∣ ***y***, ***κ***) should be easy to sample from and should allow for straightforward calculation of probabilities for generated samples. For this purpose, we adopt a structured variational family for this encoder [32, 34, 35]:

$$\begin{array}{c} q\left(x|y,\kappa \right)\propto q\left(x_{1}\right)\prod_{r=2}^{R}q\left(x_{r}|x_{r-1}\right)\prod_{r=1}^{R}q\left(x_{r}|y_{r}\right)\prod_{r=1}^{R}q\left(x_{r}|\kappa_{r}\right). \end{array}$$

Here, *q*(*x*_*r*_ ∣ *y*_*r*_) and *q*(*x*_*r*_ ∣ ***κ***_*r*_) are encoders that provide a probabilistic guess at each hidden state *x*_*r*_ inferred from observations *y*_*r*_ and ***κ***_*r*_. The distributions *q*(*x*_1_) and *q*(*x*_*r*_ ∣ *x*_*r*−1_) connect neighboring nodes in a Markov manner.

To achieve sampling efficiency, we define these densities as Gaussian distributions. First, we define the distributions *q*(*x*_1_) and *q*(*x*_*r*_ ∣ *x*_*r*−1_) as

$$\begin{array}{cc} q(x_{1}) & =N(x_{1}|b_{1},W_{1}),\\ q(x_{r}|x_{r-1}) & =N(x_{r}|Gx_{r-1}+b,W), \end{array}$$

where *b*_1_ is the initial mean, *W*_1_ is the initial precision matrix, *G* is the transition matrix, *b* is the transition offset, and *W* is the transition precision matrix.

The encoder *q*(*x*_*r*_ ∣ *y*_*r*_) approximates the likelihood of *x*_*r*_ given *y*_*r*_. We define this part as a Gaussian distribution with neural networks [34]:

$$\begin{array}{c} q(x_{r}|y_{r})=N(x_{r}|\nu_{\phi }^{y\to x}\left(y_{r}\right),\Xi_{\phi }^{y\to x}\left(y_{r}\right)), \end{array}$$

where $\nu_{\phi }^{y\to x}\left(y\right),\;\Xi_{\phi }^{y\to x}\left(y\right)$ are the mean vector and precision matrix expressed as neural networks, respectively.

The encoder *q*(*x*_*r*_ ∣ ***κ***_*r*_) approximates the point process likelihood. The point process likelihood is the product of *n*_*r*_ joint mark intensity terms λ(*x*_*r*_, *κ*_*ri*_) and the exponential term exp(−λ(*x*_*r*_)). To express this product property, we define *q*(*x*_*r*_ ∣ ***κ***_***r***_) as the product of *n*_*r*_ + 1 Gaussian experts with neural networks:

$$\begin{array}{c} q\left(x_{r}|\kappa_{r}\right)=(\prod_{i=1}^{n_{r}}N\left(x_{r}|\nu_{\phi }^{\kappa \to x}\left(\kappa_{ri}\right),\Xi_{\phi }^{\kappa \to x}\left(\kappa_{ri}\right)\right))\;N\left(x_{r}|\nu_{\phi }^{x},\Xi_{\phi }^{x}\right), \end{array}$$

where $\nu_{\phi }^{\kappa \to x}\left(\kappa \right),\;\Xi_{\phi }^{\kappa \to x}\left(\kappa \right)$ are the mean vector and precision matrix expressed as neural networks, and $\nu_{\phi }^{x},\;\Xi_{\phi }^{x}$ are also the mean vector and precision matrix. Here, $N(x_{r}|\nu_{\phi }^{\kappa \to x}\left(\kappa_{ri}\right),\Xi_{\phi }^{\kappa \to x}\left(\kappa_{ri}\right))$ approximates λ(*x*_*r*_, *κ*_*ri*_), and $N(x_{r}|\nu_{\phi }^{x},\Xi_{\phi }^{x})$ approximates exp(−λ(*x*_*r*_)).

The posterior distribution of ***x*** under these assumptions reduces to a Gaussian distribution whose precision matrix is a block tridiagonal matrix. By using the block tridiagonal structure, the computational cost of generating samples and calculating their densities is proportional to the number of bins *R* [34]. For more details of the sampling procedure, see Section S1.6 in S1 Text.

Once we get a sample ***x*** from the encoder *q*_*θ*_(***x*** ∣ ***y***, ***κ***), we then evaluate the inner expectation terms in the lower bound (4). While the prior *p*(*x*_1_), *p*(*x*_*r*_ ∣ *x*_*r*−1_) and the observation distribution for the covariate *p*(*y*_*r*_ ∣ *x*_*r*_) are straightforward to evaluate, the point process likelihood *p*(***κ***_*r*_ ∣ *x*_*r*_) is not analytically tractable. This term is replaced by its lower bound derived in Section 2.3:

$$\sum_{i=1}^{n_{r}}\frac{1}{\alpha }\;\text{log}\;(\frac{1}{L}\sum_{l=1}^{L}{\left(\frac{\lambda_{0}\;p\left(x_{r},\kappa_{ri}|z_{ril}\right)\;p\left(z_{ril}\right)}{q(z_{ril}|x_{r},\kappa_{ri})}\right)}^{\alpha })-{\left(\frac{1}{L}\sum_{l=1}^{L}{\left(\frac{\lambda_{0}\;p\left(x_{r}|z_{rl}\right)\;p\left(z_{rl}\right)}{q(z_{rl}|x_{r})}\right)}^{\beta }\right)}^{\frac{1}{\beta }},$$ (5)

where ${\left\{z_{ril}\right\}}_{l=1}^{L}$ and ${\left\{z_{rl}\right\}}_{l=1}^{L}$ are i.i.d. samples from *q*(*z* ∣ *x*_*r*_, *κ*_*ri*_) and *q*(*z* ∣ *x*_*r*_), respectively.

For training our model, we estimate all the parameters simultaneously by maximizing the lower bound (4). The Jackknife resampling and the doubly reparameterized gradients are also applicable in this maximization. For a summary of the parameter estimation, see Section S1.7 in S1 Text.

### 2.5 Decoding covariate from unsorted spikes

Next, we consider a decoding problem. Assume that only the unsorted spikes ***κ*** are observed and that we already know the model parameters. Under this assumption, the decoding aims to calculate the posterior of the hidden state ***x*** and the observed covariate ***y*** given the unsorted spikes ***κ***. Using Bayes’ rule and our state space model definition, this posterior is expressed as follows:

$$\begin{array}{cc} p(x,y|\kappa ) & =p(x|\kappa )\;p(y|x),\\ p(x|\kappa ) & =\frac{p(x,\kappa )}{\int p(x,\kappa )\;dx}, \end{array}$$

where *p*(***x***, ***κ***) = *p*(***x***)*p*(***κ*** ∣ ***x***) is the joint density of ***x*** and ***κ***. Since the posterior of ***y*** is determined by the posterior of ***x*** and the decoder *p*(***y*** ∣ ***x***), all we need to do is calculate the posterior *p*(***x*** ∣ ***κ***). Once we calculate *p*(***x*** ∣ ***κ***) and obtain samples ${\left\{x_{s}\right\}}_{s=1}^{S}$ from this posterior, the posterior of ***y*** can be approximated as

$$\begin{array}{c} p\left(y|\kappa \right)=\int p\left(y|x\right)\;p\left(x|\kappa \right)\;dx\approx \frac{1}{S}\sum_{s=1}^{S}p\left(y|x_{s}\right). \end{array}$$

The rest of this section focuses on calculating the posterior *p*(***x*** ∣ ***κ***). Under our model’s definition, the point process likelihood *p*(***κ*** ∣ ***x***) is not Gaussian, making the posterior *p*(***x*** ∣ ***κ***) analytically intractable. To tackle similar situations, previous studies have proposed a nonlinear filter algorithm for point process observations [16, 43, 44]. They recursively approximated the point process likelihood at each time step using the Laplace approximation and constructed a forward filtering algorithm. In this paper, we mainly use the direct optimization method described in [45, 46]. In this approach, we approximate the point process likelihood for all steps simultaneously and obtain the approximated posterior of ***x*** directly.

The Laplace approximation provides the approximated posterior by fitting a Gaussian distribution with a mean equal to the mode of the posterior and precision equal to the Hessian matrix of the negative log density evaluated at the mode. Denote the gradient and the Hessian matrix of log *p*(***x***, ***κ***) with respect to ***x*** as ∇_***x***_ log *p*(***x***, ***κ***) and $\nabla_{x}^{2}\;\text{log}\;p\left(x,\kappa \right)$, respectively. Then, the Laplace approximation for the posterior is given by

$$\begin{array}{cc} p(x|\kappa ) & \approx N(x|\hat{\mu },\hat{\Lambda }),\\ \hat{\mu } & =\underset{x}{\text{arg}\;\text{max}}\;\;\text{log}\;p\left(x,\kappa \right),\\ \hat{\Lambda } & =-\nabla_{x}^{2}\;\text{log}\;p\left(x,\kappa \right){|}_{x=\hat{\mu }}. \end{array}$$

Thus, approximating the posterior reduces to the optimization problem of finding the MAP solution $\hat{\mu }$ of the joint density.

Newton’s algorithm can be applied to find this $\hat{\mu }$. The Newton update rule at the *k*-th iteration is

$$\begin{array}{c} x^{\left(k+1\right)}=x^{\left(k\right)}-{\left(\nabla_{x}^{2}\;\text{log}\;p\left(x,\kappa \right){|}_{x=x^{\left(k\right)}}\right)}^{-1}\nabla_{x}\;\text{log}\;p\left(x,\kappa \right){|}_{x=x^{\left(k\right)}}. \end{array}$$ (6)

After *K* iterations of this update, starting from the initial value ***x***^(0)^, the mean and precision matrix of the Laplace approximation can be determined as

$$\begin{array}{c} \hat{\mu }=x^{\left(K\right)},\;\hat{\Lambda }=-\nabla_{x}^{2}\;\text{log}\;p\left(x,\kappa \right){|}_{x=x^{\left(K\right)}}. \end{array}$$

However, a challenge arises due to the dimensionality of ***x***. In our problem, the length of ***x*** is proportional to the number of bins, *R*. This makes multiplying the inverse Hessian $\nabla_{x}^{2}\;\text{log}\;p\left(x,\kappa \right)$ with the gradient ∇_***x***_ log *p*(***x***, ***κ***) computationally expensive. This issue can be addressed by using the block tridiagonal structure of the Hessian matrix [45].

A critical issue specific to our model is that the joint density log *p*(***x***, ***κ***) is not always guaranteed to be concave. This leads to two problems. The first problem is that the Newton update does not always increase the joint density, leading to convergence to poor solutions. The second problem is that the precision matrix $\hat{\Lambda }$ may not be positive definite, making the approximate Gaussian distribution invalid.

To address these issues, we approximate the log joint density as a concave function at each iteration. The log joint density is the summation of the log prior and the point process log-likelihood as

$$\begin{array}{cc} \;\text{log}\;p(x,\kappa ) & =\;\text{log}\;p(x)+\;\text{log}\;p(\kappa |x)\\ & =\;\text{log}\;p\left(x\right)+\sum_{r=1}^{R}\;\text{log}\;p\left(\kappa_{r}|x_{r}\right). \end{array}$$

Since the point process log-likelihood is not analytically tractable under our model, this term is replaced by the lower bound (5). As the log prior follows a Gaussian distribution, the first term is already concave. Consequently, our primary task is to approximate the latter lower bound term to be concave.

Hereafter, let us consider the concave approximation of the lower bound around the point $\bar{x}={\left\{{\bar{x}}_{r}\right\}}_{r=1}^{R}$. To ensure the concavity, we employ the technique used in Gauss-Newton optimization [47]. This optimization is applied to the objective function that can be represented as a composite of a smooth map and a concave function.

To apply this technique, we first rewrite the Rényi lower bound and the *χ* upper bound as composite forms:

$$\begin{array}{cc} \pi_{F_{ri}}\left(x_{r}\right) & ≔\pi (F_{ri}\left(x_{r}\right))=\frac{1}{\alpha }\;\text{log}\;(\frac{1}{L}\sum_{l=1}^{L}{\left(\frac{\lambda_{0}\;p\left(x_{r},\kappa_{ri}|z_{ril}\right)\;p\left(z_{ril}\right)}{q(z_{ril}|{\bar{x}}_{r},\kappa_{ri})}\right)}^{\alpha }),\\ \rho_{G_{r}}\left(x_{r}\right) & ≔\rho (G_{r}\left(x_{r}\right))=-{\left(\frac{1}{L}\sum_{l=1}^{L}{\left(\frac{\lambda_{0}\;p\left(x_{r}|z_{rl}\right)\;p\left(z_{rl}\right)}{q(z_{rl}|{\bar{x}}_{r})}\right)}^{\beta }\right)}^{\frac{1}{\beta }}, \end{array}$$

where $F_{ri},\;G_{r}:X\to R^{L}$ are smooth maps, $\pi ,\;\rho :R^{L}\to R$ are concave functions, and $\pi_{F_{ri}},\;\rho_{G_{r}}$ are composite functions. A detailed definition of these maps and functions is provided in Section S1.8 in S1 Text. With these notations, the lower bound of the point process log-likelihood at the *r*-th bin is

$$\begin{array}{c} L_{r}\left(x_{r}\right)≔\sum_{i=1}^{n_{r}}\pi_{F_{ri}}\left(x_{r}\right)+\rho_{G_{r}}\left(x_{r}\right). \end{array}$$

Now, we are ready to apply the Gauss-Newton optimization. To obtain the concave approximation of this lower bound, we approximate *F*_*ri*_(*x*_*r*_), *G*_*r*_(*x*_*r*_) by a first-order Taylor expansion around ${\bar{x}}_{r}$, and use them as inputs to *π* and *ρ*:

$$\begin{array}{cc} \hat{\pi_{F_{ri}}}\left(x_{r}\right) & ≔\pi (F_{ri}\left({\bar{x}}_{r}\right)+J_{F_{ri}}\left({\bar{x}}_{r}\right)\left(x_{r}-{\bar{x}}_{r}\right)),\\ \hat{\rho_{G_{r}}}\left(x_{r}\right) & ≔\rho (G_{r}\left({\bar{x}}_{r}\right)+J_{G_{r}}\left({\bar{x}}_{r}\right)\left(x_{r}-{\bar{x}}_{r}\right)), \end{array}$$

where $J_{F_{ri}}\left({\bar{x}}_{r}\right)$ and $J_{G_{r}}\left({\bar{x}}_{r}\right)$ are the Jacobian matrices of *F*_*ri*_ and *G*_*r*_ at ${\bar{x}}_{r}$, respectively. Since *π* and *ρ* are concave functions and their inputs are linear in terms of *x*, both $\hat{\pi_{F_{ri}}}$ and $\hat{\rho_{G_{r}}}$ are concave functions. With these approximations, the approximated lower bound

$$\begin{array}{c} {\hat{L}}_{r}\left(x_{r}\right)≔\sum_{i=1}^{n_{r}}\hat{\pi_{F_{ri}}}\left(x_{r}\right)+\hat{\rho_{G_{r}}}\left(x_{r}\right) \end{array}$$

is always concave. Consequently, the log joint density replaced with this approximated lower bound

$$\begin{array}{c} \;\text{log}\;p\left(x\right)+\sum_{r=1}^{R}{\hat{L}}_{r}\left(x_{r}\right) \end{array}$$

is also concave.

This established concavity addresses two prior concerns. First, the concavity ensures that the Newton step consistently moves in an ascending direction throughout optimization. Second, the precision matrix $\hat{\Lambda }$ derived from the Laplace approximation is guaranteed to be positive definite, ensuring the validity of the resulting Gaussian distribution.

We substitute this approximated objective into the Newton update rule (6). Owing to the block tridiagonal structure of the Hessian matrix, the computational cost for each Newton step is directly proportional only to the number of bins *R* [45]. For detailed information, see Section S1.8 in S1 Text.

## 3 Results

In this section, we apply our model and other models to synthetic data and place cell spiking activities. In Section 3.1, we explain the metrics used in the experiments. In Section 3.2, we describe the models being compared. In Section 3.3 and Section 3.4, we present descriptions of the datasets and the results of the experiments. In Section 3.5, we conduct comparisons between models and highlight the strengths of our model.

We implemented our model in TensorFlow. The implementation of our model is available at https://github.com/nttcslab/mppvae.

### 3.1 Tasks and metrics

The prediction task aims to evaluate the model’s ability to predict the future covariate and unsorted spikes. In this task, we calculated the negative log-likelihood (NLL) of the test data:

$$\begin{array}{c} \text{NLL}=-\;\text{log}\;p(y,\kappa ). \end{array}$$

This metric shows how well the model predicts the covariate and unsorted spikes in a future interval. A smaller NLL indicates better predictive performance. Our model defines the joint mark intensity as the integral of the decoder, making the point process log-likelihood not analytically tractable. For such types of models, we use the lower bound of the log-likelihood instead to calculate NLL. Since this value becomes larger than the true NLL, our model is at a disadvantage in the comparison. Thus, if our model achieves superior performance compared to other models, the gap from the true NLL does not change the relative ranking of the models.

In the decoding task, we reconstructed the observed covariate $\hat{y}$ from the unsorted spikes ***κ***. Then, we calculated the mean squared error (MSE) between the reconstructed covariate and the true covariate as

$$\begin{array}{c} \text{MSE}=\frac{1}{R}\sum_{r=1}^{R}{\left\|{\hat{y}}_{r}-y_{r}\right\|}_{2}^{2}, \end{array}$$

where ‖⋅‖_2_ is the *l*_2_ norm. A smaller MSE indicates better decoding performance.

Fig 2 summarizes the observed variables in the training step and evaluation tasks.

### 3.2 Compared models

To demonstrate the effectiveness of our model, we compared it with other marked point process models. In this section, we provide a brief overview of three of these models. These models define the joint mark intensity in different ways while sharing the same state space model described in Section 2.4. For details on definitions, hyperparameters, parameter estimation, and decoding procedures, see Section S2 in S1 Text.

We did not apply the KDE-based model [9] due to memory issues, which scale linearly with the number of spikes and the length of waveforms (e.g., 170,000 × 256 for Gatsby_08282013).

For notational simplicity, we denote our model as JVAE in the figures, short for the joint mark intensity model using VAE.

#### CVAE

We construct another joint mark intensity model based on a conditional variational autoencoder, denoted as CVAE. The conditional variational autoencoder is an extension of the variational autoencoder used for conditional density estimation [48, 49]. To use this model, we decompose the joint mark intensity into the product of two functions:

$$\begin{array}{c} \lambda (x,\kappa )=\lambda (x)\;p(\kappa |x). \end{array}$$

Here, λ(*x*) is called the ground intensity, and *p*(*κ* ∣ *x*) is the mark density given the state. Based on this decomposition, the point process log-likelihood is expressed as the summation of two parts:

$$\begin{array}{c} \sum_{r=1}^{R}\sum_{i=1}^{n_{r}}\;\text{log}\;p\left(\kappa_{ri}|x_{r}\right)+\sum_{r=1}^{R}[n_{r}\;\text{log}\;\lambda \left(x_{r}\right)-\lambda \left(x_{r}\right)]. \end{array}$$ (7)

The first part is the conditional log-likelihood that explains the dependency of the marks on the states. The second part is the non-marked point process log-likelihood. Thus, the parameter estimation reduces to two separate estimations by maximizing the corresponding terms.

In this model, we use a deterministic neural network for the ground intensity λ(*x*) and the conditional variational autoencoder for the mark density *p*(*κ* ∣ *x*). Specifically, we define the mark density as

$$\begin{array}{c} p(\kappa |x)=\int p(\kappa |z)\;p(z|x)\;dz, \end{array}$$

where *p*(*κ* ∣ *z*) is a decoder for mark *κ* and *p*(*z* ∣ *x*) is a prior for *z* dependent on *x*. Similar to our model, the likelihood of this model is not analytically tractable. Hence, we introduce the encoder *q*(*z* ∣ *x*, *κ*) to derive the lower bound and estimate parameters of the decoder, encoder, and prior by maximizing this lower bound.

For the decoder, encoder, and prior, we use Gaussian distributions whose means and precision matrices are neural networks:

$$\begin{array}{c} p\left(\kappa |x,z\right)=N\left(\kappa |\mu_{\theta }^{\text{xz}\to \kappa }\left(x,z\right),\Lambda_{\theta }^{\text{xz}\to \kappa }\left(x,z\right)\right),\;p\left(z|x\right)=N\left(z|\mu_{\theta }^{x\to z}\left(x\right),\Lambda_{\theta }^{x\to z}\left(x\right)\right),\;\\ q\left(z|x,\kappa \right)=N(z|\nu_{\phi }^{x\kappa \to z}\left(x,\kappa \right),\Xi_{\phi }^{x\kappa \to z}\left(x,\kappa \right)). \end{array}$$

#### GMM

The second model for comparison is a Gaussian mixture model multiplied by a scale parameter, denoted as GMM [12–15]. In this model, the joint mark intensity is defined as

$$\begin{array}{c} \lambda \left(x,\kappa \right)=\sum_{l=1}^{L}\lambda_{l}\;N\left(x|\mu_{l}^{x},\Lambda_{l}^{x}\right)\;N\left(\kappa |\mu_{l}^{\kappa },\Lambda_{l}^{\kappa }\right), \end{array}$$

where λ_*l*_ is a scale parameter for the *l*-th component, $\mu_{l}^{x}$ and $\Lambda_{l}^{x}$ are the mean vector and precision matrix for the ground intensity, and $\mu_{l}^{\kappa }$ and $\Lambda_{l}^{\kappa }$ are the mean vector and precision matrix for the mark distribution.

#### RMPP

The last model for comparison is based on a recurrent marked point process [50], denoted as RMPP. This model assumes that event generation at time *t* depends on the history of the previous events ${\{(t_{i},\kappa_{i})\text{\}}}_{t_{i}<t}$. To express such dependency, it introduces a latent state that determines the event generation around time *t*, and models the evolution of this state with a recurrent neural network. Based on this idea, we assume that only the mark distribution is determined by the history of the process:

$$\begin{array}{c} \lambda \left(t,x,\kappa |{\left\{\left(t_{i},x_{i},\kappa_{i}\right)\right\}}_{t_{i}<t}\right)=\lambda \left(x\right)\;p\left(\kappa |{\left\{\left(t_{i},x_{i},\kappa_{i}\right)\right\}}_{t_{i}<t}\right). \end{array}$$

Let *z*_*i*_ be a latent variable assigned to a pair consisting of a state value *x*_*i*_ and an unsorted spike (*t*_*i*_, *κ*_*i*_). Assume that the latent state dynamics are governed by a Gated Recurrent Unit [51]:

$$\begin{array}{c} z_{i}=\text{GRU}\left(z_{i-1},\Delta t_{i-1},x_{i-1},\kappa_{i-1}\right), \end{array}$$

where GRU(⋅) is a forward step of a gated recurrent unit and Δ*t*_*i*_ = *t*_*i*_ − *t*_*i*−1_ is the inter-event interval. Given these latent states, the mark distribution at the i*-th spike time *t*_i*_ is defined as a Gaussian mixture whose parameters are neural networks:

$$\begin{array}{c} p\left(\kappa |{\left\{\left(t_{i},x_{i},\kappa_{i}\right)\right\}}_{t_{i}<t_{i^{*}}}\right)=\sum_{l=1}^{L}\pi_{l}\left(z_{i^{*}}\right)\;N\left(\kappa |\mu_{l}^{z\to \kappa }\left(z_{i^{*}}\right),\Lambda_{l}^{z\to \kappa }\left(z_{i^{*}}\right)\right), \end{array}$$ (55)

where *π*_*l*_(*z*) is a mixture weight, and $\mu_{l}^{z\to \kappa }\left(z\right)$ and $\Lambda_{l}^{z\to \kappa }\left(z\right)$ are the mean vector and precision matrix for the *l*-th component, expressed as neural networks.

This model explicitly handles the historical dependency of the unsorted spikes, and such a definition contradicts a key assumption of the state-space model in Section 2.4. To use the state-space model, the observed covariate ***y*** and the unsorted spikes ***κ*** should be independent given the hidden state ***x***. Thus, the decoding method explained in this paper is not applicable to RMPP. Furthermore, since the memory usage increases linearly with the number of spikes, RMPP cannot be applied to the place cell data (*n* ≈ 100, 000). For these reasons, we applied RMPP only to the prediction task using the synthetic data.

#### GRU, DNN, WF

To compare the performance of our model with sorting-based decoding methods, we also applied the decoders implemented in [52] to the datasets. This implementation contains several regression methods that receive the binned spikes and output the predicted covariate.

We applied three regression decoders, named GRU, DNN, and WF. GRU uses a gated recurrent unit, DNN uses a feedforward neural network, and WF employs a Wiener filter, which corresponds to linear regression.

### 3.3 Synthetic data

#### Data generation

We generated datasets consisting of covariate time series and unsorted spikes influenced by this covariate. In line with our state space definition, we assumed that a hidden state exists and influences both the covariate and unsorted spikes. To demonstrate our model’s ability to handle nonlinear dynamics, we employed the Lorenz attractor as this hidden state. The Lorenz attractor is a chaotic solution to the nonlinear differential equation:

$$\begin{array}{c} \frac{d}{dt}[\begin{array}{c} x_{1t}\\ x_{2t}\\ x_{3t} \end{array}]=[\begin{array}{c} \alpha (x_{2t}-x_{1t}),\\ x_{1t}\;\left(\beta -x_{3t}\right)-x_{2t},\\ x_{1t}\;x_{2t}-\gamma x_{3t} \end{array}], \end{array}$$

where *α*, *β*, and *γ* are parameters.

Given the hidden state, the two types of observed covariates were defined as follows:

(i) The observed covariate equals the hidden state,
  $$\begin{array}{c} y_{t}=[\begin{array}{c} x_{1t}\\ x_{2t}\\ x_{3t} \end{array}]. \end{array}$$ (8)
  The data with this covariate was referred to as Lorenz_3D.
(ii) The observed covariate was a two-dimensional projection of the hidden state,
  $$\begin{array}{c} y_{t}=[\begin{array}{c} x_{1t}\\ x_{3t} \end{array}]. \end{array}$$ (9)
  The data with this covariate was referred to as Lorenz_2D. In this case, we could not access information for *x*_2*t*_, which also had an influence on the generation of unsorted spikes.

We assumed that there were four probes measuring different neural population activities and that unsorted spikes were generated from the marked inhomogeneous Poisson process that depends on the hidden state at each probe. To imitate the activities of multiple neurons, the joint mark intensity of this process was defined as a mixture representation:

$$\sum_{l=1}^{L}\lambda_{l}\;\text{exp}\;({\left(\frac{t}{T}\;c_{l}+(1-\frac{t}{T})\;d_{l}\right)}^{⊤}x)\;\int N\left(\kappa |\mu^{z\to \kappa }\left(z\right),\Lambda^{z\to \kappa }\left(z\right)\right)\;N\left(z|\mu_{l}^{z},\Lambda_{l}^{z}\right)\;dz.$$ (10)

Here, λ_*l*_ is a scale parameter of the *l*-th component, and $c_{l},\;d_{l}\in R^{3}$ are the most favorable directions at *t* = 0 and *t* = *T*, respectively. The intensity of the *l*-th component reaches its maximum value when *x*_*t*_ aligns with the most favorable direction determined by these vectors. In this definition, this direction gradually changes from *c*_*l*_ to *d*_*l*_, which expresses the plasticity of spiking activities. The mark of the *l*-th component is distributed according to a Gaussian decoder N(*κ* ∣ *μ*^z→κ^(*z*), Λ^z→κ^(*z*)) with a Gaussian prior whose mean vector and precision matrix are $\mu_{l}^{z}$ and $\Lambda_{l}^{z}$, respectively. We selected the parameters of this decoder and prior so that the generated marks resemble the actual extracellular waveforms with 32 time steps in [53].

**Fig 3:**
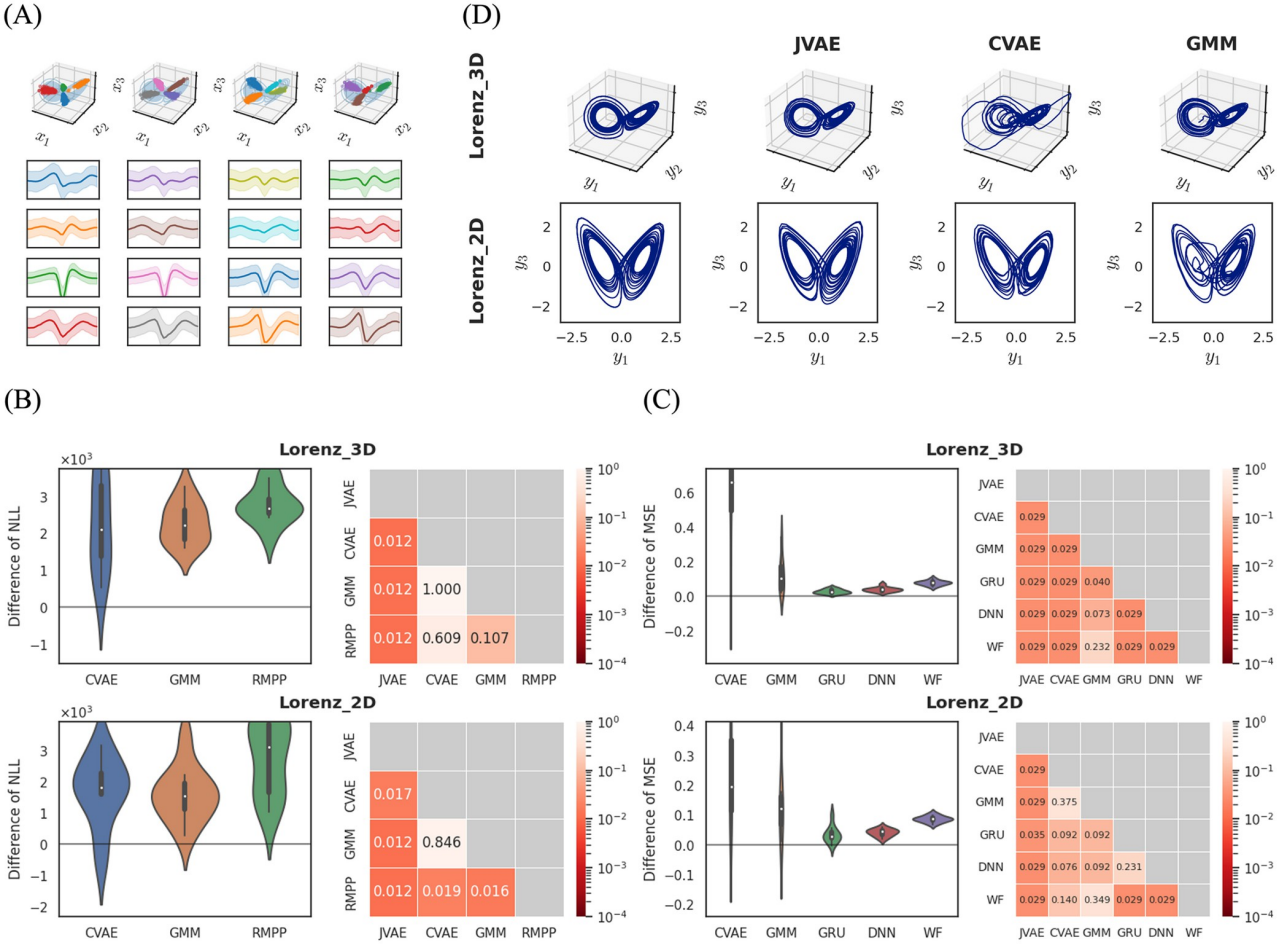
(A) Synthetic data. We assumed that the dataset consisted of four sets of unsorted spikes measured at different probes. Each set of unsorted spikes consists of four components that are tuned to different state values and generate spikes with varied waveforms. Each column indicates the spike times and waveforms measured at each probe. (1st row) The hidden state and spike times: A blue line represents the hidden state *x*_*t*_. Colored dots on this line denote the state values at spike times for each component. (2nd to 5th row) The waveform distribution of each component. The solid line and colored band show the mean and two-sigma interval of the waveform distribution. (B) The prediction performance for synthetic data. The first row shows the results from Lorenz_3D and the second shows the results from Lorenz_2D. (Left) Violin plots of NLL. These values are subtracted by the baseline NLL of JVAE (our model). The white dots represent the median, the thick black bars indicate the interquartile range, and the thin black bars show the range from the minimum to the maximum values. (Right) The *p* values in the Wilcoxon test for assessing differences between two models. Each cell indicates the result for a pairing of two models. These *p* values are corrected by the Holm-Sidak method. The color indicates levels of the *p* values. (C) The decoding performance for synthetic data. (Left) Violin plots of MSE. (Right) The *p* values in the Wilcoxon test for assessing differences between two models. (D) Decoding results for the synthetic data. The first row shows the results from Lorenz_3D and the second shows the results from Lorenz_2D. (1st column) The true observed covariate. (2nd to 4th columns) Reconstructed covariates.

Under this definition, we generated ten trials of data. We first calculated ten paths of the Lorenz attractor for a duration of *T* = 100, each with different initial values for *x*_*t*_ at *t* = 0. Using these paths, we defined the observed covariates *y*_*t*_ by (8) or (9), and generated the unsorted spikes {(*t*_*i*_, *κ*_*i*_)} by (10). To use our state space representation, we split the time axis into bins with a length of Δ*t* = 0.01 and prepared the discrete observations ***y*** and ***κ***. Fig 3A shows the data generated by this procedure.

#### Model training and evaluation

We divided the data into three parts along the time axis: 80% for training, 10% for validation, and 10% for testing. Using the training data, we estimated the state space model *p*(***x***, ***y***, ***κ***), including the joint mark intensity λ(*x*, *κ*). As mentioned in Section 2.4, we did not have access to information about the hidden state, and the dimension of *x*_*r*_ is an unknown hyperparameter. Thus, we defined the hidden state *x*_*r*_ as a ten-dimensional vector.

For training the models, we used the Adam optimizer to maximize the objectives and used the validation data for early stopping to avoid overfitting. With the obtained model, NLL and MSE scores were calculated using the test data. We repeated this procedure ten times in different trials. Then, given the calculated scores, we conducted the Wilcoxon signed-rank test to assess the statistically significant difference between every pair of models. Since these were multiple comparisons, we modified the *p* values using the Holm-Sidak method. We concluded that there was a significant difference if the *p*-value was < 0.05.

#### Task results

In the prediction task, our model consistently outperformed the other marked point process models in all trials (Fig 3B). For Lorenz_3D, there were no significant performance differences among the other three models. In contrast, for Lorenz_2D, CVAE and GMM performed better than RMPP.

In the decoding task, our model achieved the best performance (Fig 3C). For Lorenz_3D, GMM performed better than CVAE. When compared to sorted decoding, both CVAE and GMM resulted in significantly worse performance. Among the sorted decoding methods, GRU outperformed DNN and WF in both datasets.

Our model succeeded in reconstructing the true attractors for both datasets (Fig 3D). While CVAE and GMM also reconstructed the attractors roughly, their reconstructed paths sometimes deviated from the true paths, leading to an increase in the MSE scores. In contrast, although the paths reconstructed by sorted decoding were noisy, they did not deviate significantly from the true paths (see Fig B in S1 Text).

### 3.4 Unsorted place cell spiking activities

#### Data description

We applied our model to place cell multi-unit spiking activities in the hippocampus of rats freely foraging on various tracks [53, 54]. A place cell has a receptive field known as a “place field” and emits spikes when a rat passes through its place field [55]. Estimating the firing rate of each place cell as a function of spatial coordinates provides insight into how a neuron population codes spatial information. The spiking activities of place cells also offer an excellent example of neural decoding. When using place cell data for neural decoding, the goal is to reconstruct the rat’s trajectories from the spikes. The success of decoding serves as evidence that the place cell population activity contains sufficient information to recover the rat’s trajectory.

Let us summarize the correspondence between mathematical variables in our model and the components of this data. The rat trajectory is the observed covariate *y*_*t*_, and the sequence of spikes attached with their waveforms on the probes is the unsorted spikes {(*t*_*i*_, *κ*_*i*_)}. The state space model *p*(***x***, ***y***, ***κ***) explains the hidden dynamics behind these data and how this hidden state affects the rat trajectories and its place cell spiking activities. The joint mark intensity λ(*x*, *κ*) describes the firing rate of spikes with the waveform *κ* when the rat’s hidden state is *x*. The decoding task aims to reconstruct the rat trajectory ***y*** from the unsorted spikes ***κ***.

**Fig 4:**
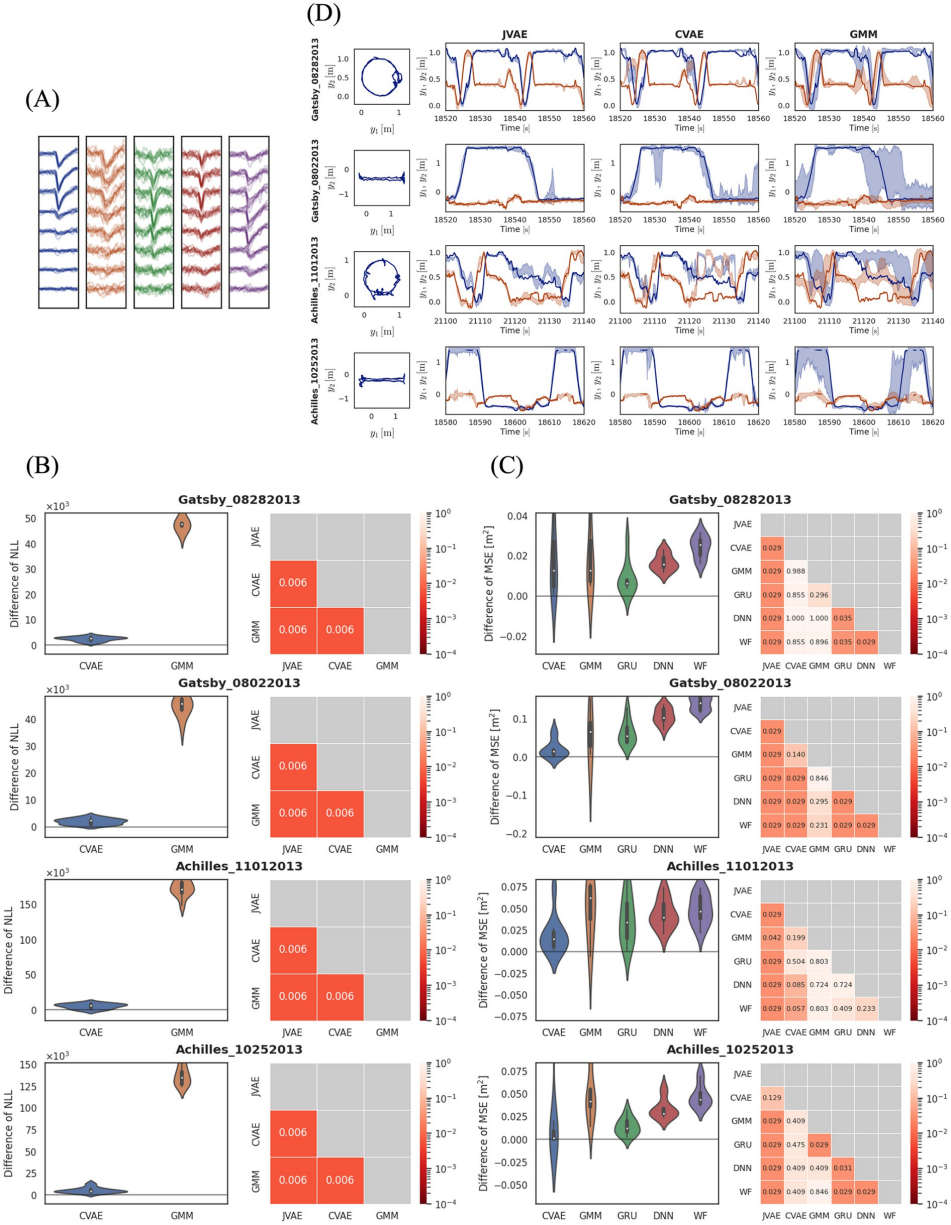
(A) Waveforms of spikes. Each panel shows the collection of spike waveforms classified into the same cluster. (B) Prediction performance for the rat place cell spiking activities. Each row shows the result of each trial. (Left) Violin plots of NLL. These values are subtracted by the baseline NLL of JVAE (our model). The white dots represent the median, the thick black bars indicate the interquartile range, and the thin black bars show the range from the minimum to the maximum values. (Right) The *p* values in the Wilcoxon test for assessing differences between two models. Each cell indicates the result for a pairing of two models. These *p* values are corrected by the Holm-Sidak method. The color indicates levels of the *p* values. (C) Decoding performance for the rat place cell spiking activities. (Left) Violin plots of MSE. (Right) The *p* values in the Wilcoxon test for assessing differences between two models. (D) Decoding results for the rat place cell spiking activities. Each row shows the result of each trial. (1st column) 2D plots of the rat trajectories in the circular or linear track. (2nd to 4th columns) True and reconstructed rat trajectories. Solid blue and red lines show the coordinates *y*_1_ and *y*_2_ of the actual rat trajectories during the experiment. Intervals without solid lines indicate that the rat trajectories were missing during those intervals due to device issues. The blue and red bands represent the two-sigma predictive intervals.

We selected four trials from the dataset: two different rats foraging on a linear track and a circular track [53, 54]. We refer to these trials by their respective directory names in the dataset: Gatsby_08282013, Gatsby_08022013, Achilles_11012013, and Achilles_10252013. In those trials, probes with four, eight, or ten recording sites were inserted into the rat hippocampus, and electrical potentials were measured simultaneously at each recording site. Spikes were detected using threshold processing, and spike waveforms with 32 time steps were extracted from the electrical potential signals at each site. The waveforms of all sites were concatenated into one vector and used as a spike feature *κ*. One trial contains around 200,000 spikes in a 400-second interval. Fig 4A shows the spike waveforms of clusters assigned by spike sorting.

We concatenated the spike waveforms measured at multiple sites into one vector and used this as a mark *κ*. This *κ* is a 320-dimensional vector at one of the probes. The observed covariate *y*_*t*_ is a two-dimensional spatial coordinate. To use our state space representation, we split the time axis into bins with length Δ*t* = 0.025 and prepared the discrete observations ***y*** and ***κ***.

#### Model training and evaluation

We split the data into ten partitions and used eight for training, one for validation, and one for testing.

The training and evaluation procedure was the same as that described in Section 3.3. We repeated the training and evaluation process ten times, changing the partitions each time.

#### Task results

In the prediction task, our model outperformed both CVAE and GMM across all trials (Fig 4B). In contrast to the results for synthetic data, CVAE also performed significantly better than GMM.

In the decoding task, our model exhibited better performance in three trials (Fig 4C). CVAE exhibited better performance in Gatsby_08022013 than GMM but did not show significant improvement across all trials. Unlike the results for synthetic data, GMM had poor decoding performance. When compared to sorted decoding, CVAE performed better, particularly in Gatsby_08022013.

Our model successfully recovered the nonlinear trajectories of rats from unsorted spikes (Fig 4D). While CVAE generally reconstructed the rat trajectories well, it occasionally deviated significantly from the true paths (e.g., interval [21120, 21130] in Achilles_11012013). GMM provided a rough approximation of the rat trajectories but failed to accurately capture the true paths. The trajectories reconstructed by sorted decoding were noisy and exhibited abrupt jumps (see Fig C in S1 Text).

### 3.5 Comparison

In this section, we first discuss three issues with the compared models used in the experiments. Next, we explain how our model overcomes these issues.

The first issue is the overfitting of the ground intensity. For synthetic data, CVAE demonstrated performance comparable to GMM in the prediction task but failed in the decoding task. The primary cause of the large MSE values for CVAE was the deviation of the reconstructed path from the true path. This deviation might be attributable to overfitting during the training phase. CVAE and RMPP utilize a neural network to define the ground intensity λ(*x*), adjusting this network to maximize the log-likelihood (7). Since the log-likelihood contains the term −λ(*x*), this network tends to assign zero mass to points where no spikes are observed. In fact, the optimal solution that maximizes the log-likelihood is given by

$$\begin{array}{c} \lambda \left(x\right)=\sum_{r=1}^{R}n_{r}\delta_{x_{r}}\left(x\right), \end{array}$$

where $\delta_{x_{r}}\left(x\right)$ is a Dirac delta function equal to 1 when *x* = *x*_*r*_. Thus, if the neural network comprises too many layers and units, it fits the training data too well, thereby failing to generalize to the test data. This overfitting negatively impacts the decoding performance. During decoding, we approximate the log-likelihood with a concave function. If the network for the ground intensity overfits to the training data and approaches the optimal solution mentioned above, its concave approximation significantly deviates from the true ground intensity. Such an incorrect approximation might result in a large deviation from the true path. Therefore, addressing overfitting is crucial to achieving better decoding results.

The second issue is the deviation of simple parametric models from the true joint mark intensity, which may occur in GMM and RMPP. This deviation might have been caused by two factors: nonlinear embeddings and complex waveform distributions. Both GMM and RMPP showed poorer performance for Lorenz_2D than our model. Our state space model attempts to linearize the Lorenz attractor dynamics in the hidden state space and recover missing information *x*_2*t*_ through its nonlinear embedding. This embedding determines the hidden state space and, consequently, the joint mark intensity within this space. However, this embedding does not guarantee that the joint mark density will be a mixture of unimodal functions. GMM and RMPP, which assume a Gaussian mixture for the joint mark intensity, fail to capture the complicated shape of the joint mark intensity induced by the embedding. For the place cell data, GMM’s prediction performance was worse than that of CVAE. The spike waveform in the place cell dataset was a high-dimensional vector, and its distribution was more complex than that of the synthetic data. Therefore, simple parametric models like Gaussian mixtures are insufficient to represent actual waveforms.

The third issue, specific to sorted decoding, is the discontinuity of the observed spikes. For synthetic data, GRU demonstrated performance comparable to both CVAE and GMM. However, as shown in Fig C in S1 Text, the reconstructed paths were noisier compared to unsorted decoding and exhibited abrupt jumps. The primary cause of these jumps is that the paths reconstructed through sorted decoding reflect the discontinuity observed in the spikes. The regression-based decoders do not impose any assumptions on the continuity of the hidden states. Thus, the similarity between reconstructed covariate values across consecutive bins was derived from the similarities between spike count vectors across consecutive bins. When spikes occur, these vectors exhibit abrupt jumps, causing discontinuity in the inputs of the decoders and resulting in noisy reconstructed covariate values. For place cell data, the performance of sorted decoding was inferior to CVAE in one trial. This might have been due to the discontinuity induced by unreliable spike assignment. Neural population activities often include a hash cluster, which is poorly isolated from other clusters [18, 56]. Spike sorting assigns labels to spikes within this cluster by using hard decision boundaries, overlooking the uncertainty of these spikes. Such labeling causes discontinuity in the input vectors, leading to noisy reconstructed paths.

Our model addresses these issues. First, the use of a variational autoencoder avoids overfitting. Our model maps a low-dimensional latent variable *z* to the joint representation of the hidden state *x* and the mark *κ*, and imposes a prior over *z*. This model definition acts as regularization. Second, by utilizing neural networks, our model can capture the complex interdependencies between hidden states and marks in a data-driven manner. This works effectively even when the nonlinear embedding distorts the true joint mark intensity. Third, our model defines joint mark intensity to represent unsorted spikes, thus avoiding the discontinuities introduced by the assignment of unclusterable spikes. The information of spikes with ambiguous waveforms has a small influence on decoding. Moreover, our state space model defines the hidden state dynamics, enabling us to obtain smoother reconstructed paths.

### 3.6 Application to Neuropixels data

To demonstrate the applicability of our model to data collected with high-density probes, we applied it to the open dataset [57]. This dataset includes electrophysiological recordings from multiple brain regions of mice using Neuropixels probes and behavioral data during decision-making tasks. For more details on the dataset, see [57].

**Fig 5:**
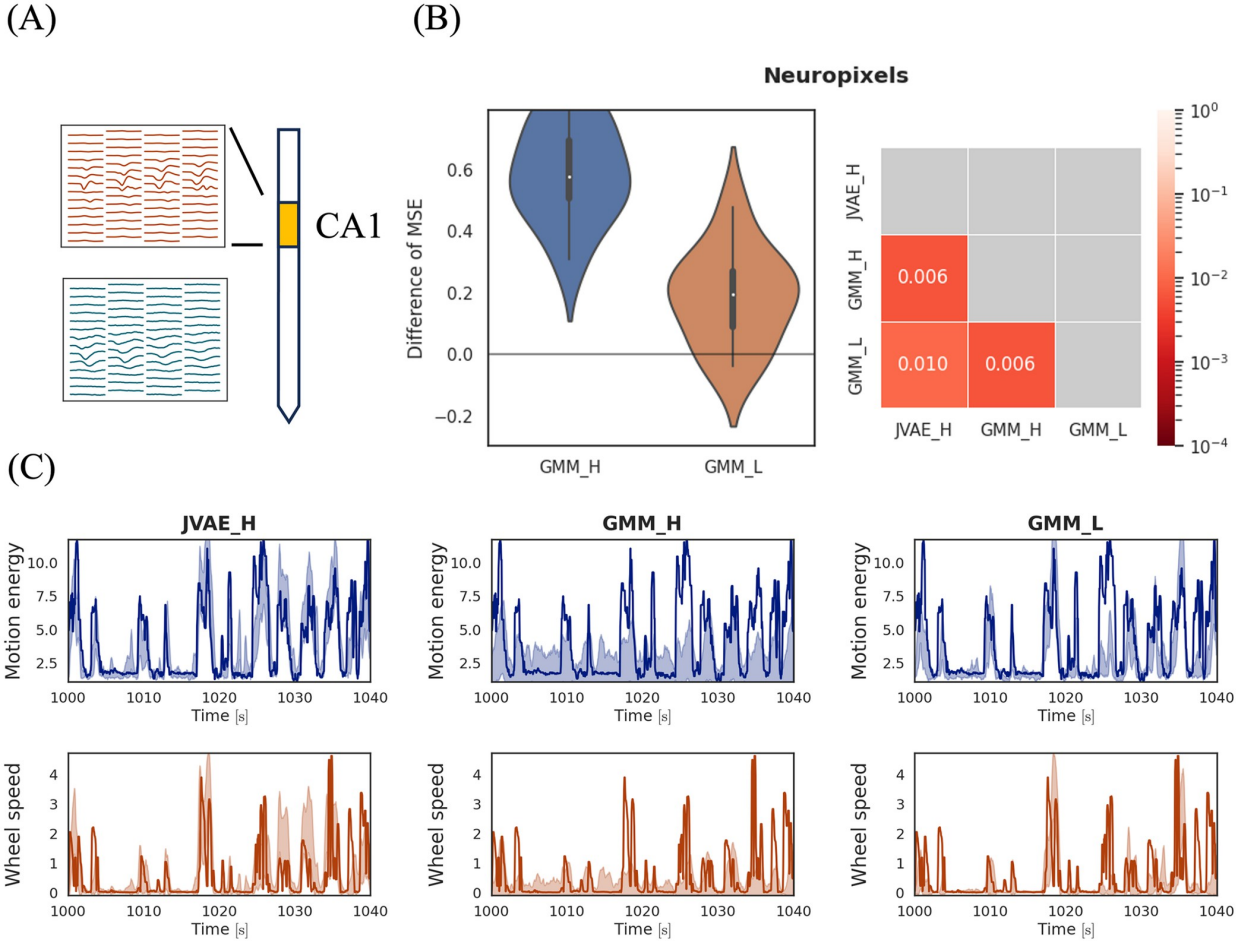
(A) Waveforms of spikes. Spike waveforms measured at 56 sites in the CA1 region were used for the spike feature. (B) Decoding performance for the Neuropixels data. (Left) Violin plots of MSE. These values are subtracted from the baseline MSE of JVAE_H (our model). The white dots represent the median, the thick black bars indicate the interquartile range, and the thin black bars show the range from the minimum to the maximum values. (Right) The *p* values in the Wilcoxon test for assessing differences between two models. Each cell indicates the results for a pairing of two models. These *p* values are corrected by the Holm-Sidak method. The color indicates levels of the *p* values. (C) Decoding result for the Neuropixels data. The first row shows the result of the motion energy, and the second shows the wheel speed. Solid blue and red lines show the actual values during the experiment. The blue and red bands represent the one-sigma predictive intervals.

The Neuropixels probe has 384 sites and measures a wide range of neuron populations. We used recordings from 56 sites located in the CA1 region. This dataset also includes spike sorting results provided by Kilosort [58], consisting of spike times, spike amplitudes, and spike depths on the probe. Using the times of spikes with estimated depths in the CA1 region, we extracted the waveforms for 32 time steps around each spike from the raw recording (Fig 5A). Then, we concatenated these values into one vector and used it as a spike feature *κ*. The dimension of *κ* was 1792. The mouse’s face motion energy and wheel speed were used as covariates. The interval used for this experiment was 400 seconds long and included approximately 150,000 observed spikes.

We applied our model (denoted as JVAE_H) and the Gaussian mixture model (GMM_H) to this dataset. Additionally, we applied the Gaussian mixture model to the data with a two-dimensional spike feature, including spike amplitudes and spike depths on the probe (GMM_L).

Fig 5B and 5C show the decoding results. JVAE_H outperformed both GMM_H and GMM_L, indicating that our model effectively handles high-dimensional spike features and is useful for Neuropixels data. GMM_L performed better than GMM_H, consistent with the comparison of decoding using low-dimensional and high-dimensional spike features presented in Section S4 in S1 Text.

## 4 Discussion

In this paper, we proposed a new joint mark intensity model. Our model decomposes the joint mark intensity into a nonnegative scale parameter and a probability density function, expressing the latter density as a variational autoencoder consisting of neural networks. With this model, we derived the lower bound for the point process log-likelihood by merging the evidence lower bound and upper bound. This lower bound provides stable gradient estimates during parameter estimation. Furthermore, we integrated our model into the state space model with nonlinear embedding. This integration allows us to capture hidden dynamics within both the covariate and neural population activities. Using this state space representation, we provided an efficient decoding method for reconstructing the covariate from neural population activities. Our model outperforms other models in prediction and decoding tasks for both synthetic data and place cell spiking activities.

The primary advantage of our model is its capacity to uncover the relationships between observations without the need for prior knowledge or the preparation of parametric models. Simply using our model for decoding can provide insight into whether neural population activities contain sufficient information to recover the observed covariates. This insight is likely to be valuable for researchers when making hypotheses and planning future experiments.

Hereafter, we discuss the main limitations of our model from three perspectives: joint mark intensity evaluation, model interpretation, and model misestimation.

First, our model cannot evaluate intensity values deterministically. Since our joint mark intensity model includes the integral over the latent variable *z*, the evaluation of the intensity value always requires Monte Carlo approximation. This makes the algorithms computationally inefficient. We need sufficient Monte Carlo samples to obtain better gradient estimates for model training and to obtain better reconstructed paths by decoding.

Second, our model does not provide interpretation from the estimated joint mark intensity. Our joint mark intensity model consists of neural networks, which were estimated in a completely data-driven manner. Therefore, we cannot infer the generative structure of the data from the estimated model. Moreover, reflecting prior knowledge of the generative structure in our model is also difficult. In contrast, previous studies have employed a Gaussian mixture for the joint mark intensity based on the observation that neurons have one or more receptive fields and that the waveforms of spikes are distributed according to a unimodal distribution. Previous work [13] developed this idea by introducing a time-varying structure to this mixture expression to deal with neuronal plasticity. Such work has mainly focused on building interpretable models of spike generation and inferred the hidden structure consistent with this interpretation. Unfortunately, our model cannot be used for this purpose.

Third, there is a potential risk that our model will incorrectly estimate the relation between covariates and neural population activities. For example, if there is measurement noise in the electrical potential that depends on a covariate (e.g., the movement of the brain relative to the probe [59]), a deep learning-based model cannot distinguish this false correlation from the actual dependence structure. Such misestimation leads to incorrect conclusions about the neural computation systems in the brain. Imposing appropriate constraints derived from prior knowledge on the model is important to avoid such misestimation. On the other hand, too strong constraints may result in a misspecification of the model. Thus, there is a trade-off between the flexibility of the model and the risk of misestimation. We believe that our model achieves this balance.

To highlight the contribution of this paper from a machine learning perspective, we summarize the recent development of point process models utilizing neural networks. Recent studies have mainly focused on history-dependent temporal point processes. The recurrent marked point process [50] used a recurrent neural network to express the conditional intensity function. Subsequent works have tried to improve the RNN structure [60, 61]. Another study [62] expressed the compensator of the intensity as a neural network to evaluate likelihood directly without numerical integrations. Neural network-based generative models such as VAE [63] and normalizing flows [64, 65] have been used for expressing the event time distribution instead of the conditional intensity. In another line of research, the Neural ODE [66] adopted continuous ordinal differential equations governed by neural networks to express inhomogeneous Poisson process intensity, and Neural JSDE [67] expanded this concept for history-dependent marked point processes. Such ODE-based models have been applied to infer the hidden state dynamics underlying neural population activity [68].

These studies primarily aim to model the history-dependent conditional intensity or inter-event distribution along the time axis. On the other hand, the goal of joint mark intensity estimation is to estimate the dependency structure between state and events. Due to this different goal and for the following technical reasons, these models cannot be used for joint mark intensity estimation. First, since the observations in this paper are multi-dimensional trajectories of states and unsorted spikes, the integration techniques along the time axis used in existing studies are not applicable. Second, the existing models express the mark distribution as a simple parametric distribution whose parameter depends on previous events or hidden states. This assumption is sometimes insufficient for expressing complicated mark distributions. Third, the existing models are designed more for event prediction by incorporating history dependency, making them less effective for decoding.

We conclude this paper by discussing future work.

In the experiments, we concatenated the spike waveforms from all sites into one vector and used it as the spike feature *κ*. Thus, even if two spikes occur at spatially distant recording sites, their spike features may overlap if they occur at temporally close timings. The probability of such spike collisions increases with the number of recording sites. For example, Neuropixels, as discussed in Section 3.6, has a larger number of recording sites than the probes mentioned in Section 3.4, resulting in an increase in the number of spike collisions. Our model assumes that the spike features assigned to each spike are not influenced by other spikes. Therefore, spike collisions violate this assumption and may lead to incorrect estimates of the joint mark intensity. To address this issue, we need to extend our model to incorporate a history-dependent structure, where the distribution of spike features at time *t* depends on previous spike history. One direction for our future work is to integrate our model into a deconvolution method for handling spike collisions (e.g., Kilosort [58]). Solving this collision issue will enable us to apply our model to a wider range of brain regions simultaneously recorded by high-density probes.

Our model succeeded in finding the unknown dependencies between neural population activities and other states in a data-driven manner. This strength leads to a wide range of possible applications, not limited to neuroscience. One possible application candidate is online social activity, such as X or Facebook, which reveal people’s interests and preferences. Our model may enable the prediction of users’ social activities and reconstruct other information that influences their activities.

We integrated our joint mark intensity model into the state space model with a nonlinear embedding. This combination offers a data-driven way to embed the point process observations into the hidden linear dynamics. The same idea has been used in the Koopman operator theory [69]. Models based on the Koopman operator project nonlinear dynamics into linear dynamics in infinite-dimensional space through nonlinear embeddings. Our model could be a promising tool for applying this theory to point process observations.

The point process likelihood appears in different machine learning problems. For instance, conditional density estimation [70] and semi-supervised generative adversarial networks [71] under the generalized Kullback-Leibler divergence maximize the same objective function. Future work includes applying our model to such problems.

## Supporting information

S1 Text Technical appendix. S1 Text contains a further description of our model. We present the derivation of the lower bound for the point process log-likelihood under a general *f*-divergence. Additionally, we provide a detailed explanation of the techniques used in parameter estimation and decoding. Furthermore, we give details of the compared models, the experimental settings, and present additional experimental results.
(PDF)
