# Supplementary material for: Marked point process variational autoencoder with applications to unsorted spiking activities
Source: PLoS Comput Biol. 2024 Dec 30;20(12):e1012620. doi: 10.1371/journal.pcbi.1012620 (PMC11684723; doi:10.1371/journal.pcbi.1012620)
Supplement: S1 Text — contains a further description of our model. We present the derivation of the lower bound for the point process log-likelihood under a general f-divergence. Additionally, we provide a detailed explanation of the techniques used in parameter estimation and decoding. Furthermore, we give details of the compared models, the experimental settings, and present additional experimental results. (PDF) [file pcbi.1012620.s001.pdf]

Supplementary Material

“Marked point process variational autoencoder

with applications to unsorted spiking activities”

Ryohei SHIBUE and Tomoharu IWATA

S1 Details of our model

S1.1 Multiplication constant of joint mark intensity

In Section 2.1, we simplified the point process log-likelihood by omitting the multiplication constant Δt. We provide a justification for this simplification.

Define the scaled joint mark intensity as $\tilde{\lambda}(x, \kappa) = \lambda(x, \kappa)\Delta t$. Then, the point process log-likelihood is rewritten as

$$\sum_{i=1}^{n} \log \lambda(x_i, \kappa_i) - \sum_{j=1}^{m} \int \lambda(x_j, \kappa)\, \Delta t \, \mathrm{d}\kappa = \sum_{i=1}^{n} \log \tilde{\lambda}(x_i, \kappa_i) - \sum_{j=1}^{m} \int \tilde{\lambda}(x_j, \kappa)\, \mathrm{d}\kappa - n \log \Delta t.$$

The last term $-n \log \Delta t$ is a constant and does not affect parameter estimation. Hence, we can ignore this term during parameter estimation. After estimating $\tilde{\lambda}(x, \kappa)$ with the above log-likelihood, we can obtain the estimate of $\lambda(x, \kappa)$ by $\tilde{\lambda}(x, \kappa)/\Delta t$.

S1.2 Derivation of Rényi lower bound and χ upper bound

In this section, we provide the step-by-step derivation of the Rényi lower bound and χ upper bound for $p(x, \kappa)$ and $p(x)$. In this paper, we adopt a slightly different parametrization compared to previous works (e.g., [1]) to simplify the objective function. For example, the parameterization of α in the Rényi lower bound is different from that in the previous paper [2]. To revert to the previous parametrization, replace α with $1 - \alpha$.

Hereafter, we assume that $p(x, \kappa) > 0$ and $p(x) > 0$. If $p(x, \kappa) = 0$, then $p(x, \kappa \mid z)\, p(z)$ is always zero, deriving the lower bound in the main paper as equal to zero. The same applies to $p(x)$ and its upper bound.

First, we consider the Rényi lower bound for $p(x, \kappa)$. Given $0 < \alpha \leq 1$, the power function with order α^{-1} is monotonically increasing. Therefore, the following inequality holds:

$$\begin{aligned} p(x, \kappa) &= (p(x, \kappa)^{\alpha})^{\frac{1}{\alpha}} \\ &\geq (p(x, \kappa)^{\alpha} - \alpha\, p(x, \kappa)^{\alpha}\, \mathcal{H}_{\alpha}(q(z \mid x, \kappa) \parallel p(z \mid x, \kappa)))^{\frac{1}{\alpha}}, \end{aligned} \tag{S1}$$

where $\mathcal{H}_{\alpha}(q(z \mid x, \kappa) \parallel p(z \mid x, \kappa))$ is the α-divergence between $q(z \mid x, \kappa)$ and $p(z \mid x, \kappa)$. The inequality is derived from the fact that the α-divergence is always nonnegative. The equality holds when

$$\mathcal{H}_{\alpha}(q(z \mid x, \kappa) \parallel p(z \mid x, \kappa)) = 0,$$

i.e., $q(z \mid x, \kappa) = p(z \mid x, \kappa)$. The right-hand side of the above inequality is rewritten as the function of the Rényi lower bound. From the definition of the α-divergence, the second term in the bracket is

$$\begin{aligned} \alpha\, p(x, \kappa)^{\alpha}\, \mathcal{H}_{\alpha}(q(z \mid x, \kappa) \parallel p(z \mid x, \kappa)) &= -\int q(z \mid x, \kappa)^{1-\alpha}\, (p(z \mid x, \kappa)\, p(x, \kappa))^{\alpha}\, \mathrm{d}z + p(x, \kappa)^{\alpha} \\ &= -\int q(z \mid x, \kappa) \left(\frac{p(x, \kappa \mid z)\, p(z)}{q(z \mid x, \kappa)} \right)^{\alpha} \mathrm{d}z + p(x, \kappa)^{\alpha} \\ &= -\,\mathrm{E}_{q(z \mid x, \kappa)} \left[\left(\frac{p(x, \kappa \mid z)\, p(z)}{q(z \mid x, \kappa)} \right)^{\alpha} \right] + p(x, \kappa)^{\alpha}. \end{aligned}$$

Substituting this into the inequality (S1) yields the lower bound:

$$p(x, \kappa) \geq \left(\mathrm{E}_{q(z \mid x, \kappa)} \left[\left(\frac{p(x, \kappa \mid z)\, p(z)}{q(z \mid x, \kappa)} \right)^{\alpha} \right] \right)^{\frac{1}{\alpha}}.$$

This lower bound has a one-to-one correspondence with the Rényi lower bound presented in [2]. Taking the logarithm of both sides yields the Rényi lower bound mentioned in that work:

$$\log p(x, \kappa) \geq \frac{1}{\alpha} \log \left(\mathrm{E}_{q(z \mid x, \kappa)} \left[\left(\frac{p(x, \kappa \mid z)\, p(z)}{q(z \mid x, \kappa)} \right)^{\alpha} \right] \right).$$

Next, we consider the χ upper bound for $p(x)$. Given $1 \leq \beta$, the power function with order β^{-1} is

monotonically increasing. Therefore, the following inequality holds:

$$
\begin{aligned}
p(x) &= \left(p(x)^{\beta}\right)^{\frac{1}{\beta}} \\
&\leq \left(p(x)^{\beta} + \beta\, p(x)^{\beta}\, \chi_{\beta}(q(z \mid x) \parallel p(z \mid x))\right)^{\frac{1}{\beta}}, \qquad \text{(S2)}
\end{aligned}
$$

where $\chi_{\beta}(q(z \mid x) \parallel p(z \mid x))$ is the χ-divergence between $q(z \mid x)$ and $p(z \mid x)$. The inequality is derived from the fact that the β-divergence is always nonnegative. The equality holds when

$$
\chi_{\beta}(q(z \mid x) \parallel p(z \mid x)) = 0,
$$

i.e., $q(z \mid x) = p(z \mid x)$. From the definition of the χ-divergence, the second term in the bracket is

$$
\begin{aligned}
\beta\, p(x)^{\beta}\, \chi_{\beta}(q(z \mid x) \parallel p(z \mid x)) &= \int q(z \mid x)^{1-\beta} \left(p(z \mid x)\, p(x)\right)^{\beta} \mathrm{d}z - p(x)^{\beta} \\
&= \int q(z \mid x) \left(\frac{p(x \mid z)\, p(z)}{q(z \mid x)}\right)^{\beta} \mathrm{d}z - p(x)^{\beta} \\
&= \mathrm{E}_{q(z|x)}\left[\left(\frac{p(x \mid z)\, p(z)}{q(z \mid x)}\right)^{\beta}\right] - p(x)^{\beta}.
\end{aligned}
$$

Substituting this into the inequality (S2) yields the upper bound:

$$
p(x) \leq \left(\mathrm{E}_{q(z|x)}\left[\left(\frac{p(x \mid z)\, p(z)}{q(z \mid x)}\right)^{\beta}\right]\right)^{\frac{1}{\beta}}.
$$

S1.3 f variational bound

This section provides the lower bound for the point process log-likelihood under a general f-divergence. An f-divergence is a class of divergence between two probability distributions, determined by a convex function $f : \mathbb{R} \to \mathbb{R}$ that satisfies $f(1) = 0$ [3]:

$$
\mathrm{D}_f(q(z) \parallel p(z)) = \int p(z)\, f\left(\frac{q(z)}{p(z)}\right) \mathrm{d}z.
$$

This divergence is non-negative and equals zero when the densities are identical, i.e., $q(z) = p(z)$. The dual function $f^*(u) = uf(1/u)$ is also convex, satisfies $f^*(1) = 0$, and derives the reverse divergence:

$$
\mathrm{D}_{f^*}(q(z) \parallel p(z)) = \int p(z)\, \frac{q(z)}{p(z)}\, f\left(\frac{p(z)}{q(z)}\right) \mathrm{d}z = \mathrm{D}_f(p(z) \parallel q(z)).
$$

Table A. Examples of f-divergence.

Divergence	$\mathrm{D}_f(q(z) \parallel p(z))$	$f(u)$	$f^*(u)$	$c_f(w)$
Kullback-Leibler divergence, KL	$\int q(z) \log \frac{q(z)}{p(z)} \mathrm{d}z$	$u \log u$	$-\log u$	1
α-divergence, $\mathcal{H}_\alpha$, $0 \leq \alpha \leq 1$	$-\frac{1}{\alpha} \int q(z)^{1-\alpha} p(z)^\alpha \mathrm{d}z + \frac{1}{\alpha}$	$\frac{-u^{1-\alpha} + u}{\alpha}$	$\frac{-u^\alpha + 1}{\alpha}$	w^α
χ-divergence, χ_β, $1 \leq \beta$	$\frac{1}{\beta} \int q(z)^{1-\beta} p(z)^\beta \mathrm{d}z - \frac{1}{\beta}$	$\frac{u^{1-\beta} - u}{\beta}$	$\frac{u^\beta - 1}{\beta}$	w^β

Table A shows examples of f-divergences with specific choices for f. The Kullback-Leibler divergence, α-divergence, and χ-divergence are special cases of f-divergences.

The work by [1] proposed evidence bounds under f-divergences, including lower bounds based on well-known divergences as special cases. This work focused on a set of convex functions that meet particular conditions. Let $\mathcal{F}$ be this set of functions. For more details about these conditions, see the original paper [1].

Hereafter, we utilize two f-divergences determined by convex functions $f, g \in \mathcal{F}$ to derive the evidence bounds for

$$p(x, \kappa) = \int p(x, \kappa \mid z)\, p(z)\, \mathrm{d}z$$

and

$$p(x) = \int p(x \mid z)\, p(z)\, \mathrm{d}z.$$

First, we establish the lower bound for $p(x, \kappa)$. Let f be a convex function in $\mathcal{F}$ such that its dual $f^*(u)$ is monotonically non-increasing. Using the divergence derived from this f, the following inequality holds:

$$\begin{aligned} p(x, \kappa) &= f^{*-1}\left(f^*(p(x, \kappa))\right) \\ &\geq f^{*-1}\left(f^*(p(x, \kappa)) + c_f(p(x, \kappa))\, \mathrm{D}_f(q(z \mid x, \kappa) \parallel p(z \mid x, \kappa))\right) \\ &= f^{*-1}\left(\mathrm{E}_{q(z \mid x, \kappa)}\left[f^*\left(\frac{p(x, \kappa \mid z)\, p(z)}{q(z \mid x, \kappa)}\right)\right]\right). \end{aligned}$$

Here, f^{*-1} is the inverse of f^* satisfying $f^{*-1}(f^*(u)) = u$, $c_f(\cdot)$ is a nonnegative multiplication coefficient determined by f, and $q(z \mid x, \kappa)$ is an encoder. The definitions of $c_f(\cdot)$ for well-known divergences are shown in Table A. The second inequality is derived from the non-negativity of the f-divergence and the monotonically non-increasing nature of f^{*-1}. Maximizing this lower bound with respect to the encoder parameter reduces

to a minimization

$$\mathrm{D}_f(q(z \mid x, \kappa) \parallel p(z \mid x, \kappa)),$$

and the equality holds when $q(z \mid x, \kappa) = p(z \mid x, \kappa)$. Substituting $f^*(u) = (-u^\alpha + 1)/\alpha$ yields the evidence lower bound based on α-divergence:

$$p(x, \kappa) \geq \left(\mathrm{E}_{q(z|x,\kappa)} \left[\left(\frac{p(x, \kappa \mid z)\, p(z)}{q(z \mid x, \kappa)} \right)^\alpha \right] \right)^{\frac{1}{\alpha}}.$$

Next, we derive the upper bound for $p(x)$. Let g be a convex function in $\mathcal{F}$ such that its dual $g^*(u)$ is monotonically non-decreasing. Similarly to the previous derivation, we obtain the following inequality:

$$\begin{aligned} p(x) &= g^{*-1}\left(g^*(p(x))\right) \\ &\leq g^{*-1}\left(g^*(p(x)) + c_g(p(x))\, \mathrm{D}_g(q(z \mid x) \parallel p(z \mid x))\right) \\ &= g^{*-1}\left(\mathrm{E}_{q(z|x)} \left[g^* \left(\frac{p(x \mid z)\, p(z)}{q(z \mid x)} \right) \right] \right). \end{aligned}$$

Here, $c_g(w)$ is a nonnegative multiplication coefficient determined by g and $q(z \mid x)$ is another encoder. The second inequality is derived from the monotonically non-decreasing nature of g^{*-1}. Minimizing this upper bound with respect to the encoder parameter reduces to a minimization

$$\mathrm{D}_g(q(z \mid x) \,||\, p(z \mid x)),$$

and the equality holds when $q(z \mid x) = p(z \mid x)$. Substituting $g^*(u) = (u^\beta - 1)/\beta$ yields the χ upper bound:

$$p(x) \leq \left(\mathrm{E}_{q(z|x)} \left[\left(\frac{p(x \mid z)\, p(z)}{q(z \mid x)} \right)^\beta \right] \right)^{\frac{1}{\beta}}.$$

Using these two bounds, the lower bound of the point process log-likelihood is

$$\sum_{i=1}^{n} \log\left(\lambda_0\, p(x_i, \kappa_i)\right) - \sum_{j=1}^{m} \lambda_0\, p(x_j)$$
$$\geq \sum_{i=1}^{n} \log\left(\lambda_0\, f^{*-1}\left(\mathrm{E}_{q(z|x_i,\kappa_i)}\left[f^{*}\left(\frac{p(x_i, \kappa_i \mid z)\, p(z)}{q(z \mid x_i, \kappa_i)}\right)\right]\right)\right)$$
$$- \sum_{j=1}^{m} \lambda_0\, g^{*-1}\left(\mathrm{E}_{q(z|x_j)}\left[g^{*}\left(\frac{p(x_j \mid z)\, p(z)}{q(z \mid x_j)}\right)\right]\right).$$

Along with the parameter estimation, we need to replace the expectations in this lower bound with Monte Carlo estimates. Utilizing the reparameterization trick, the Monte Carlo estimate for this lower bound is

$$\sum_{i=1}^{n} \log\left(\lambda_0\, f^{*-1}\left(\frac{1}{L}\sum_{l=1}^{L} f^{*}\left(\frac{p(x_i, \kappa_i \mid z_{il})\, p(z_{il})}{q(z_{il} \mid x_i, \kappa_i)}\right)\right)\right) - \sum_{j=1}^{m} \lambda_0\, g^{*-1}\left(\frac{1}{L}\sum_{l=1}^{L} g^{*}\left(\frac{p(x_j \mid z_{jl})\, p(z_{jl})}{q(z_{jl} \mid x_j)}\right)\right),$$

where $\{z_{il}\}_{l=1}^{L}$ and $\{z_{jl}\}_{l=1}^{L}$ are i.i.d. samples from $q(z \mid x_i, \kappa_i)$ and $q(z \mid x_j)$, respectively. If the functions $\log \circ f^{*-1}$ and $-\,g^{*-1}$ are concave, then the expectation of this Monte Carlo estimate is also a lower bound for the point process log-likelihood. However, this does not hold since $-g^{*-1}$ is convex, which leads to a positive bias in the Monte Carlo estimate of this term. Hence, the maximization of this Monte Carlo estimate is not guaranteed to be a maximization of the point process log-likelihood. To reduce this kind of bias, we use the resampling estimator described in Section S1.4.

S1.4 Jackknife estimator

This section describes the bias correction for the Monte Carlo estimate of the lower bound. While this technique is applicable to any f-divergence, we specifically focus on the lower bound derived from the Rényi lower bound and the χ upper bound.

Since the log function is concave, the expectation of the Monte Carlo estimate of the Rényi lower bound

$$\mathrm{E}_{z_1,\ldots,z_L \sim q(z|x,\kappa)}\left[\frac{1}{\alpha}\log\left(\frac{1}{L}\sum_{l=1}^{L}\left(\frac{\lambda_0\, p(x, \kappa \mid z_l)\, p(z_l)}{q(z_l \mid x, \kappa)}\right)^{\alpha}\right)\right]$$

is also the lower bound of the original Rényi lower bound. On the other hand, the expectation of the Monte

Carlo estimate of the χ upper bound

$$\mathrm{E}_{z_1,\ldots,z_L\sim q(z|x)}\left[\left(\frac{1}{L}\sum_{l=1}^{L}\left(\frac{\lambda_0\, p(x\mid z_l)\, p(z_l)}{q(z_l\mid x)}\right)^{\beta}\right)^{\frac{1}{\beta}}\right]$$

is not necessarily the upper bound of the χ upper bound. This is because the power function with order $1/\beta$ is concave, not convex. Consequently, the expectation of the objective function (3) in the main paper is not guaranteed to be a lower bound of the point process log-likelihood. This may cause misestimation of the parameter since maximizing this objective is not equivalent to maximizing the point process log-likelihood. This issue becomes more critical when the bias between the lower bound and its Monte Carlo estimate is large. To reduce such bias, we adopt the jackknife estimator for the χ upper bound.

The jackknife resampling technique involves a leave-one-out resampling approach to eliminate the $O(L^{-1})$ bias from the estimator evaluated on L samples [4]. Previous work has used this technique to reduce the bias of the Rényi lower bound [5].

Our primary goal is to eliminate the bias between the χ upper bound and its Monte Carlo estimate. Let W and W_L be the χ upper bound and its Monte Carlo estimate based on L samples, respectively:

$$W=\left(\mathrm{E}\left[\left(\frac{\lambda_0\, p(x\mid z)\, p(z)}{q(z\mid x)}\right)^{\beta}\right]\right)^{\frac{1}{\beta}},\quad W_L=\left(\frac{1}{L}\sum_{l=1}^{L}\left(\frac{\lambda_0\, p(x\mid z_l)\, p(z_l)}{q(z_l\mid x)}\right)^{\beta}\right)^{\frac{1}{\beta}}.$$

Here, the expectation in the first definition is taken over z distributed according to $q(z\mid x)$, and $\{z_l\}_{l=1}^{L}$ in the second definition are i.i.d. random samples from $q(z\mid x)$. This W_L is the consistent estimator of W as $L\to\infty$, and its expectation can be asymptotically expanded as

$$\mathrm{E}[W_L]=W+\frac{c_1}{L}+\frac{c_2}{L^2}+\cdots,$$

where c_i, $i=1,2,\ldots$ are coefficients that do not depend on L. The jackknife estimator aims to reduce the $O(L^{-1})$ bias term by using resampling. Let W_{L-1} be the consistent estimator that can be expanded in the same way. With this W_{L-1}, the jackknife estimator of W_L is defined as

$$\widehat{W}_L := LW_L-(L-1)W_{L-1}.$$

The expectation of this estimator is

$$\mathrm{E}\left[LW_L - (L-1)W_{L-1}\right] = W + O(L^{-2})$$

and does not include the $O(L^{-1})$ bias. There are several ways to define the W_{L-1} from L samples, and the most commonly used of these is the leave-one-out estimator

$$W_{L-1} := \frac{1}{L}\sum_{l=1}^{L}\left(\frac{1}{L-1}\sum_{k\neq l}\left(\frac{\lambda_0\, p(x \mid z_k)\, p(z_k)}{q(z_k \mid x)}\right)^{\beta}\right)^{\frac{1}{\beta}}.$$

This choice is optimal in the sense that it minimizes the variance of the estimator [6].

In conclusion, the jackknife estimator for the χ upper bound is

$$L\left(\frac{1}{L}\sum_{l=1}^{L}\left(\frac{\lambda_0\, p(x \mid z_l)\, p(z_l)}{q(z_l \mid x)}\right)^{\beta}\right)^{\frac{1}{\beta}} - \frac{L-1}{L}\sum_{l=1}^{l}\left(\frac{1}{L-1}\sum_{k\neq l}\left(\frac{\lambda_0\, p(x \mid z_k)\, p(z_k)}{q(z_k \mid x)}\right)^{\beta}\right)^{\frac{1}{\beta}}.$$

This modification is expected to reduce the bias and yield improved results for parameter estimation.

A higher-order jackknife estimator can be constructed to reduce higher-order bias. The m-th order jackknife estimator is

$$\sum_{j=0}^{m}\frac{c(L,m,j)}{\binom{L}{L-j}}\sum_{|I|=L-j,\, I\subset\{1,\ldots,L\}}\left(\frac{1}{|I|}\sum_{k\in I}\left(\frac{\lambda_0\, p(x \mid z_k)\, p(z_k)}{q(z_k \mid x)}\right)^{\beta}\right)^{\frac{1}{\beta}},$$

where $c(L,m,j)$ is a multiplication constant:

$$c(L,m,j) = (-1)^j\,\frac{(L-j)^m}{j!(m-j)!}.$$

The m-th order jackknife estimator removes the $O(L^{-m})$ bias. However, due to the computational cost of $O(L^m)$, this estimator becomes impractical for large L.

We do not recommend using the jackknife estimator for the Rényi lower bound, since it will be unstable when the mark dimension is high. Consider the case where $L=2$ and the importance weights

$$v_1 := \frac{\lambda_0\, p(x,\kappa \mid z_1)\, p(z_1)}{q(z_1 \mid x,\kappa)},\quad v_2 := \frac{\lambda_0\, p(x,\kappa \mid z_2)\, p(z_2)}{q(z_2 \mid x,\kappa)}$$

have a large difference, i.e., $v_1 \gg v_2$. In this case, the jackknife estimator for the Rényi lower bound can be

approximated as follows:

$$\frac{2}{\alpha}\log\left(\frac{v_1^\alpha + v_2^\alpha}{2}\right) - \frac{1}{2}\left(\frac{1}{\alpha}\log v_1^\alpha + \frac{1}{\alpha}\log v_2^\alpha\right) \approx \frac{3}{2}\log v_1 - \frac{1}{2}\log v_2.$$

The right-hand side of this equation includes the negative sign of the log importance weight $-\log v_2$. When the mark dimension is high, the importance weights defined by Gaussian density often become very small. In such cases, the absolute value of $\log v_2$ becomes much larger than $\log v_1$, leading the estimator to be nearly equal to $-\log v_2$. Consequently, maximizing this objective minimizes the $\log v_2$, which can lead to a deviation from the true parameter. A similar issue arises in general L when $v_1, \ldots, v_{L-1} \gg v_L$.

This issue does not occur when using the jackknife estimator for the χ upper bound. Consider the similar case where $L = 2$ and importance weights

$$w_1 := \frac{\lambda_0\, p(x \mid z_1)\, p(z_1)}{q(z_1 \mid x)}, \quad w_2 := \frac{\lambda_0\, p(x \mid z_2)\, p(z_2)}{q(z_2 \mid x)}$$

have a large difference $w_1 \gg w_2$. Then, the jackknife estimator of the χ upper bound is approximated as

$$2\left(\frac{w_1^\beta + w_2^\beta}{2}\right)^{\frac{1}{\beta}} - \frac{1}{2}\left(\left(w_1^\beta\right)^{\frac{1}{\beta}} + \left(w_2^\beta\right)^{\frac{1}{\beta}}\right) \approx \left(2^{1-\frac{1}{\beta}} - \frac{1}{2}\right) w_1 - \frac{1}{2} w_2.$$

Although the right-hand side of this equation contains the negative sign of the importance weight $-w_2$, this term vanishes when $w_2 \approx 0$ and does not affect the gradients of the parameters.

S1.5 Doubly reparameterized gradients

This section describes the doubly reparameterized gradients used in parameter estimation. The lower bound of the point process log-likelihood consists of the Rényi lower bound and the χ upper bound. These two evidence bounds are written as

$$\frac{1}{\alpha}\log\left(\frac{1}{L}\sum_{l=1}^{L} v_l^\alpha\right), \quad \left(\frac{1}{L}\sum_{l=1}^{L} w_l^\beta\right)^{\frac{1}{\beta}},$$

where v_l and w_l are importance weights defined as

$$v_l = \frac{\lambda_0\, p(x, \kappa \mid z_l)\, p(z_l)}{q(z_l \mid x, \kappa)}, \quad w_l = \frac{\lambda_0\, p(x \mid z_l)\, p(z_l)}{q(z_l \mid x)}.$$

In the estimation step, we need to compute the gradients of these two bounds with respect to the encoder parameter ϕ. The common approach to achieve this is the reparameterization trick. The reparameterization trick treats the latent variable z_l as a deterministic function of the random variable ϵ_l and the parameter ϕ. For example, under the Gaussian assumption in our paper, the reparameterization of z_l in the χ upper bound is

$$z_l := z_\phi(\epsilon_l) = \nu_\phi^{\mathrm{x}\to\mathrm{z}}(x) + \mathrm{chol}\left(\Xi_\phi^{\mathrm{x}\to\mathrm{z}}(x)\right)^{-\top} \epsilon_l,$$

where $\mathrm{chol}(\cdot)$ is a Cholesky decomposition operator and ϵ_l is a random variable that follows the Gaussian distribution $\mathrm{N}(0, I)$. By using this reparameterization, the lower bounds can be written as functions of ϕ, enabling us to calculate the gradients through auto-differentiation tools. However, when using evidence lower bounds based on general divergences, this reparameterization can lead to instability in the gradient estimator. To address this issue, we utilize the doubly reparameterized gradient estimator proposed by [7].

We review the doubly reparameterized gradient using the χ upper bound as an example. The gradient of the χ upper bound with respect to the encoder parameter ϕ is

$$\nabla_\phi \mathbb{E}_{\{z_l\}_{l=1}^L}\left[\left(\frac{1}{L}\sum_{l=1}^{L} w_l^\beta\right)^{\frac{1}{\beta}}\right] = \mathbb{E}_{\{\epsilon_l\}_{l=1}^L}\left[\sum_{l=1}^{L}\left(\frac{1}{L}\sum_{k=1}^{L} w_k^\beta\right)^{\frac{1}{\beta}} \frac{w_l^\beta}{\sum_{k=1}^{L} w_k^\beta}\nabla_\phi \log w_l\right]. \tag{S3}$$

As explained in the previous paragraph, we treat $z_l = z_\phi(\epsilon_l)$ as a deterministic function that receives ϕ and random variables $\epsilon_l \sim \mathrm{N}(0, I)$ as inputs. The total derivative term $\nabla_\phi \log w_l$ can be split into two terms:

$$\nabla_\phi \log w_l = -\frac{\partial}{\partial\phi}\log q(z_l \mid x) + \frac{\partial \log w_l}{\partial z_l}\frac{\partial z_l}{\partial \phi}.$$

This means that the encoder parameter ϕ affects $\nabla_\phi \log w_l$ in two ways: through the log probability of the encoder, $\log q(z \mid x)$, and through the function $z_l = z_\phi(\epsilon_l)$. Previous work [8] has suggested that the first term exhibits large variance and is the primary cause of the gradient estimator's instability. To reduce this variance, the work by [7] proposed the doubly reparameterized gradient estimator.

The core idea of this method is to estimate the first term by employing an additional reparameterization trick:

$$\mathrm{E}_{z_l}\left[\left(\left(\frac{1}{L}\sum_{k=1}^{L} w_k^\beta\right)^{\frac{1}{\beta}} \frac{w_l^\beta}{\sum_{k=1}^{L} w_k^\beta}\right)\frac{\partial}{\partial\phi}\log q(z_l \mid x)\right] = \mathrm{E}_{\epsilon_l}\left[\frac{\partial}{\partial z_l}\left(\left(\frac{1}{L}\sum_{k=1}^{L} w_k^\beta\right)^{\frac{1}{\beta}} \frac{w_l^\beta}{\sum_{k=1}^{L} w_k^\beta}\right)\frac{\partial z_l}{\partial\phi}\right].$$

Substituting this equivalence into (S3) derives the doubly reparameterized gradients of the χ upper bound:

$$\begin{aligned}
&\nabla_\phi \mathbb{E}_{\{z_l\}_{l=1}^L}\left[\left(\frac{1}{L}\sum_{l=1}^L w_l^\beta\right)^{\frac{1}{\beta}}\right] \\
&\qquad = \mathrm{E}_{\{\epsilon_l\}_{l=1}^L}\left[\sum_{l=1}^L (\beta-1)\left(\frac{1}{L}\sum_{k=1}^L w_k^\beta\right)^{\frac{1}{\beta}}\left(\left(\frac{w_l^\beta}{\sum_{k=1}^L w_k^\beta}\right)^2 - \frac{w_l^\beta}{\sum_{k=1}^L w_k^\beta}\right)\frac{\partial \log w_l}{\partial z_l}\frac{\partial z_l}{\partial \phi}\right].
\end{aligned}$$

Note that this reparameterized gradient is applicable to the jackknife estimator discussed in the previous section.

The same derivation can be used for the Rényi lower bound. The reparameterized gradient of the Rényi lower bound is

$$\nabla_\phi \mathbb{E}_{\{z_l\}_{l=1}^L}\left[\frac{1}{\alpha}\log\left(\frac{1}{L}\sum_{l=1}^L v_l^\alpha\right)\right] = \mathrm{E}_{\{\epsilon_l\}_{l=1}^L}\left[\sum_{l=1}^L\left(\alpha\left(\frac{v_l^\alpha}{\sum_{k=1}^L v_k^\alpha}\right)^2 + (1-\alpha)\frac{v_l^\alpha}{\sum_{k=1}^L v_k^\alpha}\right)\frac{\partial \log v_l}{\partial z_l}\frac{\partial z_l}{\partial \phi}\right].$$

S1.6 Sampling hidden states from encoder

This section demonstrates how to generate samples from the variational posterior $q(\boldsymbol{x} \mid \boldsymbol{y}, \boldsymbol{\kappa})$ and how to evaluate their log density values. We assume that the density of the encoder $q(\boldsymbol{x} \mid \boldsymbol{y}, \boldsymbol{\kappa})$ is proportional to the product of the densities:

$$q(\boldsymbol{x} \mid \boldsymbol{y}, \boldsymbol{\kappa}) \propto q(x_1)\prod_{r=2}^R q(x_r \mid x_{r-1})\prod_{r=1}^R q(x_r \mid y_r)\prod_{r=1}^R q(x_r \mid \boldsymbol{\kappa}_r).$$

To sample from the left distribution, we need to select these densities in such a way that the normalized density can be easily calculated. To achieve this, we define these densities as Gaussian distributions:

$$\begin{aligned}
q(x_1) &= \mathrm{N}(x_1 \mid b_1, W_1), \\
q(x_r \mid x_{r-1}) &= \mathrm{N}(x_r \mid G x_{r-1} + b, W), \\
q(x_r \mid y_r) &= \mathrm{N}(x_r \mid \nu_\phi^{\mathrm{y}\to\mathrm{x}}(y_r), \Xi_\phi^{\mathrm{y}\to\mathrm{x}}(y_r)), \\
q(x_r \mid \boldsymbol{\kappa}_r) &= \left(\prod_{i=1}^{n_r}\mathrm{N}(x_r \mid \nu_\phi^{\kappa\to\mathrm{x}}(\kappa_{ri}), \Xi_\phi^{\kappa\to\mathrm{x}}(\kappa_{ri}))\right)\mathrm{N}(x_r \mid \nu_\phi^{\mathrm{x}}, \Xi_\phi^{\mathrm{x}}).
\end{aligned}$$

This assumption makes the variational posterior of $\boldsymbol{x}$ also a multivariate Gaussian distribution:

$$q(\boldsymbol{x} \mid \boldsymbol{y}, \boldsymbol{\kappa}) = \mathrm{N}(\boldsymbol{x} \mid \boldsymbol{\nu}, \boldsymbol{\Xi}), \tag{S4}$$

where $\boldsymbol{\nu}$ and $\boldsymbol{\Xi}$ are the mean and precision matrix calculated from the above densities. Once we obtain these $\boldsymbol{\nu}$ and $\boldsymbol{\Xi}$, we can generate samples from this distribution using the reparameterization trick:

$$\boldsymbol{x} = \boldsymbol{\nu} + \boldsymbol{L}^{-\top} \boldsymbol{\epsilon}, \quad \boldsymbol{\epsilon} \sim \mathrm{N}(\boldsymbol{0}, \boldsymbol{I}),$$

where $\boldsymbol{L}$ is a Cholesky decomposition of $\boldsymbol{\Xi}$. In addition, we can evaluate the log densities of these samples as

$$\begin{aligned} \log q(\boldsymbol{x} \mid \boldsymbol{y}, \boldsymbol{\kappa}) &= \frac{1}{2} \log\det \boldsymbol{\Xi} - \frac{1}{2} (\boldsymbol{x} - \boldsymbol{\nu})^\top \boldsymbol{\Xi} (\boldsymbol{x} - \boldsymbol{\nu}) + \text{const.} \\ &= \log\det \boldsymbol{L} - \frac{1}{2} \boldsymbol{\epsilon}^\top \boldsymbol{\epsilon} + \text{const.} \end{aligned}$$

However, the above operations require $O(R^3)$, which is computationally expensive. Nevertheless, by utilizing the block tridiagonal structure of the precision matrix $\boldsymbol{\Xi}$, these computational costs can be reduced to $O(R)$ [9].

Hereafter, we explain the details of this computation. First, we define the following matrices:

$$\boldsymbol{C}_\phi = \begin{bmatrix} \Xi_\phi^{\mathrm{y}\to\mathrm{x}}(y_1) + \sum_{i=1}^{n_1} \Xi_\phi^{\kappa\to\mathrm{x}}(\kappa_{1i}) + \Xi_\phi^{\mathrm{x}} & & \\ & \ddots & \\ & & \Xi_\phi^{\mathrm{y}\to\mathrm{x}}(y_R) + \sum_{i=1}^{n_R} \Xi_\phi^{\kappa\to\mathrm{x}}(\kappa_{Ri}) + \Xi_\phi^{\mathrm{x}} \end{bmatrix},$$

$$\boldsymbol{d}_\phi = \begin{bmatrix} \Xi_\phi^{\mathrm{y}\to\mathrm{x}}(y_1)\, \nu_\phi^{\mathrm{y}\to\mathrm{x}}(y_1) + \sum_{i=1}^{n_1} \Xi_\phi^{\kappa\to\mathrm{x}}(\kappa_{1i})\, \nu_\phi^{\kappa\to\mathrm{x}}(\kappa_{1i}) + \Xi_\phi^{\mathrm{x}}\, \nu_\phi^{\mathrm{x}} \\ \vdots \\ \Xi_\phi^{\mathrm{y}\to\mathrm{x}}(y_R)\, \nu_\phi^{\mathrm{y}\to\mathrm{x}}(y_R) + \sum_{i=1}^{n_R} \Xi_\phi^{\kappa\to\mathrm{x}}(\kappa_{Ri})\, \nu_\phi^{\kappa\to\mathrm{x}}(\kappa_{Ri}) + \Xi_\phi^{\mathrm{x}}\, \nu_\phi^{\mathrm{x}} \end{bmatrix},$$

$$\boldsymbol{G} = \begin{bmatrix} I & & & \\ -G & I & & \\ & \ddots & \ddots & \\ & & -G & I \end{bmatrix}, \quad \boldsymbol{W} = \begin{bmatrix} W_1 & & & \\ & W & & \\ & & \ddots & \\ & & & W \end{bmatrix}, \quad \boldsymbol{b} = \begin{bmatrix} b_1 \\ b \\ \vdots \\ b \end{bmatrix}.$$

Then, based on the Gaussian property, the mean $\boldsymbol{\nu}$ and precision matrix $\boldsymbol{\Xi}$ of (S4) are given by

$$\boldsymbol{\Xi} = \boldsymbol{C}_\phi + \boldsymbol{G}^\top \boldsymbol{W} \boldsymbol{G},$$
$$\boldsymbol{\nu} = \boldsymbol{\Xi}^{-1} \left(\boldsymbol{d}_\phi + \boldsymbol{G}^\top \boldsymbol{W} \boldsymbol{b} \right).$$

The latter equation includes the multiplication of the inverse of $\boldsymbol{\Xi}$, whose size is proportional to the number of time bins R. The naive matrix operation incurs a computational cost of $O(R^3)$; however, this can be reduced to $O(R)$ by using Cholesky decomposition:

$$\boldsymbol{\Xi} = \boldsymbol{L}\boldsymbol{L}^\top.$$

By utilizing the block tridiagonal structure of $\boldsymbol{\Xi}$, this decomposition incurs only a cost of $O(R)$. With this decomposition, multiplying $\boldsymbol{\Xi}^{-1} = (\boldsymbol{L}\boldsymbol{L}^\top)^{-1}$ by a vector $\boldsymbol{\rho}$ is equivalent to solving the following two-step linear equations:

$$\boldsymbol{L}^{-\top} \left(\boldsymbol{L}^{-1} \boldsymbol{\rho} \right) = \boldsymbol{\nu} \quad \Leftrightarrow \quad \boldsymbol{L}\boldsymbol{\tau} = \boldsymbol{\rho}, \quad \boldsymbol{L}^\top \boldsymbol{\nu} = \boldsymbol{\tau}.$$

Since the Cholesky decomposition of a block tridiagonal matrix is a block bidiagonal matrix, these linear equations can be efficiently solved using forward and backward substitutions, with a cost of $O(R)$. The second linear equation is also utilized in the reparameterization trick:

$$\boldsymbol{x} = \boldsymbol{\nu} + \boldsymbol{L}^{-\top} \boldsymbol{\epsilon}, \quad \boldsymbol{\epsilon} \sim \mathrm{N}(\boldsymbol{0}, \boldsymbol{I}),$$

and this operation costs $O(R)$. In conclusion, all operations related to the encoder $q(\boldsymbol{x} \mid \boldsymbol{y}, \boldsymbol{\kappa})$ can be performed with $O(R)$ cost.

In the remaining part of this section, we describe the details of the iteration algorithms used in the preceding operations. First, we show the recursive formula for the Cholesky decomposition of $\boldsymbol{\Xi}$. Given that $\boldsymbol{\Xi}$ is a block tridiagonal matrix and its Cholesky decomposition is thus a bidiagonal matrix, we define the

elements of these matrices as follows:

$$\boldsymbol{\Xi} = \begin{bmatrix} \Xi_1 & \Pi_1^\top & & \\ \Pi_1 & \Xi_2 & \ddots & \\ & \ddots & \ddots & \Pi_{R-1}^\top \\ & & \Pi_{R-1} & \Xi_R \end{bmatrix}, \quad \boldsymbol{L} = \begin{bmatrix} L_1 & & & \\ M_1 & L_2 & & \\ & \ddots & \ddots & \\ & & M_{R-1} & L_R \end{bmatrix}.$$

Then, L_r and M_r are obtained by the following recursive formula from $r = 1$ to R:

$$L_r \leftarrow \begin{cases} \operatorname{chol}\left(\Xi_r\right), & (r = 1), \\ \operatorname{chol}\left(\Xi_r - M_{r-1}M_{r-1}^\top\right), & (\text{otherwise}), \end{cases}$$
$$M_r \leftarrow \Pi_r L_r^{-\top},$$

where chol$(\cdot)$ is the Cholesky decomposition function.

Next, we show the forward and backward substitutions to solve the linear equations with coefficient matrices $\boldsymbol{L}$ and $\boldsymbol{L}^\top$. Define the elements of the vectors $\boldsymbol{\nu}, \boldsymbol{\tau}, \boldsymbol{\rho}$ as

$$\boldsymbol{\nu} = \begin{bmatrix} \nu_1 \\ \nu_2 \\ \vdots \\ \nu_R \end{bmatrix}, \quad \boldsymbol{\tau} = \begin{bmatrix} \tau_1 \\ \tau_2 \\ \vdots \\ \tau_R \end{bmatrix}, \quad \boldsymbol{\rho} = \begin{bmatrix} \rho_1 \\ \rho_2 \\ \vdots \\ \rho_R \end{bmatrix},$$

and consider the linear equations

$$\boldsymbol{L}\boldsymbol{\tau} = \boldsymbol{\rho}, \quad \boldsymbol{L}^\top \boldsymbol{\nu} = \boldsymbol{\tau}.$$

Then, the forward substitution from $r = 1$ to R to solve the first equation is

$$\tau_r \leftarrow \begin{cases} L_r^{-1}\rho_r, & (r = 1), \\ L_r^{-1}\left(\rho_r - M_{r-1}\tau_{r-1}\right), & (\text{otherwise}), \end{cases}$$

and the backward substitution from $r = R$ to 1 to solve the second equation is

$$\nu_r \leftarrow \begin{cases} L_r^{-\top} \tau_r, & (r = R), \\ L_r^{-\top} \left(\tau_r - M_r^{\top} \nu_{r+1} \right), & \text{(otherwise)}. \end{cases}$$

Finally, the log determinant of $\boldsymbol{\Xi}$ can be obtained through the bidiagonal structure of $\boldsymbol{L}$:

$$\log \det \boldsymbol{\Xi} = 2 \log \det \boldsymbol{L} = 2 \sum_{r=1}^{R} \log \det L_r.$$

Here, since L_r is a lower triangular matrix, $\log \det L_r$ is the summation of the logarithms of the diagonal elements of L_r.

S1.7 Parameter estimation

This section summarizes the flow of parameter estimation within our model. We aim to maximize the lower bound of the log-likelihood $\log p(\boldsymbol{y}, \boldsymbol{\kappa})$. To compute this lower bound, we employ three encoders. First, we use the encoder $q(\boldsymbol{x} \mid \boldsymbol{y}, \boldsymbol{\kappa})$ to obtain the lower bound as

$$\log p(\boldsymbol{y}, \boldsymbol{\kappa}) \geq \mathrm{E}_{q(\boldsymbol{x}|\boldsymbol{y},\boldsymbol{\kappa})} \left[\log p(\boldsymbol{y} \mid \boldsymbol{x}) + \log p(\boldsymbol{\kappa} \mid \boldsymbol{x}) + \log p(\boldsymbol{x}) - \log q(\boldsymbol{x} \mid \boldsymbol{y}, \boldsymbol{\kappa}) \right], \tag{S5}$$

where $p(\boldsymbol{y} \mid \boldsymbol{x})$ is the decoder for $\boldsymbol{y}$, $p(\boldsymbol{\kappa} \mid \boldsymbol{x})$ is the point process likelihood, and $p(\boldsymbol{x})$ is a prior for $\boldsymbol{x}$. While the values of the decoder $p(\boldsymbol{y} \mid \boldsymbol{x})$, prior $p(\boldsymbol{x})$, and posterior $q(\boldsymbol{x} \mid \boldsymbol{y}, \boldsymbol{\kappa})$ are computable, the point process likelihood $p(\boldsymbol{\kappa} \mid \boldsymbol{x})$ is not analytically tractable since it contains expectations over z. To derive a lower bound for this part, we introduce the encoders $q(z \mid x, \kappa)$ and $q(z \mid x)$:

$$\begin{aligned} &\log p(\boldsymbol{\kappa} \mid \boldsymbol{x}) \\ &= \sum_{r=1}^{R} \left[\sum_{i=1}^{n_r} \log \lambda(x_r, \kappa_{ri}) - \lambda(x_r) \right] \\ &\geq \sum_{r=1}^{R} \left[\sum_{i=1}^{n_r} \frac{1}{\alpha} \log \mathrm{E}_{q(z|x_r,\kappa_{ri})} \left[\left(\frac{\lambda_0\, p(x_r, \kappa_{ri} \mid z)\, p(z)}{q(z \mid x_r, \kappa_{ri})} \right)^{\alpha} \right] - \left(\mathrm{E}_{q(z|x_r)} \left[\left(\frac{\lambda_0\, p(x_r \mid z)\, p(z)}{q(z \mid x_r)} \right)^{\beta} \right] \right)^{\frac{1}{\beta}} \right]. \end{aligned}$$

Substituting this into the initial equation (S5) yields the objective lower bound.

To perform optimization, the expectations with respect to $\boldsymbol{x}$ and z in this lower bound should be replaced by Monte Carlo estimates. To do this, we first sample $\boldsymbol{x}$ from encoder $q(\boldsymbol{x} \mid \boldsymbol{y}, \boldsymbol{\kappa})$ as explained in Section S1.6.

Then, given this $\boldsymbol{x}$, we sample $\{z_{ril}\}$ and $\{z_{rl}\}$ from $q(z \mid x_r, \kappa_{ri})$ and $q(z \mid x_r)$, respectively. Given these samples, we replace the Rényi lower bound by its Monte Carlo estimate

$$\frac{1}{\alpha} \log \left(\frac{1}{L} \sum_{l=1}^{L} \left(\frac{\lambda_0 \, p(x_r, \kappa_{ri} \mid z_{ril}) \, p(z_{ril})}{q(z_{ril} \mid x_r, \kappa_{ri})} \right)^{\alpha} \right),$$

and the χ upper bound by the jackknife estimator

$$\sum_{j=0}^{m} \frac{c(L, m, j)}{\binom{L}{L-j}} \sum_{|I|=L-j,\, I \subset \{1, \dots, L\}} \left(\frac{1}{|I|} \sum_{k \in I} \left(\frac{\lambda_0 \, p(x \mid z_k) \, p(z_k)}{q(z_k \mid x)} \right)^{\beta} \right)^{\frac{1}{\beta}}.$$

In the main paper, we assume Gaussian distributions for both encoders and decoders. Fig A visually represents the computational graph based on our model assumptions. According to this graph, parameter gradients are computed using automatic differentiation in deep neural network libraries. The gradients of encoder parameters appearing in the point process log-likelihood are replaced by doubly reparameterized gradients. Based on these gradients, we iteratively update the parameters using the Adam optimizer.

S1.8 Decoding

This section describes how to reconstruct the observed covariate $\boldsymbol{y}$ and the hidden state $\boldsymbol{x}$ from the unsorted spikes $\boldsymbol{\kappa}$. As stated in the main paper, we rely on the Laplace approximation to approximate the posterior $p(\boldsymbol{x} \mid \boldsymbol{\kappa})$. However, the Laplace approximation requires the posterior density to be concave with respect to $\boldsymbol{x}$, which is not the case in our model. To overcome this limitation, we adopt the Gauss-Newton approximation for the point process log-likelihood.

We first review the Gauss-Newton optimization [10]. This method is used for optimizing nonlinear objective functions that can be represented as a composite of a smooth map and a concave function, denoted as

$$\pi_F(x) := \pi(F(x)),$$

where π is a concave function, F is a smooth map, and $\pi_F(x)$ is a composite function that we want to maximize. The core idea here is to approximate $\pi_F(x)$ with a concave function by approximating $F(x)$ with a first-order Taylor expansion around $\bar{x}$ as

$$\widehat{\pi_F}(x) := \pi(F(\bar{x}) + \mathrm{J}_F(\bar{x})(x - \bar{x})).$$

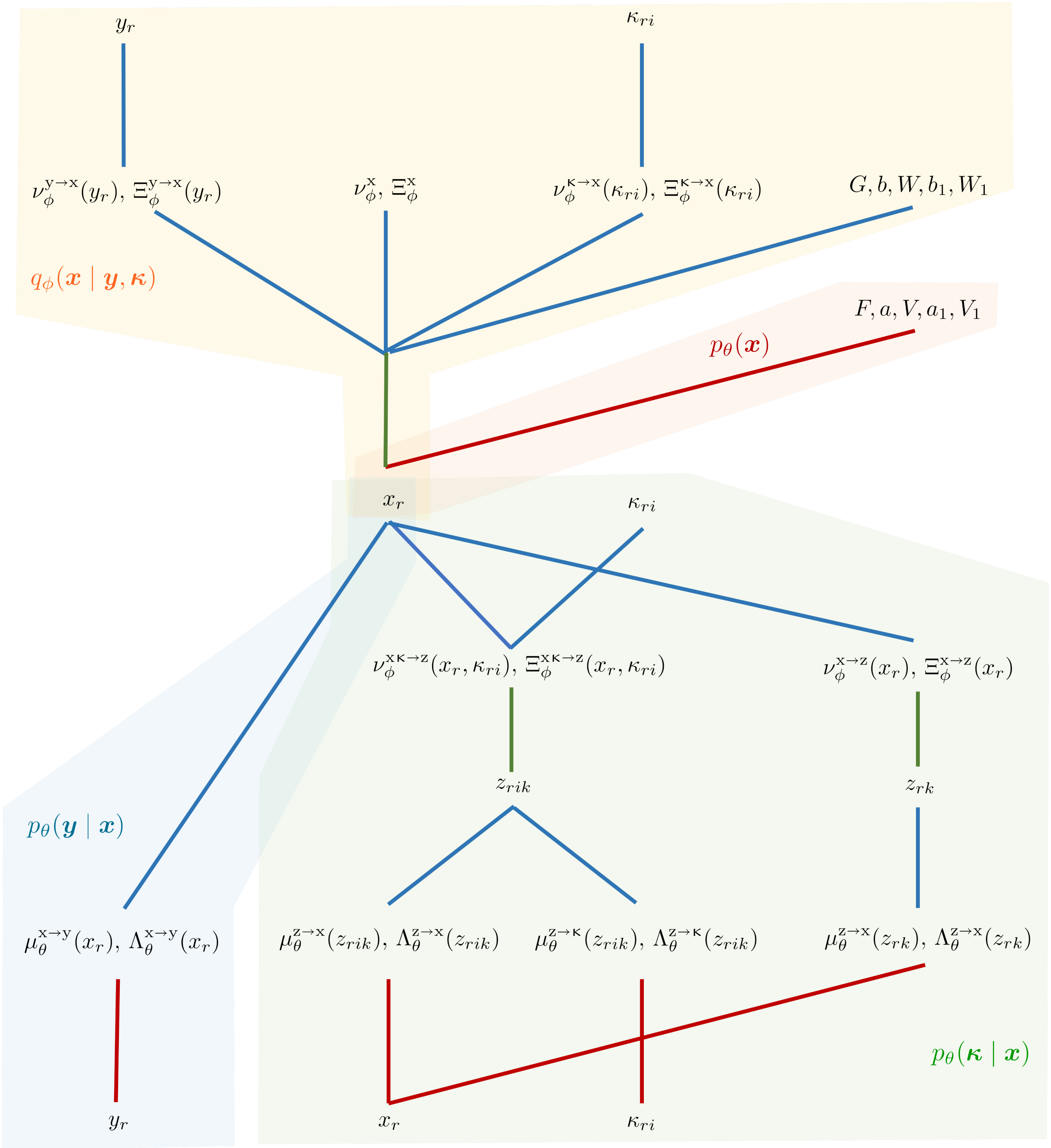

Fig A. Computational graph of our model. Blue lines indicate deterministic paths, green lines indicate sampling, and red lines indicate density evaluation. With this graph and the reparameterization trick, we calculate the gradients of the parameters with automatic differentiation tools.

where $\mathrm{J}_F(\bar{x})$ is the Jacobian matrix of F at $\bar{x}$. Since π is concave and the inner part of this function is a linear function of x, $\widehat{\pi_F}(x)$ becomes a concave function of x. This concavity is derived from the fact that the Hessian matrix at $\bar{x}$ is

$$\nabla^2 \widehat{\pi_F}(\bar{x}) = \mathrm{J}_F(\bar{x})^\top \, \nabla^2 \pi(F(\bar{x})) \, \mathrm{J}_F(\bar{x}),$$

which is always negative semidefinite. With this approximation, the update in the k-th iteration is defined as

$$x^{(k+1)} = \underset{\|x - x^{(k)}\| \le \Delta}{\arg\max} \; \pi(F(x^{(k)}) + \mathrm{J}_F(x^{(k)})(x - x^{(k)})).$$

The constraint $\|x - x^{(k)}\| \le \Delta$ ensures that the next point $x^{(k+1)}$ stays within a certain neighborhood of the previous point $x^{(k)}$. This constraint guarantees the similarity between $\pi_F(x)$ and its approximation $\widehat{\pi_F}(x)$ at each iteration. Since the decoding algorithm works well empirically even without this constraint, we do not use this constraint in the main paper.

To apply Gauss-Newton optimization to our model, we first rewrite the log joint density as a composite of a smooth map and a concave function. The log joint density is the summation of the log prior for $\boldsymbol{x}$ and the point process log-likelihood:

$$\log p(\boldsymbol{x}, \boldsymbol{\kappa}) = \log p(\boldsymbol{x}) + \log p(\boldsymbol{\kappa} \mid \boldsymbol{x}).$$

The point process log-likelihood term is replaced by the lower bound derived in the main paper. Since we define the prior for $\boldsymbol{x}$ as the Gaussian distribution, the first term is already concave. Hence, we need to express the second term as a composite form.

The lower bound consists of the Rényi lower bound and χ upper bound. We rewrite these terms as composite forms, respectively. The Rényi lower bound and χ upper bound around $\bar{x}$ are expressed as

$$\frac{1}{\alpha} \log \left(\frac{1}{L} \sum_{l=1}^{L} v_l(x)^\alpha \right), \quad -\left(\frac{1}{L} \sum_{l=1}^{L} w_l(x)^\beta \right)^{\frac{1}{\beta}},$$

where $v_l(x)$ and $w_l(x)$ are importance weights defined as

$$v_l(x) = \frac{\lambda_0 \, p(x, \kappa \mid z_l) \, p(z_l)}{q(z_l \mid \bar{x}, \kappa)}, \quad w_l(x) = \frac{\lambda_0 \, p(x \mid z_l) \, p(z_l)}{q(z_l \mid \bar{x})}.$$

Here, we define the denominator of the importance weights not to depend on x, but rather on $\bar{x}$. This approximation simplifies the calculation of the gradient under our model, making it easier to apply the optimization step. If we use the Gaussian distribution for the decoder as described in the main paper, then the gradient of $\log v_l(x)$ is

$$\begin{aligned}\nabla \log v_l(x) &= \nabla_x \log p(x, \kappa \mid z_l) \\ &= -\Lambda_\theta^{\mathrm{z}\to\mathrm{x}}(z_l)\,(x - \mu_\theta^{\mathrm{z}\to\mathrm{x}}(z_l))\,.\end{aligned}$$

Thus, calculating the gradient does not require automatic differentiation tools.

We define the smooth maps F and G, along with the concave functions π and ρ, such that $\pi(F(x))$ and $\rho(G(x))$ are equal to the Rényi lower bound and χ upper bound, respectively. We define these functions as follows:

$$\pi(\boldsymbol{\zeta}) = \frac{1}{\alpha}\log\left(\frac{1}{L}\sum_{l=1}^{L}\zeta_l^\alpha\right), \qquad F(x) = \begin{bmatrix} v_1(x) \\ \vdots \\ v_L(x) \end{bmatrix}, \qquad \pi_F(x) = \frac{1}{\alpha}\log\left(\frac{1}{L}\sum_{l=1}^{L} v_l(x)^\alpha\right),$$

$$\rho(\boldsymbol{\eta}) = -\left(\frac{1}{L}\sum_{l=1}^{L}\exp(\beta\eta_l)\right)^{\frac{1}{\beta}}, \qquad G(x) = \begin{bmatrix} \log w_1(x) \\ \vdots \\ \log w_L(x) \end{bmatrix}, \qquad \rho_G(x) = -\left(\frac{1}{L}\sum_{l=1}^{L} w_l(x)^\beta\right)^{\frac{1}{\beta}}.$$

With these mappings and functions, the approximations of the Rényi lower bound and the χ upper bound around $\bar{x}$ are

$$\begin{aligned}\widehat{\pi_F}(x) &:= \pi(F(\bar{x}) + \mathrm{J}_F(\bar{x})(x - \bar{x})), \\ \widehat{\rho_G}(x) &:= \rho(G(\bar{x}) + \mathrm{J}_G(\bar{x})(x - \bar{x})).\end{aligned}$$

The gradients and the Hessian matrices of these $\widehat{\pi_F}$ and $\widehat{\rho_G}$ evaluated at $\bar{x}$ are

$$
\begin{aligned}
\nabla\widehat{\pi_F}(\bar{x}) &= \sum_{l=1}^{L}\left(\frac{v_l(\bar{x})^\alpha}{\sum_{k=1}^{L} v_k(\bar{x})^\alpha}\right)\nabla\log v_l(\bar{x}),\\
\nabla^2\widehat{\pi_F}(\bar{x}) &= -\alpha\,\nabla\widehat{\pi_F}(\bar{x})\,\nabla\widehat{\pi_F}(\bar{x})^\top - (1-\alpha)\sum_{l=1}^{L}\left(\frac{v_l(\bar{x})^\alpha}{\sum_{k=1}^{L} v_k(\bar{x})^\alpha}\right)\nabla\log v_l(\bar{x})\,\nabla\log v_l(\bar{x})^\top,\\
\nabla\widehat{\rho_G}(\bar{x}) &= -\widehat{\rho_G}(\bar{x})\sum_{l=1}^{L}\left(\frac{w_l(\bar{x})^\beta}{\sum_{k=1}^{L} w_k(\bar{x})^\beta}\right)\nabla\log w_l(\bar{x}),\\
\nabla^2\widehat{\rho_G}(\bar{x}) &= -\widehat{\rho_G}(\bar{x})\left((1-\beta)\left(\frac{\nabla\widehat{\rho_G}(\bar{x})}{\widehat{\rho_G}(\bar{x})}\right)\left(\frac{\nabla\widehat{\rho_G}(\bar{x})}{\widehat{\rho_G}(\bar{x})}\right)^\top + \beta\sum_{l=1}^{L}\left(\frac{w_l(\bar{x})^\beta}{\sum_{k=1}^{L} w_k(\bar{x})^\beta}\right)\nabla\log w_l(\bar{x})\nabla\log w_l(\bar{x})^\top\right).
\end{aligned}
$$

Both $\nabla^2\widehat{\pi_F}(\bar{x})$ and $\nabla^2\widehat{\rho_G}(\bar{x})$ are negative semidefinite, ensuring that the approximated functions are concave.

We move on to the explanation about decoding. The $v_l(x)$ and $w_l(x)$ in the previous definition actually depend on x_r, κ_{ri}, and z_{ril}. To indicate the dependency on subscripts r, i, and k, we introduce the following notations:

$$
v_{ril}(x_r) = \frac{\lambda_0\, p(x_r, \kappa_{ril} \mid z_{ril})\, p(z_{ril})}{q(z_{ril} \mid \bar{x}_r, \kappa_{ril})}, \qquad F_{ri}(x) = \begin{bmatrix} v_{ri1}(x) \\ \vdots \\ v_{riL}(x) \end{bmatrix}, \qquad \pi_{F_{ri}}(x) = \frac{1}{\alpha}\log\left(\frac{1}{L}\sum_{l=1}^{L} v_{ril}(x)^\alpha\right),
$$

$$
w_{rl}(x_r) = \frac{\lambda_0\, p(x_r \mid z_{rl})\, p(z_{rl})}{q(z_{rl} \mid \bar{x}_r)}, \qquad G_r(x) = \begin{bmatrix} \log w_{r1}(x) \\ \vdots \\ \log w_{rL}(x) \end{bmatrix}, \qquad \rho_{G_r}(x) = -\left(\frac{1}{L}\sum_{l=1}^{L} w_{rl}(x)^\beta\right)^{\frac{1}{\beta}}.
$$

Then, the lower bound of the point process log-likelihood at the r-th bin

$$
\begin{aligned}
\mathcal{L}_r(x_r) &= \sum_{i=1}^{n_r}\frac{1}{\alpha}\log\left(\frac{1}{L}\sum_{l=1}^{L} v_{ril}(x_r)^\alpha\right) - \left(\frac{1}{L}\sum_{l=1}^{L} w_{rl}(x_r)^\beta\right)^{\frac{1}{\beta}}\\
&= \sum_{i=1}^{n_r}\pi_{Fri}(x_r) + \rho_{Gr}(x_r)
\end{aligned}
$$

is approximated by

$$
\widehat{\mathcal{L}}_r(x_r) = \sum_{i=1}^{n_r}\widehat{\pi_{Fri}}(x_r) + \widehat{\rho_{Gr}}(x_r),
$$

where $\widehat{\pi_{F_{ri}}}(x_r)$ and $\widehat{\rho_{G_r}}(x_r)$ are the Gauss-Newton approximation of $\pi_{F_{ri}}(x_r)$ and $\rho_{G_r}(x_r)$, respectively.

Based on the preceding discussion, the Hessian matrix of this approximated lower bound is always negative semidefinite. This ensures that the lower bound is always concave, and the joint log density replaced with this lower bound

$$\log p(\boldsymbol{x}) + \sum_{r=1}^{R} \widehat{\mathcal{L}}_r(x_r)$$

is also concave.

Hereafter, we show the details of the Newton update. Define the following matrices:

$$C(\boldsymbol{x}) = \begin{pmatrix} -\nabla^2 \widehat{\mathcal{L}_r}(\bar{x}_1) & & \\ & \ddots & \\ & & -\nabla^2 \widehat{\mathcal{L}_r}(\bar{x}_R) \end{pmatrix}, \quad \boldsymbol{d}(\boldsymbol{x}) = \begin{pmatrix} \nabla \widehat{\mathcal{L}_r}(\bar{x}_1) \\ \vdots \\ \nabla \widehat{\mathcal{L}_r}(\bar{x}_R) \end{pmatrix},$$

$$\boldsymbol{F} = \begin{pmatrix} I & & & \\ -F & I & & \\ & \ddots & \ddots & \\ & & -F & I \end{pmatrix}, \quad \boldsymbol{V} = \begin{pmatrix} V_1 & & & \\ & V & & \\ & & \ddots & \\ & & & V \end{pmatrix}, \quad \boldsymbol{a} = \begin{pmatrix} a_1 \\ a \\ \vdots \\ a \end{pmatrix}.$$

Then, the Newton update at the k-th iteration is

$$\boldsymbol{x}^{(k+1)} \leftarrow \boldsymbol{x}^{(k)} - \left(\boldsymbol{C}(\boldsymbol{x}^{(k)}) + \boldsymbol{F}^\top \boldsymbol{V} \boldsymbol{F}\right)^{-1} \left(\boldsymbol{d}(\boldsymbol{x}^{(k)}) + \boldsymbol{F}^\top \boldsymbol{V}(\boldsymbol{F}\boldsymbol{x}^{(k)} - \boldsymbol{a})\right).$$

Here, the matrix $\boldsymbol{C}(\boldsymbol{x}^{(k)}) + \boldsymbol{F}^\top \boldsymbol{V} \boldsymbol{F}$ is a block tridiagonal matrix. Thus, using the same technique as in Section S1.6, the matrix multiplication of the inverse of this matrix incurs only $O(R)$ cost. After K iterations starting from the initial value $\boldsymbol{x}^{(0)}$, we determine the mean and precision matrix of the approximate posterior as

$$\begin{aligned} \widehat{\boldsymbol{\mu}} &\leftarrow \boldsymbol{x}^{(K)}, \\ \widehat{\boldsymbol{\Lambda}} &\leftarrow \boldsymbol{C}(\boldsymbol{x}^{(K)}) + \boldsymbol{F}^\top \boldsymbol{V} \boldsymbol{F}. \end{aligned}$$

Furthermore, we can efficiently generate samples $\{\boldsymbol{x}_s\}_{s=1}^{S}$ from the approximate posterior in $O(R)$ time.

With the generated samples, we can approximate the posterior of $\boldsymbol{y}$ as

$$p(\boldsymbol{y} \mid \boldsymbol{\kappa}) = \int p(\boldsymbol{y} \mid \boldsymbol{x})\, p(\boldsymbol{x} \mid \boldsymbol{\kappa})\, \mathrm{d}\boldsymbol{x} \approx \frac{1}{S} \sum_{s=1}^{S} p(\boldsymbol{y} \mid \boldsymbol{x}_s).$$

In the rest of this section, we discuss how to determine the initial value $\boldsymbol{x}^{(0)}$. Given that the posterior density within our model is not concave, selecting an appropriate initial value becomes crucial for achieving a good solution. To decide the initial value, we employ the Laplace recursive filter for point process observation [11]. This method is based on a forward filtering algorithm that recursively approximates the posterior using a Laplace approximation at each step.

In a general state space model, the objective of filtering is to calculate the posterior of the r-th hidden state based on observations up to time step r. In our scenario, the hidden state is represented by x_r, the observation corresponds to the unsorted spikes $\boldsymbol{\kappa}_r$, and the filtered distribution of interest is $p(x_r \mid \boldsymbol{\kappa}_{l \leq r})$. Specifically, the filtering algorithm iterates the following update formula to calculate the one-step predictive distributions and filter distributions alternately:

$$\begin{aligned} p(x_r \mid \{\boldsymbol{\kappa}_l\}_{l \leq r-1}) &= \int p(x_r \mid x_{r-1})\, p(x_{r-1} \mid \{\boldsymbol{\kappa}_l\}_{l \leq r-1})\, \mathrm{d}x_{r-1}, \\ p(x_r \mid \{\boldsymbol{\kappa}_l\}_{l \leq r}) &= \frac{p(\boldsymbol{\kappa}_r \mid x_r)\, p(x_r \mid \{\boldsymbol{\kappa}_l\}_{l \leq r-1})}{\int p(\boldsymbol{\kappa}_r \mid x_r)\, p(x_r \mid \{\boldsymbol{\kappa}_l\}_{l \leq r-1})\, \mathrm{d}x_r}. \end{aligned}$$

However, since the point process likelihood $p(\boldsymbol{\kappa}_r \mid x_r)$ is not Gaussian, the integrals in these equations are not tractable under our model. To address such issues, the Laplace filter recursively approximates the posterior $p(x_r \mid \{\boldsymbol{\kappa}_l\}_{l \leq r})$ as Gaussian by using a Laplace approximation.

Given that the $(r-1)$-th filtered distribution $p(x_{r-1} \mid \{\boldsymbol{\kappa}_l\}_{l \leq r-1})$ and the transition distribution $p(x_r \mid x_{r-1})$ are both Gaussian, the one-step prediction distribution $p(x_r \mid \{\boldsymbol{\kappa}_l\}_{l \leq r-1})$ also follows a Gaussian distribution. Consequently, updates for the one-step prediction distribution and the filtered distribution simplify to updates of the mean and the precision matrix of a Gaussian distribution. More specifically, the Laplace filter iterates the following update formulas for $r = 1, \ldots, R$ to calculate one-step predictive distributions and filter distributions:

- Update for one-step predictive distribution:

$$p(x_r \mid \{\boldsymbol{\kappa}_l\}_{l\le r-1}) = \mathrm{N}\left(x_r \,\middle|\, \widehat{\mu}_{r|r-1}, \widehat{\Lambda}_{r|r-1}\right),$$

$$\widehat{\mu}_{r|r-1} = F\widehat{\mu}_{r-1|r-1} + a,$$

$$\widehat{\Lambda}_{r|r-1} = \left(F\widehat{\Lambda}^{-1}_{r-1|r-1}F^\top + V\right)^{-1}.$$

- Update for filtered distribution:

$$p(x_r \mid \{\boldsymbol{\kappa}_l\}_{l\le r}) = \mathrm{N}\left(x_r \,\middle|\, \widehat{\mu}_{r|r}, \widehat{\Lambda}_{r|r}\right),$$

$$\widehat{\mu}_{r|r} = \arg\max_{x_r} \left[\log p(\boldsymbol{\kappa}_r \mid x_r) + \log p(x_r \mid \{\boldsymbol{\kappa}_l\}_{l\le r-1})\right],$$

$$\widehat{\Lambda}_{r|r} = -\nabla^2_{x_r} \left[\log p(\boldsymbol{\kappa}_r \mid x_r) + \log p(x_r \mid \{\boldsymbol{\kappa}_l\}_{l\le r-1})\right]_{x_r=\widehat{\mu}_{r|r}}.$$

To calculate the filtered mean $\widehat{\mu}_{r|r}$ and the filtered precision matrix $\widehat{\Lambda}_{r|r}$, we maximize the summation of the point process log-likelihood and the log density of the one-step predictive distribution. However, the point process log-likelihood is not guaranteed to be concave, giving rise to the same problems mentioned in the previous discussion. To overcome these problems, we use the Gauss-Newton approximation again. We replace the point process log-likelihood by its Gauss-Newton approximation as

$$\widehat{\mu}_{r|r} = \arg\max_{x_r} \left[\widehat{\mathcal{L}}_r(x_r) + \log p(x_r \mid \{\boldsymbol{\kappa}_l\}_{l\le r-1})\right],$$

$$\widehat{\Lambda}_{r|r} = -\nabla^2\widehat{\mathcal{L}}_r(\widehat{\mu}_{r|r}) - \nabla^2_{x_r} \log p(x_r \mid \{\boldsymbol{\kappa}_l\}_{l\le r-1})\big|_{x_r=\widehat{\mu}_{r|r}}.$$

With this approximation, the Newton update is

$$x_r^{(k+1)} \leftarrow x_r^{(k)} - \left(-\nabla^2\widehat{\mathcal{L}}_r(x_r^{(k)}) + \widehat{\Lambda}_{r|r-1}\right)^{-1}\left(\nabla\widehat{\mathcal{L}}_r(x_r^{(k)}) + \widehat{\Lambda}_{r|r-1}(x_r^{(k)} - \widehat{\mu}_{r|r-1})\right).$$

After K iterations starting from the initial value $x_r^{(0)} = \widehat{\mu}_{r|r-1}$, the mean and precision matrix of the filtered distribution are updated as

$$\widehat{\mu}_{r|r} \leftarrow x_r^{(K)},$$

$$\widehat{\Lambda}_{r|r} \leftarrow -\nabla^2\widehat{\mathcal{L}}_r(x_r^{(K)}) + \widehat{\Lambda}_{r|r-1}.$$

Table B. Summary of models.

Model	Type	Prediction task	Decoding task
JVAE	Unsorted	✓	✓
CVAE	Unsorted	✓	✓
GMM	Unsorted	✓	✓
RMPP	Unsorted	✓	×
GRU	Sorted	×	✓
DNN	Sorted	×	✓
WF	Sorted	×	✓

Once the filtered distributions have been calculated, we apply the Kalman backward smoother to obtain the smoothed distributions $p(x_r \mid \{\boldsymbol{\kappa}_r\}_{r=1}^R) = \mathrm{N}(x_r \mid \widehat{\mu}_{r|R}, \widehat{\Lambda}_{r|R})$ using the following equations:

$$\widehat{\mu}_{r|R} = \widehat{\mu}_{r|r} + \widehat{\Lambda}_{r|r}^{-1} F^\top \widehat{\Lambda}_{r+1|r} \left(\widehat{\mu}_{r+1|R} - \widehat{\mu}_{r+1|r}\right),$$
$$\widehat{\Lambda}_{r|R} = \left(\widehat{\Lambda}_{r|r}^{-1} + \widehat{\Lambda}_{r|r}^{-1} F^\top \widehat{\Lambda}_{r+1|r} \left(\widehat{\Lambda}_{r+1|R}^{-1} - \widehat{\Lambda}_{r+1|r}^{-1}\right) \widehat{\Lambda}_{r+1|r} F \widehat{\Lambda}_{r|r}^{-1}\right)^{-1}.$$

Finally, the initial value $\boldsymbol{x}^{(0)}$ for the direct optimization method is given by the smoothed mean:

$$\boldsymbol{x}^{(0)} \leftarrow \begin{bmatrix} \widehat{\mu}_{1|R} \\ \vdots \\ \widehat{\mu}_{R|R} \end{bmatrix}.$$

S2 Details of experiments

S2.1 Compared models

In the main paper, we compare our model with existing models based on the prediction task and the decoding task. In this section, we provide detailed explanations of these models. For the sake of completeness, we reiterate the same explanations from the main paper.

All models utilizing marked point processes share the same state space model. The distinction among these models lies in how they define the point process likelihood. On the other hand, the models employed for sorted decoding do not use the state space model.

Some models cannot be applied to the prediction task or the decoding task. Table B provides a summary of the available tasks for each model.

CVAE The joint mark intensity can be decomposed into the product of the ground intensity and the conditional density of the mark given the state:

$$\lambda(x, \kappa) = \lambda(x)\, p(\kappa \mid x).$$

Based on this decomposition, the point process log-likelihood is expressed as the summation of two parts:

$$\sum_{r=1}^{R} \sum_{i=1}^{n_r} \log p(\kappa_{ri} \mid x_r) \quad + \quad \sum_{r=1}^{R} \left[n_r \log \lambda(x_r) - \lambda(x_r) \right].$$

The first part is the conditional log-likelihood of the mark given the state, while the second part is the point process log-likelihood without marks. Existing marked point process models often model the first part as a parametric distribution whose parameters depend on the history of the process, and the second part as a deterministic function also dependent on the history. Such models are inadequate when the actual mark distribution deviates from simple parametric distributions.

CVAE employs a deterministic neural network for the ground intensity and a conditional variational autoencoder for the mark distribution. The conditional variational autoencoder is an extension of VAE designed for conditional density estimation. It represents the conditional density as

$$p(\kappa \mid x) = \int p(\kappa \mid x, z)\, p(z \mid x)\, \mathrm{d}z.$$

In this equation, $p(\kappa \mid x, z)$ is the decoder, and $p(z \mid x)$ is a prior depending on x. These distributions are often defined as exponential families whose parameters are neural networks. To estimate parameters, we maximize the log-likelihood $\log p(\kappa \mid x)$. Since it contains the integral over z, we use an encoder $q(z \mid x, \kappa)$ to obtain the lower bound and maximize this lower bound instead. Similarly to our model, the Rényi lower bound is defined as

$$\log p(\kappa \mid x) \geq \frac{1}{\alpha} \log \left(\mathrm{E}_{q(z|x,\kappa)} \left[\left(\frac{p(\kappa \mid x, z)\, p(z \mid x)}{q(z \mid x, \kappa)} \right)^{\alpha} \right] \right).$$

To perform optimization, the expectations are replaced with a Monte Carlo approximation as

$$\frac{1}{\alpha} \log \left(\frac{1}{L} \sum_{l=1}^{L} \left(\frac{p(\kappa \mid x, z_l)\, p(z_l \mid x)}{q(z_l \mid x, \kappa)} \right)^{\alpha} \right),$$

where $\{z_l\}_{l=1}^{L}$ are i.i.d. samples from the decoder $q(z \mid x, \kappa)$. Similarly to our model, the gradients of the

Rényi lower bound are calculated using doubly reparameterized gradients in the experiments.

The specific choices for the decoder, prior and encoder are Gaussian distributions whose means and precision matrices are neural networks:

$$p(\kappa \mid x, z) = \mathrm{N}(\kappa \mid \mu_\theta^{\mathsf{xz}\to\mathsf{\kappa}}(x, z), \Lambda_\theta^{\mathsf{xz}\to\mathsf{\kappa}}(x, z)),$$
$$p(z \mid x) = \mathrm{N}(z \mid \mu_\theta^{\mathsf{x}\to\mathsf{z}}(x), \Lambda_\theta^{\mathsf{x}\to\mathsf{z}}(x)),$$
$$q(z \mid x, \kappa) = \mathrm{N}(z \mid \nu_\phi^{\mathsf{x\kappa}\to\mathsf{z}}(x, \kappa), \Xi_\phi^{\mathsf{x\kappa}\to\mathsf{z}}(x, \kappa)).$$

Next, we provide an explanation about the decoding. Similarly to our model, the lower bound of the point process log-likelihood is not concave with respect to x. Thus, we use the Gauss-Newton approximation to the lower bound. The lower bound consists of the following terms:

$$\frac{1}{\alpha} \log \left(\frac{1}{L} \sum_{l=1}^{L} v_l(x)^\alpha \right), \quad -\lambda(x),$$

where $v_l(x)$ is the importance weight defined as

$$v_l(x) = \frac{\lambda(x)\, p(\kappa \mid x, z_l)\, p(z_l \mid x)}{q(z_l \mid \bar{x}, \kappa)}.$$

The Rényi lower bound term is the same as the one in our model. Thus, we can use the same map and function to rewrite this term as a composite form. The main difference lies in the second term, which is not guaranteed to be concave. To ensure concavity, we rewrite these terms in a concave composite form as follows:

$$\pi(\boldsymbol{\zeta}) = \frac{1}{\alpha} \log \left(\frac{1}{L} \sum_{l=1}^{L} \zeta_l^\alpha \right), \qquad F(x) = \begin{bmatrix} v_1(x) \\ \vdots \\ v_L(x) \end{bmatrix}, \qquad \pi_F(x) = \frac{1}{\alpha} \log \left(\frac{1}{L} \sum_{l=1}^{L} v_l(x)^\alpha \right),$$
$$\rho(\boldsymbol{\eta}) = -\exp(\eta), \qquad G(x) = \log \lambda(x), \qquad \rho_G(x) = -\lambda(x).$$

With these functions and mappings, we approximate $\pi_F(x)$ and $\rho_G(x)$ with $\widehat{\pi}_F(x)$ and $\widehat{\rho}_G(x)$, respectively, in a similar manner. By combining these approximations, we obtain a concave approximation of the lower bound. The remaining step is the same as that described in our model.

GMM GMM defines the joint mark intensity as a Gaussian mixture model multiplied by a scale parameter:

$$\lambda(x, \kappa) = \sum_{l=1}^{L} \lambda_l \, \mathrm{N}(x \mid \mu_l^{\mathrm{x}}, \Sigma_l^{\mathrm{x}}) \, \mathrm{N}(\kappa \mid \mu_l^{\kappa}, \Sigma_l^{\kappa}).$$

The strength of this model is that the parameters are interpretable: $\lambda_l \, \mathrm{N}(x \mid \mu_l^{\mathrm{x}}, \Sigma_l^{\mathrm{x}})$ is the tuning curve of the l-th neuron, and $\mathrm{N}(\kappa \mid \mu_l^{\kappa}, \Sigma_l^{\kappa})$ is the spike waveform distribution of this neuron. The work by [12] expressed the joint mark intensity as a Gaussian mixture and introduced dynamics to its parameters for handling neuronal plasticity.

With this definition, the integral with respect to κ also reduces to a mixture as

$$\int \lambda(x, \kappa) \, \mathrm{d}\kappa = \sum_{l=1}^{L} \lambda_l \, \mathrm{N}(x \mid \mu_l^{\mathrm{x}}, \Sigma_l^{\mathrm{x}}).$$

Thus, the point process log-likelihood under this model is tractable. We estimate the parameters by maximizing this log-likelihood.

Next, we provide an explanation of the decoding process. As with the preceding two models, the log-likelihood is not concave with respect to x. Thus, we rewrite it as a concave composite form. Define the importance weights as

$$v_l(x) = L \, \lambda_l \, \mathrm{N}(x \mid \mu_l^{\mathrm{x}}, \Lambda_l^{\mathrm{x}}) \, \mathrm{N}(\kappa \mid \mu_l^{\mathrm{x}}, \Lambda_l^{\kappa}), \quad w_l(x) = L \, \lambda_l \, \mathrm{N}(x \mid \mu_l^{\mathrm{x}}, \Lambda_l^{\mathrm{x}}).$$

Then, the point process log-likelihood consists of the following terms:

$$\pi_F(x) = \log \left(\frac{1}{L} \sum_{l=1}^{L} v_l(x) \right), \quad \rho_G(x) = -\frac{1}{L} \sum_{l=1}^{L} w_l(x),$$

which is equal to our model when $\alpha = 1$ and $\beta = 1$. Thus, we can use the same technique to approximate these terms. The remaining decoding steps are the same as the preceding two models.

RMPP RMPP is based on recurrent marked point processes [13]. The recurrent marked point process assumes that the i-th event (t_i, κ_i) depends on the history of previous events $\{(t_j, \kappa_j)\}_{t_j < t_i}$, and captures this dependency with a recurrent neural network. Specifically, the latent state z_i is assigned to each event (t_i, κ_i), and the evolution of this state is modeled by a recurrent neural network.

We assume that only the mark distribution at time t depends on the previous observations as

$$\lambda(t, x, \kappa \mid \{(t_i, x_i, \kappa_i)\}_{t_i<t}) = \lambda(x)\, p(\kappa \mid \{(t_i, x_i, \kappa_i)\}_{t_i<t})$$

and use a Gated Recurrent Unit to model the latent history of the process:

$$z_i = \mathrm{GRU}(z_{i-1}, \Delta t_{i-1}, x_{i-1}, \kappa_{i-1}),$$

where $\Delta t_i = t_i - t_{i-1}$. Given the latent states $\{z_i\}$, we model the mark distribution at the i^*-th spike time as a Gaussian mixture model:

$$p(\kappa \mid \{(t_i, x_i, \kappa_i)\}_{t_i<t_{i^*}}) = \sum_{l=1}^{L} \pi_l(z_{i^*})\, \mathrm{N}(\kappa \mid \mu_l^{\mathrm{z}\to\kappa}(z_{i^*}), \Lambda_l^{\mathrm{z}\to\kappa}(z_{i^*})).$$

The existing recurrent marked point process often defines the ground intensity as dependent on the latent state. We attempted to use this definition, but it did not perform well in our experiments. Thus, we defined the ground intensity as a deterministic function that is only dependent on x.

We estimate parameters by maximum likelihood estimation.

GRU, DNN, WF To compare the performance of our model with past sorted decoding, we also applied the regression-based methods implemented in [14]. This implementation contains several regression methods that receive the binned spikes and output the predicted covariate.

To create binned spikes, we perform the following steps. First, we apply spike sorting to mark sequences $\{\kappa_i\}_{i=1}^{n}$. In this step, we assume that the marks of the spikes are distributed according to a Gaussian mixture and then estimate their parameters using maximum likelihood estimation. Next, based on the estimated parameters, we divide the spikes into clusters $\{t_{ci}\}_{i=1}^{n_c}$, $c = 1, \ldots, C$, where c denotes the label of the cluster and C is the number of clusters. Finally, we group the spikes into time bins of length Δt, representing the binned spike sequences as the counts of spikes $\{m_{cr}\}_{r=1}^{R}$, $c = 1, \ldots, C$.

The regression decoders implemented by [14] take the spike counts in successive bins

$$\boldsymbol{m}_r = \begin{bmatrix} m_{1,r-w_b} & \cdots & m_{C,r-w_b} \\ \vdots & & \vdots \\ m_{1,r} & \cdots & m_{C,r} \\ \vdots & & \vdots \\ m_{1,r+w_a} & \cdots & m_{C,r+w_a} \end{bmatrix}$$

as input and return the predicted covariate $\hat{y}_r$. Here, w_b and w_a are the number of bins before and after the current bin, respectively. We applied three regression decoders, named GRU, DNN, and WF. GRU is a recurrent neural network with a gating mechanism, DNN is a feedforward neural network, and WF is a Wiener filter that uses linear regression.

The decoder parameters are estimated by minimizing the mean squared error between the true covariate and the reconstructed covariate. Additionally, their code repository offers Bayesian optimization for model selection. We utilized it to select hyperparameters.

S2.2 Computer resources

In the experiments, we used a machine with an Intel Core i7-9700KF @ 3.60 GHz CPU, 48 GB of RAM, and an Nvidia RTX3090 GPU. We implemented our model and the compared models in TensorFlow.

The runtime of the model is proportional to both the number of spikes and the number of Monte Carlo samples. For example, with a total of 170,000 spikes and 10 Monte Carlo samples, training our model for 1,000 epochs took roughly 30 minutes. The Gaussian Mixture Model (GMM) took 15 minutes for the same data. Decoding with either our model or the GMM takes less than a minute.

S2.3 Synthetic data

Data generation The hidden state x_t was a Lorenz attractor defined on $\mathbb{R}^3$ with parameters $\alpha = 10$, $\beta = 28$, and $\gamma = 2.667$. Given ten different initial conditions, ten solutions for $T = 100$ seconds were calculated using the Runge-Kutta method with an interval length of $\Delta t = 0.01$. The joint mark intensity used for data generation was

$$\sum_{l=1}^{L} \lambda_l \exp\left(\left(\frac{t}{T}c_l + \left(1 - \frac{t}{T}\right) d_l\right)^{\top} x\right) \int \mathrm{N}(\kappa \mid \mu^{\mathrm{z}\to\kappa}(z), \Lambda^{\mathrm{z}\to\kappa}(z))\, \mathrm{N}(z \mid \mu_l^{\mathrm{z}}, \Lambda_l^{\mathrm{z}})\, \mathrm{d}z. \tag{S6}$$

The number of components was $L = 16$. For the favorable directions, we first selected $2L$ points on the Lorenz attractor and normalized them to unit norm. Then, we selected L pairs among these points and defined c_l and d_l as the first and second points in each pair. For the mark distribution, we prepared a variational autoencoder with a three-dimensional latent variable z:

$$p(\kappa) = \int p(\kappa \mid z)\, p(z)\, \mathrm{d}z,$$

$$p(\kappa \mid z) = \mathrm{N}(\kappa \mid \mu^{\mathrm{z}\to\kappa}(z), \Lambda^{\mathrm{z}\to\kappa}(z)), \quad p(z) = \mathrm{N}(z \mid 0, I), \quad q(z \mid \kappa) = \mathrm{N}(z \mid \nu^{\kappa\to\mathrm{z}}(\kappa), \Xi^{\kappa\to\mathrm{z}}(\kappa)).$$

The decoder and encoder consisted of four-layer fully connected neural networks with 100 tanh units in each layer, and linear output layers for mean vectors and diagonal precision matrices. We estimated the parameters of this VAE so that it generated extracellular waveforms with 32 time steps. The waveform data used for model learning were extracted from the dataset [15]. Then, we used the decoder networks $\mu^{\mathrm{z}\to\kappa}(z), \Lambda^{\mathrm{z}\to\kappa}(z)$ for the definition of the joint mark intensity (S6). For the prior term, the mean vectors μ_l^{κ} were defined as $(c_l + d_l)/2$ and the precision matrices were diagonal matrices with diagonal values of 25. The scale parameter λ_l of each component was adjusted so that the number of spikes in the whole interval was around 2,000.

Under this definition and given the hidden state path, unsorted spikes for L components were generated using a thinning algorithm [16]. Subsequently, the generated data were divided into four distinct groups, with each group of spikes assumed to originate from different probes. We repeated this procedure ten times while altering the hidden state path to produce ten datasets.

Estimation and evaluation For the lower bound maximization, we used the Adam optimizer with a learning rate of 0.001. The training dataset was split into 80 batches along the time axis, and the parameters of the state space model and the joint mark intensity model were updated by applying the gradients calculated from each batch individually. Using all batches to update the parameters was considered one epoch. In each epoch, the order of the batches was randomly shuffled. Every 50 epochs, we calculated the negative log-likelihood (NLL) for the validation data. After 1,000 epochs, we selected the parameters that minimized this NLL and used them for the evaluation tasks.

To compare the prediction performance, we calculated the NLL score for the test data. We also reconstructed the covariate and calculated the mean squared error (MSE) between the true covariate and the reconstructed covariate. In decoding, we first applied the Laplace recursive filter with five iterations at each step to obtain the initial approximate posterior and then updated it 100 times via the Newton update. After

calculating the posterior of the hidden state $\boldsymbol{x}$, we generated 100 samples from this posterior and calculated the mean and standard deviation of the reconstructed covariate $\hat{\boldsymbol{y}}$.

State space model The hidden state x_r was defined as a ten-dimensional vector. For the decoders and encoders, we adopted four-layer fully connected neural networks with 50 Leaky ReLU units in each layer for the hidden layers and linear output layers for the mean vectors. For the precision matrices, we utilized the modified Cholesky decomposition for the full precision matrix (e.g., $\Lambda = LDL^{\top}$) and adopted linear output layers for the log diagonal values of the diagonal matrix and the off-diagonal values of the lower triangular matrix.

JVAE The decoder $p(x, \kappa \mid z)$ and the encoders $q(z \mid x, \kappa)$, $q(z \mid x)$ were Gaussian distributions whose parameters were neural networks. We adopted four-layer fully connected neural networks with 50 Leaky ReLU units in each layer for the hidden layers and linear output layers for the mean vectors and precision matrices in the same manner as the state space model. For the encoder $q(z \mid x, \kappa)$, we concatenated x and κ into one vector and used it as input. The latent variable z was a ten-dimensional vector, and the prior $p(z)$ was a standard Gaussian distribution. We used $\alpha = 1$ and $\beta = 2$ for the lower bound of the point process log-likelihood and $L = 10$ for the Monte Carlo approximation. For the χ upper bound, we used the second-order jackknife estimator. The number of Monte Carlo samples used for evaluation was $L = 100$.

CVAE We chose the neural networks for the decoder and encoder in the same manner as our model. For the ground intensity $\lambda(x)$, we adopted a four-layer fully connected neural network with 50 Leaky ReLU units in each layer for the hidden layers and an exponential layer for the output. The latent variable z was a ten-dimensional vector. We used $\alpha = 1$ for the lower bound of the point process log-likelihood and $L = 10$ for the Monte Carlo approximation. For decoding, we calculated the gradients and Hessian matrices of $\log \lambda(x, \kappa)$ and $\lambda(x)$ with respect to x by using automatic differentiation. The number of Monte Carlo samples used for evaluation was $L = 100$.

GMM We selected the number of mixtures from 10, 50, and 100 that minimized the NLL for the validation data.

RMPP The latent state z was a ten-dimensional vector. The recurrent neural network for the latent state was a four-layer GRU with 50 hidden units. To prevent overfitting, we applied dropout after every GRU layer with a dropout rate of 0.5. For the parameters of the Gaussian mixture, we adopted a four-layer fully

connected neural network with 50 Leaky ReLU units in each layer for the hidden layers and linear output layers for the mean vectors, precision matrices, and mixture weights.

For the ground intensity $\lambda(x)$, we adopted the same neural network used in CVAE.

GRU, DNN, WF For spike sorting, we assumed that the spike waveform was distributed according to a Gaussian mixture. We estimated the parameters by changing the number of clusters from 1 to 20 and selected the number of clusters using the Bayesian information criterion (BIC). The number of bins before and after the current bin was set to $w_b = 9$ and $w_a = 10$. Since the length of the bin was 0.025 seconds, the total length of the window used as input was 0.5 seconds.

For GRU and DNN, we used Bayesian optimization to find the best hyperparameters. The hyperparameters included the number of units (from 50 to 200), the dropout rate (from 0 to 0.5), and the number of training epochs (from 2 to 16).

S2.4 Unsorted place cell spiking activities

Data description We chose four trials `Gatsby_08282013`, `Gatsby_08022013`, `Achilles_11012013`, and `Achilles_10252013` from the open dataset [17, 15]. These datasets contain the raw waveforms of spikes and rats' behavioral data. We extracted spikes in 400-second intervals from each trial and removed spikes that were judged as electrical noise. Table C shows the number of spikes, the dimensions of the spike features, and the number of clusters identified through spike sorting for each trial. The intervals were further subdivided into bins with a length of 0.025 seconds to apply the state space model.

Some of the probes were contaminated by noise. Thus, we excluded unsorted spikes measured at such probes.

Estimation and evaluation The model definitions, hyperparameters, model training, and evaluation were the same as those described in Section S2.3.

S3 Results of sorted decoding

In this section, we show the decoding results of the sorted decoding methods. Fig B shows the results for the synthetic data, and Fig C shows the results for the place cell data.

Fig D shows the decoding results of the Naive Bayes decoder implemented in [14]. The Naive Bayes decoder requires a grid of bins over the covariate space. Since the rat trajectories are limited to a specific

Table C. Dataset information used in the experiments. (A) Number of spikes. (B) Dimensions of spike features. For example, in `Achilles_11012013`, there are 12 probes, and each spike feature at a probe is represented by a 320-dimensional vector. (C) Number of clusters identified through spike sorting. For example, in `Achilles_10252013`, 19 clusters were detected for the first probe.

(A)

Gatsby_08282013	Gatsby_08022013	Achilles_11012013	Achilles_10252013
12328	7746	31938	55178
38376	26414	28208	23903
5505	42891	45716	12863
16165	15205	31333	24249
35537	19122	31563	29519
52239	5083	70343	55151
41942	6834	58749	47428
19643	7172	37416	18337
2790	20146	58506	21723
	13294	80948	42172
	5599	61072	32447
	44325	63673	28116
	7566		

(B)

Gatsby_08282013	Gatsby_08022013	Achilles_11012013	Achilles_10252013
256	256	320	320
256	256	320	320
256	256	320	320
224	256	320	320
192	256	320	320
256	224	320	320
256	256	320	320
256	256	320	320
256	256	320	320
	256	320	320
	256	320	320
	256	320	128
	256		

(C)

Gatsby_08282013	Gatsby_08022013	Achilles_11012013	Achilles_10252013
1	2	5	19
11	8	2	6
1	18	13	3
2	2	7	12
15	2	14	18
20	2	16	15
8	2	8	13
2	3	7	15
2	8	13	7
	3	17	11
	2	18	7
	17	9	5
	1		

part of the space (e.g., circle track), preparing appropriate grids for this decoder is challenging. This may lead to a large deviation of the decoded values from the true values.

S4 Comparison of decoding using low-dimensional and high-dimensional spike features

The existing unsorted decoding model, such as the Gaussian mixture model, cannot handle high-dimensional spike features. To demonstrate this, we compared the decoding results derived from high-dimensional spike features with those derived from low-dimensional spike features.

We compare three models. JVAE_H and GMM_H denote our model and the Gaussian mixture model applied to the unsorted spikes with raw waveforms, respectively. GMM_L denotes the GMM model applied to low-dimensional spike features. For the low-dimensional spike features, we used the three principal components of the raw waveforms. The number of mixtures for GMM was 50.

Fig E shows the results for the synthetic data, and Fig F shows the results for the place cell data. GMM_H outperformed GMM_L for the synthetic data, while GMM_L performed better than GMM_H for the place cell data. This result was influenced by the dimension of the spike feature. The dimension of the spike feature for the synthetic data was 32, significantly lower than the dimension of the spike feature for the place cell data (ranging from 128 to 320). This indicates that the Gaussian mixture model is not sufficient in the high-dimensional case.

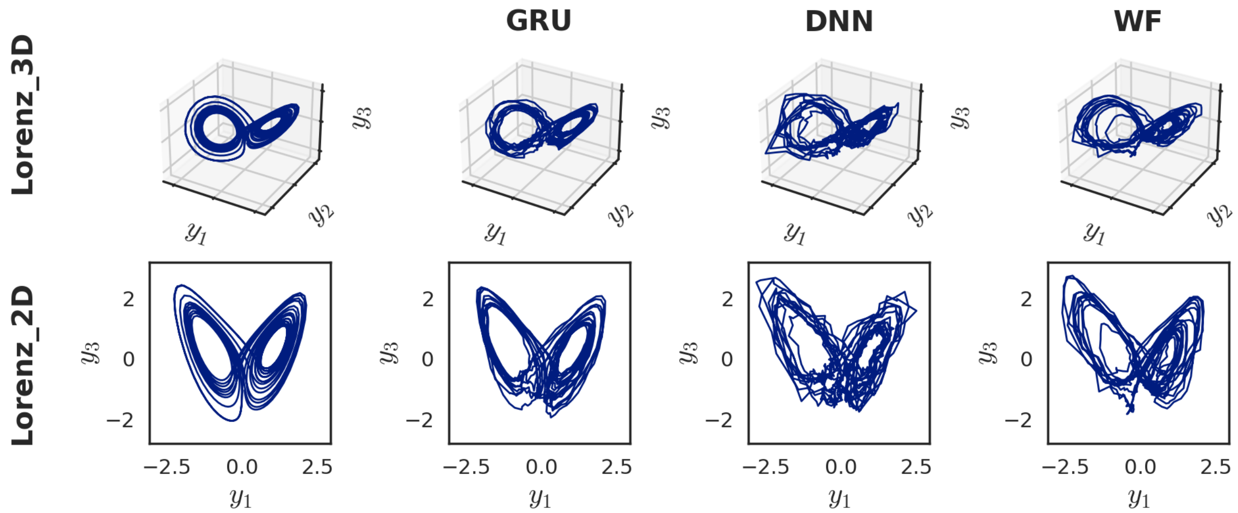

Fig B. Decoding result for the synthetic data. Each row shows the results of each trial. (1st column) True trajectories. (2nd to 4th columns) Reconstructed trajectories.

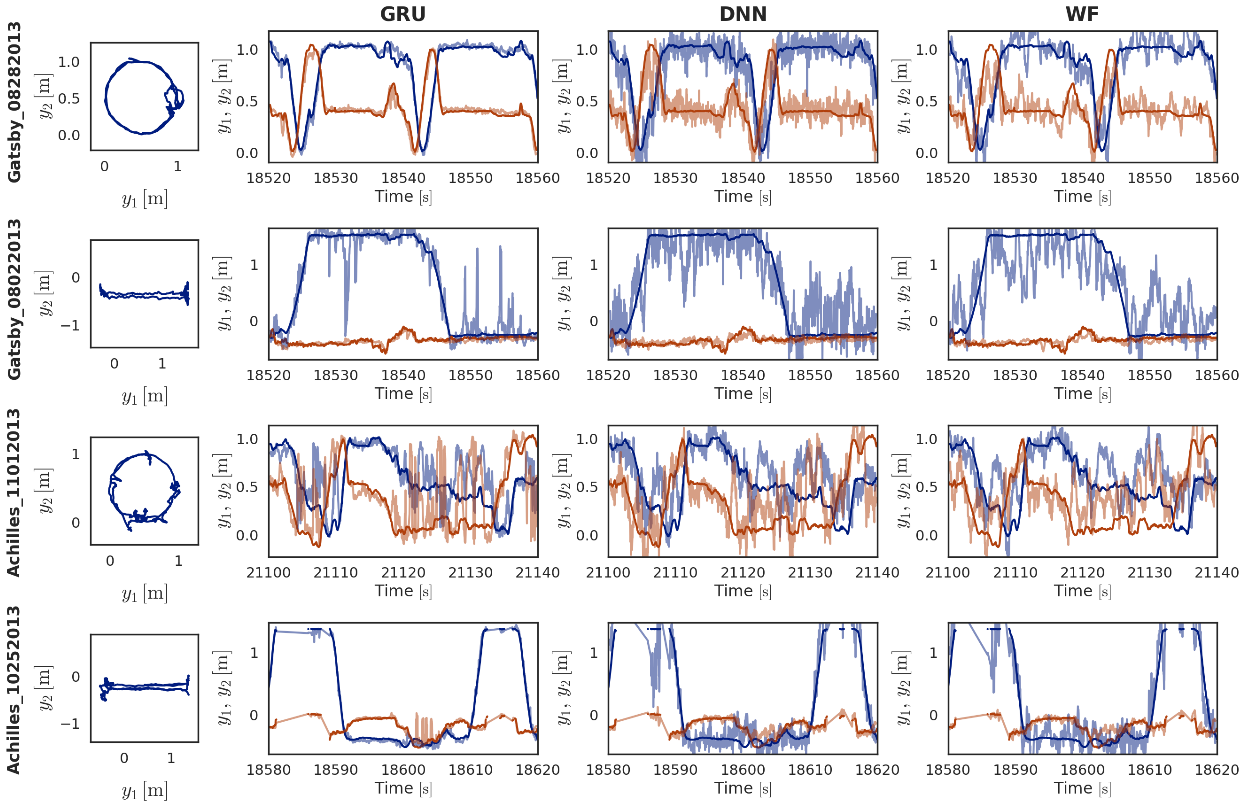

Fig C. Decoding result for the rat place cell spiking activities. Each row shows the results of each trial. (1st column) 2D plots of the rat trajectories in the circular or linear track. (2nd to 4th columns) True and reconstructed rat trajectories. Solid blue and red lines show the coordinates y_1 and y_2 of the actual rat trajectories during the experiment. Intervals without solid lines indicate that the rat trajectories were missing during those intervals due to device issues. The blue and red dashed lines represent the predicted values.

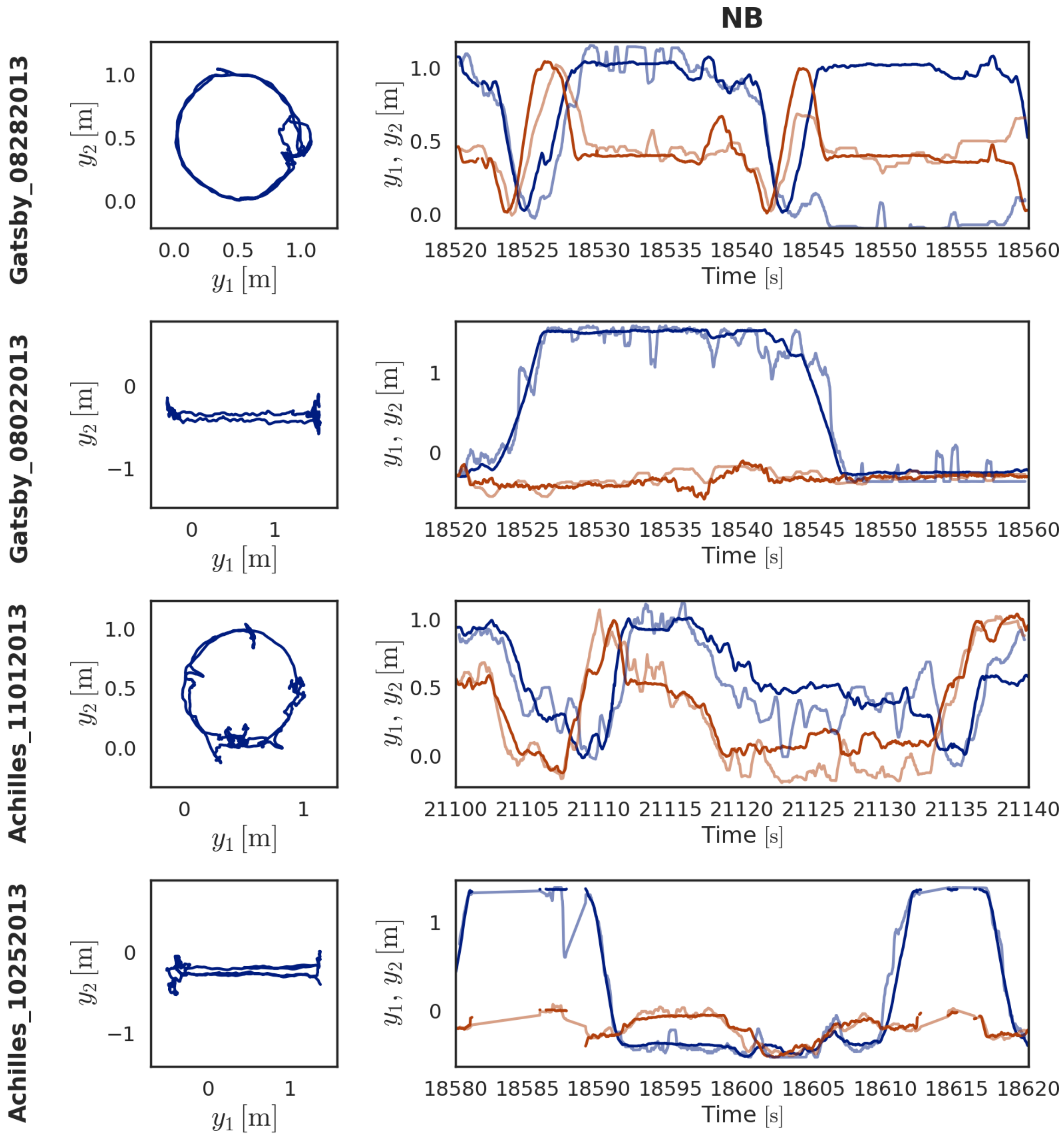

Fig D. Decoding results for the rat place cell spiking activities using the Naive Bayes decoder. The covariate space was divided into 100 × 100 bins, and the posterior probability for each bin at each time step was calculated. The decoded value was the bin with the highest posterior probability.

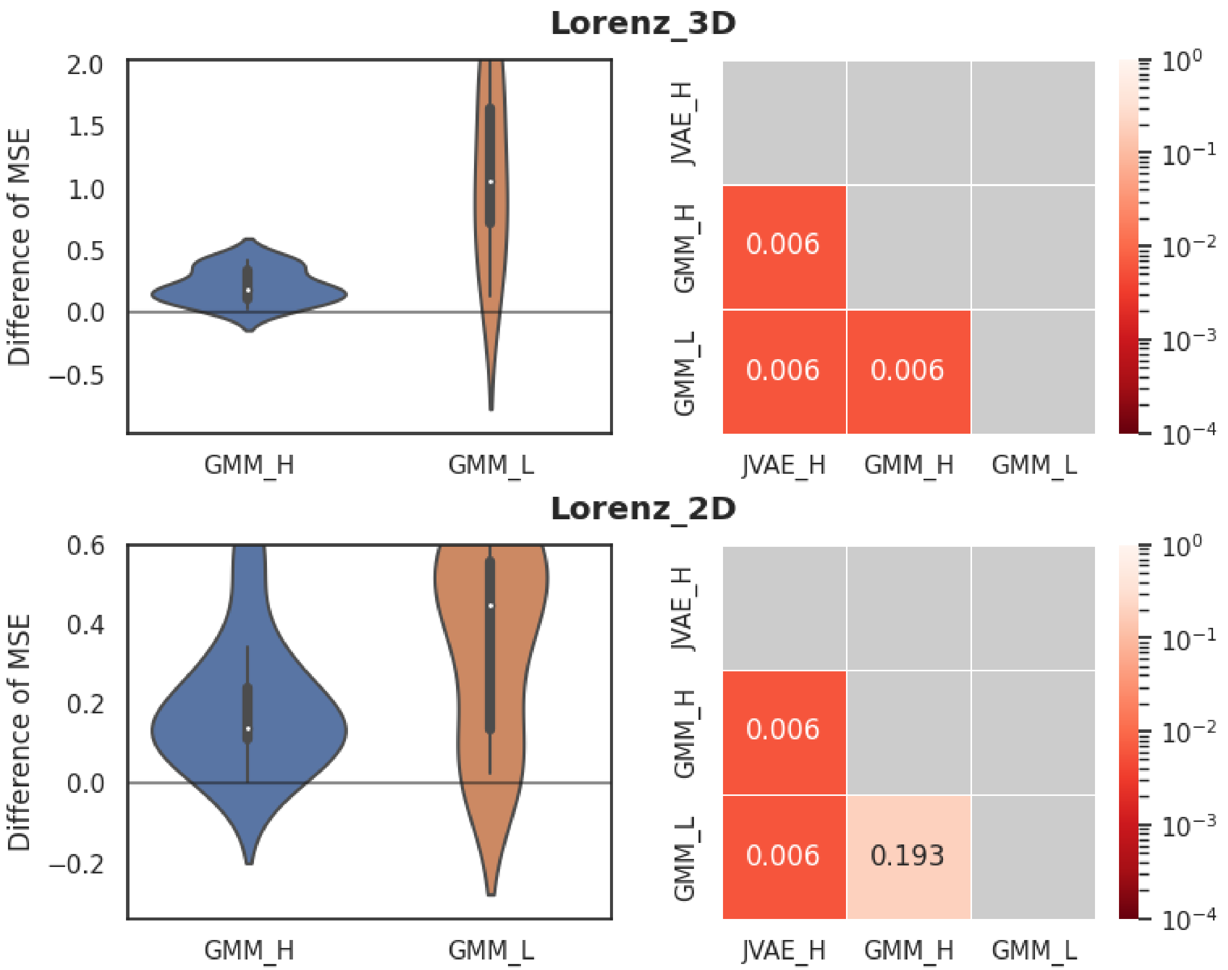

Fig E. Decoding performance for synthetic data. The first row shows the results from `Lorenz_3D`, and the second shows the results from `Lorenz_2D`. (Left) Violin plots of MSE. These values are subtracted by the baseline MSE of JVAE_H (our model). The white dots represent the median, the thick black bars indicate the interquartile range, and the thin black bars show the range from the minimum to the maximum values. (Right) The p values in the Wilcoxon test for assessing differences between two models. Each cell indicates the results for a pairing of two models. These p values are corrected by the Holm-Sidak method. The color indicates levels of the p values.

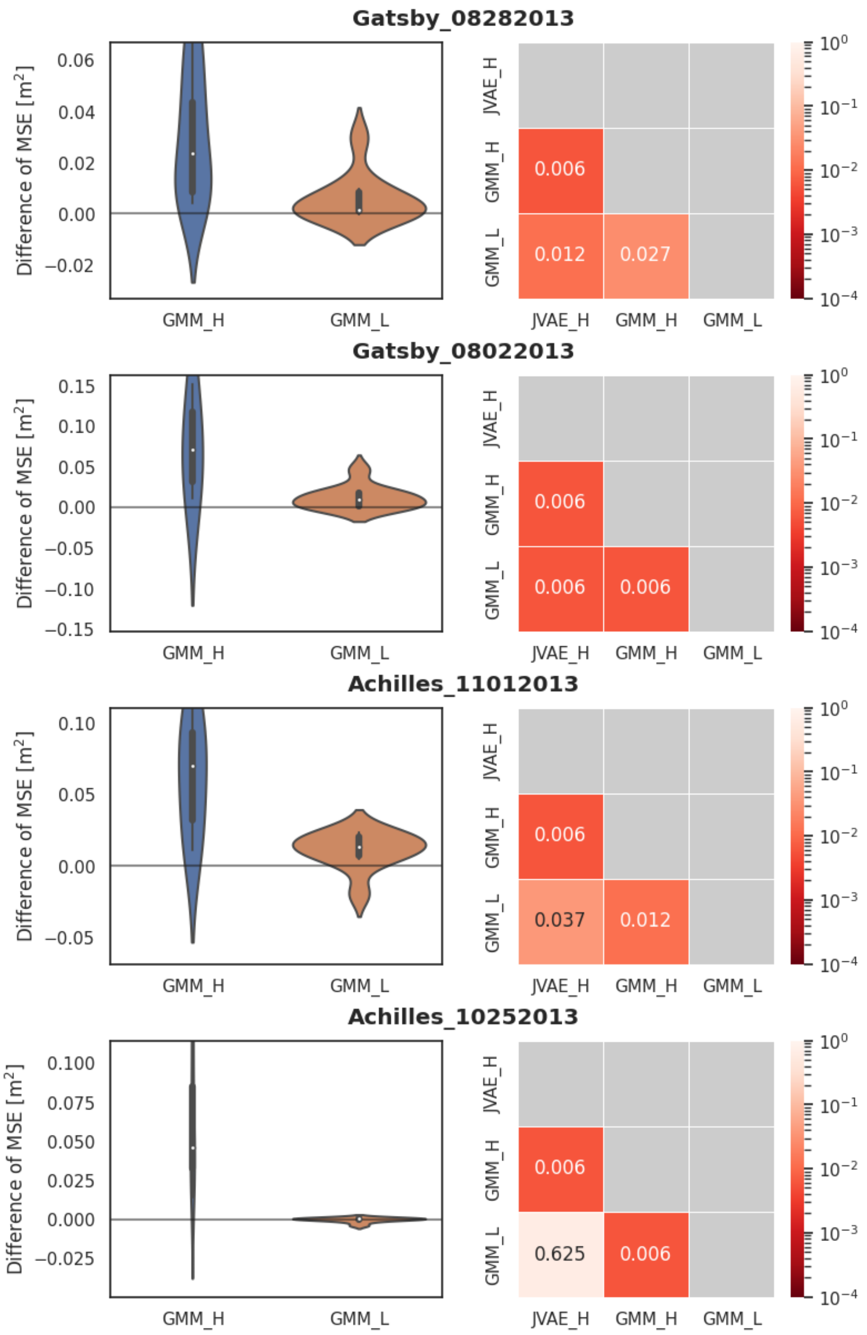

Fig F. Decoding performance for the rat place cell spiking activities. Each row shows the result of each trial. (Left) Violin plots of MSE. These values are subtracted by the baseline MSE of JVAE_H (our model). The white dots represent the median, the thick black bars indicate the interquartile range, and the thin black bars show the range from the minimum to the maximum values. (Right) The p values in the Wilcoxon test for assessing differences between two models. Each cell indicates the results for a pairing of two models. These p values are corrected by the Holm-Sidak method. The color indicates levels of the p values.

References

1. Wan N, Li D, Hovakimyan N. f-divergence variational inference. In: Adv Neural Inf Process Syst; 2020.

2. Li Y, Turner RE. Rényi divergence variational inference. In: Adv Neural Inf Process Syst; 2016.

3. Sason I. On f-divergences: integral representations, local behavior, and inequalities. Entropy. 2018;20(5).

4. Quenouille MH. Notes on bias in estimation. Biometrika. 1956;43(3/4):353–360.

5. Nowozin S. Debiasing evidence approximations: on importance-weighted autoencoders and jackknife variational inference. In: Int Conf Learn Represent; 2018.

6. Sharot T. The generalized jackknife: finite samples and subsample sizes. J Am Stat Assoc. 1976;71(354):451–454.

7. Tucker G, Lawson D, Gu S, Maddison CJ. Doubly reparameterized gradient estimators for Monte Carlo objectives. In: Int Conf Learn Represent; 2019.

8. Roeder G, Wu Y, Duvenaud DK. Sticking the landing: simple, lower-variance gradient estimators for variational inference. In: Adv Neural Inf Process Syst; 2017.

9. Archer E, Park IM, Buesing L, Cunningham J, Paninski L. Black box variational inference for state space models. arXiv. 2015;.

10. Burke JV, Ferris MC. A Gauss-Newton method for convex composite optimization. Math Program. 1995;71(2):179–194.

11. Brown EN, Frank LM, Tang D, Quirk MC, Wilson MA. A statistical paradigm for neural spike train decoding applied to position prediction from ensemble firing patterns of rat hippocampal place cells. J Neurosci. 1998;18(18):7411–7425.

12. Arai K, Liu DF, Frank LM, Eden UT. Marked point process filter for clusterless and adaptive encoding-decoding of multiunit activity. bioRxiv. 2018;.

13. Du N, Dai H, Trivedi R, Upadhyay U, Gomez-Rodriguez M, Song L. Recurrent marked temporal point processes: embedding event history to vector. In: KDD; 2016.

14. Glaser JI, Benjamin AS, Chowdhury RH, Perich MG, Miller LE, Kording KP. Machine learning for neural decoding. eNeuro. 2020;7(4).

15. Grosmark AD, Long J, Buzsáki G. Recordings from hippocampal area CA1, PRE, during and POST novel spatial learning; 2016. `http://dx.doi.org/10.6080/K0862DC5`.

16. Daley DJ, Vere-Jones D. An introduction to the theory of point processes. Springer; 2003.

17. Grosmark AD, Buzsáki G. Diversity in neural firing dynamics supports both rigid and learned hippocampal sequences. Science. 2016;351(6280):1440–1443.
